# Supplementary material for: Clinical practice guidelines for acute otitis media in children: a systematic review and appraisal of European national guidelines
Source: BMJ Open. 2020 May 5;10(5):e035343. doi: 10.1136/bmjopen-2019-035343 (PMC7228535; doi:10.1136/bmjopen-2019-035343)
Supplement: Supplementary data [file bmjopen-2019-035343supp001.pdf]

Clinical practice guidelines for acute otitis media in children: A systematic review and appraisal of European national guidelines

Supplementary file 1: Electronic search strategies

a) Medline via Ovid

1	exp Otitis Media/
2	otitis media.tw.
3	acute otitis media.mp.
4	exp Respiratory Tract Infections/
5	aom.mp.
6	middle ear infect*.mp.
7	guideline.mp. or exp GUIDELINE/ or exp PRACTICE GUIDELINE/
8	guide.mp.
9	manage*.mp.
10	exp Clinical Protocols/
11	1 or 2 or 3 or 4 or 5 or 6
12	7 or 8 or 9 or 10
13	11 and 12
14	limit 13 to (yr="2007 - 2017" and "all child (0 to 18 years)")

b) Embase via Ovid

1	1. exp otitis media/
2	otitis media.tw.
3	acute otitis media/
4	exp respiratory tract infection/
5	aom.mp.
6	middle ear infect*.mp
7	exp practice guideline/
8	guide.mp.
9	manage*.mp.
10	exp clinical protocol/
11	1 or 2 or 3 or 4 or 5 or 6
12	7 or 8 or 9 or 10

13	13. 11 and 12
14	14. limit 13 to (yr="2007 - 2017" and child)

c) Cochrane library

	Search term/strategy
1	Exp otitis media
2	Otitis media
3	Glue ear
4	midd ear adj5 (infect* or inflame*)
5	Ome or aom
6	Guideline or practice guideline
7	Management of manage*
8	Clinical protocol
9	#1 or #2 or #3 or #4 or #5
10	#6 or #7 or #8
11	#9 or #10

Results then limited to 16/11/2007-16/11/2017

d) Guidelines International Network (G-I-N)

	Search term/strategy
1	Otitis and
2	Acute Otis media

e) TRIP Medical Database

	Search term/strategy
1	Acute otitis media and
2	Otitis
3	Limit Guidelines
