# Supplementary material for: Clinical practice guidelines for acute otitis media in children: a systematic review and appraisal of European national guidelines
Source: BMJ Open. 2020 May 5;10(5):e035343. doi: 10.1136/bmjopen-2019-035343 (PMC7228535; doi:10.1136/bmjopen-2019-035343)
Supplement: Supplementary data [file bmjopen-2019-035343supp002.pdf]

Clinical practice guidelines for acute otitis media in children: A systematic review and appraisal of European national guidelines

Supplementary File 2: Acute otitis media (AOM) guidelines in Europe

Leading author organisations (Local language)	Leading author organisations (English translation)	Country/ Region	Year published/ updated	Aimed audience	Patient age group	What system used for LoE/SoR?
Institut national d'ass urance maladie-invalidité Comité d'évaluation des pratiques médicales en matière de medicaments (INAMI)	National Institute for Health and Disability Insurance Committee for the Medical Practice on Medicinal Products Evaluation	Belgium	2016	-	Children 0-15 years	Institutional
Odbornáspolečnost praktických dětských lékařů ČLS JEP, Společnost všeobecného lékařství ČLS JEP	CzMA Society of General Practice Society	Czech Republic	2011	-	Children and Adults	None
Dansk Selskab for Almen Medicin (DSAM)	Danish Society for General Practice	Denmark	2014	General Practitioners, Parents and carers	Children 0-5 years	GRADE and Institutional
Suomalainen Lääkäriseura Duodecim	Finnish Medical Society Duodecim	Finland	2017	Primary health care	Children under school age	Institutional
Agence Française de Sécurité Sanitaire des Produits de Santé (AFSSAPS); Agence Nationale de Sécurité du Médicament et des Produits de Santé (ANSM)	French Agency for the Safety of Medicines and Health Products (AFSSAPS) (currently known as National Agency for the Safety of Medicines and Health Products (ANSM)	France	2011	-	Children >3 months of age	National Agency for Accreditation and Evaluation in Health (ANAES)

AWMF (Arbeitsgemeinschaft der wissenschaltlichen medizinischen Fachgesellschaften). Lead society: Deutsche Gesellschaft für Allgemeinmedizin und Familienmedizin (DEGAM)	Association of the Scientific Medical Societies in Germany. Lead society: Society of General Medicine and Family Medicine	Germany	2014	General practitioners, paediatricians, ENT surgeons, junior doctors, audiologists, and primary care workers.	Children and adult (outpatients)	Institutional (AWMF Consensus process S2k)
Health Service Executive and Royal College of Physicians Ireland	-	Ireland	2012	-	Children	None
Società Italiana di Pediatria (SIP) and Società Italiana di Otorinolaringologia Pediatrica	Italian Society of Paediatrics (SIP) and Italian Society of Pediatric Otolaryngology (SIOP)	Italy	2010	Paediatricians, ENT surgeons, general practitioners, nurses, physicians assistants	Children aged 2 months -18 years	Manual for Writing Clinical Practice Guidelines of the Programma Nazionale Linee Guida (PNLG)
Secrétariat du Conseil Scientifique - Domaine de la Santé	Scientific Council of the Ministry of Health	Luxembourg	2007	-	Children >3months of age	None
Nederlands Huisartsen Genootschap (NHG)	Dutch College of General Practitioners	Netherlands	2014	-	Children and adolescents up to 18 years of age	None

Antibiotikasenteret for primærmedisin (ASP)	National Antibiotics Centre for Primary Care	Norway	2016	Physicians, GPs, dentists, private practitioners, medical students	Children and adults	None
Narodowy Instytut Leków (NIL)	National Medicine Institute	Poland	2016	All specialities, including GPs, paediatricians, physicians, Respiratory physicians, ENT surgeons	Children and adults	Infectious Disease Society of America
Departamento da Qualidade na Saúde (DGS)	Department of Health Quality	Portugal	2014	Physicians of the health system	Children	European Society of Cardiology
Asociación Española de Pediatría (AEPED)	Spanish Association of Paediatrics	Spain	2012	-	Children	Infectious Disease Society of America
Läkemedelsverket	Swedish Medical Products Agency	Sweden	2010	-	Children and adults	None
Paediatric Infectious Diseases Group of Switzerland (PIGS)	-	Switzerland	2010	-	Children	None
Scottish Intercollegiate Guidelines Network (SIGN)	-	UK	2003 **Has been retracted by institution**	"All people working with children" including general practitioners (GPs), practice nurses, audiologists,	-	Institutional

				paediatricians, otolaryngologists, audiological physicians, health visitors, social workers, public health physicians, users of services and all other professions caring for children.		
American Association of Paediatrics (AAP)	-	USA	2013	Paediatricians, GPs, Emergency specialists, ENT surgeons, Nurse Practitioners, Physician’s assistants	6 months- 12 years	Institutional
World Health Organisation (WHO)	-	N/A	2013	Doctors, senior nurses, senior health workers	Sick young children in low-resource setting	Institutional
