# Supplementary material for: Clinical practice guidelines for acute otitis media in children: a systematic review and appraisal of European national guidelines
Source: BMJ Open. 2020 May 5;10(5):e035343. doi: 10.1136/bmjopen-2019-035343 (PMC7228535; doi:10.1136/bmjopen-2019-035343)
Supplement: Supplementary data [file bmjopen-2019-035343supp003.pdf]

Clinical practice guidelines for acute otitis media in children: A systematic review and appraisal of European national guidelines:

Supplementary File 3: National guidelines' Level of evidence (LoE) converted to Oxford Centre for Evidence Based Medicine (OCEBM)

**Oxford Centre for EBM Levels of Evidence**		**Belgium**	**Denmark**	**France**	**Finland**	**Italy**	**Norway**	**Poland and Spain**	**Portugal**	**UK SIGN**	**AAP**	**WHO**
1a	SR of RCTs	A/B/C	1a	1	A	I	1a	I	A	1++	A	High
1b	Individual RCT	A/B/C	1b	1	B	I/ II	1b	-	B	1++/ 1+/1-	-	-
1c	All or none	-	1c	-	-	-	-	-	-	-	X	-
2a	SR with homogeneity of cohort studies	-	2a	2	A	III	2a	II	A	2++	B/ C	High-moderate
2b	Individual cohort study	-	2b	-	B/ C	III	2a	-	B	2++/ 2+/2-	-	-
2c	Outcomes research; ecological studies	-	2c	-	C	-	-	-	-	2++/ 2+/2-	-	-
3a	SR with homogeneity of case-control studies	-	3a	3	A	IV	1b, 2a	II	A	2++/2+/2-	B/C	High-moderate
3b	Individual case-control study	-	3b	-	B/C	IV	1b, 2a	-	B	2++/2+/2-	-	-

4	Case series (and poor quality cohort and case-control studies	-	4	4	C	V	3	III	C	3	C/D	Low- very low
5	Expert opinion	-	5	-	D	VI	4	III	C	4	D	-
X	LOE that does not match Oxford	-	DS	-	-	-	2b	-	-	-	-	-
