# Supplementary material for: Clinical practice guidelines for acute otitis media in children: a systematic review and appraisal of European national guidelines
Source: BMJ Open. 2020 May 5;10(5):e035343. doi: 10.1136/bmjopen-2019-035343 (PMC7228535; doi:10.1136/bmjopen-2019-035343)
Supplement: Supplementary data [file bmjopen-2019-035343supp004.pdf]

Clinical practice guidelines for acute otitis media in children: A systematic review and appraisal of European national guidelines:

Supplementary File 4: National guidelines' Strength of recommendation (SoR) converted to OCEBM SoR

Oxford Centre for EBM Strength of Recommendations		Belgium	Denmark	France	Finland	Italy	Norway	Poland and Spain	Portugal	SIGN	AAP	WHO
A	Consistent level 1 studies	-	A	A	A	A/ E	A	A/E	A	A	Strong recommendation/ Option	Strong
B	Consistent level 2 or 3 studies *or* extrapolations from level 1 studies	-	B	B	A/B	-	B	-	B	B	Strong recommendation/ Recommendation/ Option	Conditional /Weak
C	Level 4 studies *or* extrapolations from level 2 or 3 studies	-	C	C	C	-	C	-	C	C	Recommendation/Option	Conditional /Weak
D	Level 5 evidence *or* troublingly inconsistent or inconclusive studies of any level	-	D/ √	C	D	-	D	-	D	D	Option/ No recommendation	No Recommendation
X	*SOR that does not match Oxford CEBM*	1/2	DS	-		B/ C/ D		B/ C/ D	-	-		
