# Supplementary material for: Clinical practice guidelines for acute otitis media in children: a systematic review and appraisal of European national guidelines
Source: BMJ Open. 2020 May 5;10(5):e035343. doi: 10.1136/bmjopen-2019-035343 (PMC7228535; doi:10.1136/bmjopen-2019-035343)
Supplement: Supplementary data [file bmjopen-2019-035343supp005.pdf]

Clinical practice guidelines for acute otitis media in children: A systematic review and appraisal of European national guidelines

Supplementary File 5: Locally defined Strength of Recommendation (SoR) for diagnostic criteria for AOM in Europe and the USA

Guideline	Diagnostic criteria included?	National SoR
Finland	Yes	A
Italy	Yes	A
Poland	Yes	A
Portugal	Yes	I
USA	Yes	Recommendation
Belgium	Yes	No grade
Czech Republic	Yes	No grade
Denmark	Yes	No grade
France	Yes	No grade
Germany	Yes	No grade
Ireland	No	No grade
Luxembourg	Yes	No grade
Netherlands	Yes	No grade
Norway	Yes	No grade
Spain	Yes	No grade
Sweden	Yes	No grade

Switzerland	No	No grade
United Kingdom	Yes	No grade
WHO	Yes	No grade
