# Supplementary material for: Clinical practice guidelines for acute otitis media in children: a systematic review and appraisal of European national guidelines
Source: BMJ Open. 2020 May 5;10(5):e035343. doi: 10.1136/bmjopen-2019-035343 (PMC7228535; doi:10.1136/bmjopen-2019-035343)
Supplement: Supplementary data [file bmjopen-2019-035343supp006.pdf]

## Clinical practice guidelines for acute otitis media in children: A systematic review and appraisal of European national guidelines

Supplementary File 6: Examination tools recommended by European, American and WHO guidelines for acute otitis media (AOM) in children

| Country | Otoscope | Pneumatic otoscope | Tympanometry | Other | National Level of evidence/ strength of recommendation | OCEBM level of evidence/strength of recommendation |
|---|---|---|---|---|---|---|
| Czech Republic | + | | | | - | - |
| Denmark | + | + | + | | - | - |
| Finland | + | + | + | | -/B | -/B |
| France | + | | | | - | - |
| Germany | + | + | + | | - | - |
| Ireland | + | | | | - | - |
| Italy | + | + | + | Mirror | II/ B<br>II/ A | 1b/ X<br>Ib/ A |
| Luxembourg | + | + | | Mirror<br>Ear, Endoscope, Operating microscope | - | - |
| Netherlands | + | | | | - | - |
| Norway | + | + | | | - | - |
| Poland | + | + | + | | A/ II | A/ 2a-2c |
| Portugal | + | | | | - | - |
| Spain | + | + | | | - | - |
| Sweden | + | + | + | | - | - |
| SIGN | + | + | + | | 2+/ - | 2b-3b/ - |
| AAP | + | + | + | | B/<br>Recommendation | 2a-3a/B-C |
| WHO | + | | | | - | - |

No examination tools specified in Belgian and Swiss guidelines
