# Supplementary material for: Clinical practice guidelines for acute otitis media in children: a systematic review and appraisal of European national guidelines
Source: BMJ Open. 2020 May 5;10(5):e035343. doi: 10.1136/bmjopen-2019-035343 (PMC7228535; doi:10.1136/bmjopen-2019-035343)
Supplement: Supplementary data [file bmjopen-2019-035343supp007.pdf]

**Clinical practice guidelines for acute otitis media in children: A systematic review and appraisal of European national guidelines**

Supplementary File 7: Investigations recommended by European, American, and WHO guidelines for acute otitis media (AOM) in children

| **Country** | **Investigation** | **Circumstances** | *National LOE / SOR* | *Oxford LOE /SOR* |
|---|---|---|---|---|
| Czech Republic | • Culture via tympanocentesis or swab<br>• CRP | • Children <2 years of age | - | - |
| Finland | • Culture | • Complications of AOM<br>• Underlying immunodeficiency or significant co-morbidity<br>• Unwell patient requiring hospitalization<br>• To relieve extreme pain | - | - |
| France | • Culture via tympanocentesis | • Treatment failure | - | - |
| Germany | • Blood tests (FBC, CRP, Acute phase protein i.e. interleukin 6/BSG)<br>• Culture via tympanocentesis<br>• Ultrasound or xray | • Complications<br>• Recurrent AOM | - | - |
| Italy | • Blood tests (CRP, ESR, WCC)<br>• Culture via tympanocentesis<br>• Nasopharyngeal aspirate (only quantitatively) | For blood tests:<br>• For follow up<br><br>For tympanocetesis:<br>• Complications<br>• Immunocompromised<br>• Neonate | Typanocentesis: V/ B<br>NPA: IV/B<br>CT scan: IV/A | Tympanocentesis: 4 / X<br>NPA: 3a-3b/X<br>CT scan: 3a-3b/ A |

| | | | | |
|---|---|---|---|---|
| | • CT scan | • Spontaneous otorrhea<br>• Sepsis<br>• Treatment failure<br>• Research purposes | | |
| Luxembourg | • Culture via Tympanocentesis | • Aged <3 months<br>• Severe pain and convex ear on otoscopy<br>• Treatment failure | - | - |
| Norway | • Culture via tympanocentesis or swab of otorrhoea | • Treatment failure | - | - |
| Spain | • Blood tests (FBC, CRP, blood culture)<br>• Culture (Via tympanocentesis or swab of otorrhea )<br>• Lumbar puncture<br>• Skull/Temporal bone CT | • Complications<br>• Recurrent AOM<br>• Spontaneous otorrhea<br>• Treatment failure | - | - |
| Sweden | • Culture via tympanocentesis<br>• Nasopharyngeal swab | • Treatment failure | - | - |
| AAP | • Culture via tympanocentesis<br>• Nasopharyngeal aspirate | • Treatment failure | - | - |

No Investigations advised: Belgium, Denmark, Ireland, Netherlands, Poland, Portugal, Switzerland, UK SIGN, WHO
