# Supplementary material for: Clinical practice guidelines for acute otitis media in children: a systematic review and appraisal of European national guidelines
Source: BMJ Open. 2020 May 5;10(5):e035343. doi: 10.1136/bmjopen-2019-035343 (PMC7228535; doi:10.1136/bmjopen-2019-035343)
Supplement: Supplementary data [file bmjopen-2019-035343supp008.pdf]

Clinical practice guidelines for acute otitis media in children: A systematic review and appraisal of European national guidelines

Supplementary File 8: Antibiotic treatment recommended by European, American and WHO guidelines for acute otitis media (AOM) in children

Guideline	First line*	Duration of first line therapy	Second line/Treatment failure	Third line/ Allergy to first line	National LOE / SOR	Oxford LOE /SOR
Belgium	PO amoxicillin 75mg-100/kg/day in 3 divided doses	7 days	Treatment failure :<br><br>cefuroxime axetil 30-50 mg / kg in 3 doses<br><br>amoxicillin-clavulanic acid 50 / 37.5 mg / kg in 3 doses	In case of Allergy to cephalosporins , either: Co-trimoxazole (Trimethoprim 8 mg/kg/day and Sulfumethoxazole 40 mg/kg/day) in 3 divided doses<br><br>Levofloxacin 10 mg/ kg/day in 2 divided doses	For treatment choice: Expert Opinion/Weak recommendati on<br><br>Duration: GRADE A - B, low recommendati on)	For treatment choice: 5 /X<br><br>For duration: 1a-1b/ X
Czech Republic	PO amoxicillin 75-90mg/kg/day in 3 divided doses	7-10 days	Discusses targeted therapy to specific bacterial agents, but no empiric antibiotic for treatment failure	Allergy to beta lactams: co-trimoxazole	None	None
Denmark	PO Penicillin V 60mg/kg/day in 3 divided doses	7 days	Treatment failure<br><br><2 years of age: Amoxicillin-clavulanic acid	Penicillin allergy: clarithromycin 7.5mg.kg/dose x2 for 7 days	Level 1a-1c and 5 / Grade A and √	Level 1a-1c and 5

			10/2.5mg/kg/dose 8 hourly for 7 days<br><br><u>2-12 years of age:</u> amoxicillin – clavulanic acid 1-/2.5mg/kg/dose 8 hourly for 7 days		For duration: No LOE/SOR	
Finland	PO Amoxicillin 40mg/kg/day 8-12 hourly<br>OR<br>PO amoxicillin-clavulanic acid 40/5.7mg/kg/day in 2-3 divided doses	5 -7 days	<u>If vomiting</u>: IM Ceftriaxone (one dose)	<u>Penicillin allergy:</u> cefaclor, cefuroximexetil, sulfa trimethoprim, azithromycin or clarithromycin	First line: C / C<br><br>(None for treatment duration)	1b, 2b-2c, and 3b-4
France	PO amoxicillin 80-90mg/kg/day in 2-3 divided doses	5 day duration if >2 years of age, 8-10 days if <2 years of age	<u>Treatment failure:</u> PO Amoxicillin/clavulanic acid 80mg/kg/day) AND PO amoxicillin 70mg/kg/day)<br><br>OR<br><br>IM/IV ceftriaxone 50mg/kg daily for 3 days<br><br>PO Amoxicillin/clavulanic acid = otitis conjunctivitis syndrome	Allergy to beta lactams erythromycin-sulfafuraole or cotrimoxazole<br><br>Allergy to penicillins without allergy to cephalosporins: cefpodoxime	Only duration has LOE:<br><br>Level 1 and Professional Agreement/ Grade A<br><br>Duration for child <2 years of age is Level 1/ Grade A<br>>2 years of age is professional agreement (No LOE)<br><br>3rd line treatment: Professional agreement (No LOE)	Level 1a

Germany	PO Amoxicillin 50mg/kg/day in 2-3 divided doses<br><br>If from country with high rates of penicillin resistance: PO amoxicillin 80-90mg/kg/day	7 days	Treatment failure: PO amoxicillin 80-90mg/kg/day<br><br>Second choice: PO cephalosporin including cefuroxime axetil (20-30mg/kg/day for 5 days),	Allergy to penicillins/cephalosporins: macrolide ie erythromycin 7 days	None	None
Ireland	Amoxicillin (no duration, no route, no frequency)	None	None	None	None	None
Italy	Mild symptoms and no otorrhea nor risk factors† :<br>PO Amoxicillin 50mg/kg/day in 2-3 divided doses<br><br>Severe symptoms, otorrhea, or risk factors for bacterial resistance<br>Amoxicillin-clavulanic acid 80-90mg/kg/day in 2-3 divided doses	Duration:<br><br>10 days <2 years of age<br>5 days >2 years of age	Treatment failure:<br>If they were treated with amoxicillin or cefaclor: amoxicillin plus clavulanic acid or cefpodoxime proxetil or cefuroxime axetil.<br><br>If they were being treated with a broad-spectrum antibiotic: intramuscular or intravenous ceftriaxone 50mg/kg once daily<br><br>Duration:<br>10 days <2 years of age or spontaneous otorrhea<br>5 days >2 years of age<br>3 days for ceftriaxone	Penicillin allergy: macrolide	First line (for both) : Level I/ Grade A<br><br>Treatment failure: Level II/ Grade B<br><br>Duration Level I/ Grade B<br><br>Allergy:<br>VI/D	First line:<br>Level1a- 1b/ A<br><br>Treatment failure: Level 1b/ X<br><br>Duration:<br>Level 1a-1b /X

Luxembourg	Amoxycillin 80-90mg /kg/day in 3 divided doses<br><br>Alternatively if 'very severe cases'<br><br>Amoxicillin-clavulanic acid 80-90mg/kg/day in 3 divided doses	<6 years of age 10 days treatment<br><br>>6 years 5-7 days treatment	Treatment failure:<br><br>Amoxycillin/ clavulanate<br><br>Otherwise<br><br>cefuroxime axetil no dose, no frequency) or<br><br>ceftriaxone 50mg/kg/day for 3 days<br><br>or<br><br>azithromycin or clarithromycin or clindamycin	If vomiting: Ceftriaxone 50 mg / kg once daily for 3 days<br><br>Penicillin allergy: Cefuroxime 30mg/kg in two divided doses<br><br>Allergy to penicillin + cephalosporin: Azithromycin 10mg/kg/day for 6 days or clarithromycin 15mg/kg/day in two divided doses<br><br>Otherwise: sulfamethoxazole-trimethoprim 6019ng.kg of trimethoprim per day or clindamycin 30-40mg/kg in three divided doses	Not applicable	None<br>Not applicable
Netherlands	Amoxicillin 40mg/kg/day in 3 divided doses	7 days	Second line and also treatment failure:<br><br>Amoxicillin-clavulanic acid 40/10mg/kg/day in 3 divided doses for 7 days	Penicillin allergy: cotrimoxazole 36mg/kg/day in two divided doses; 5-7 days	Not applicable	Not applicable
Norway	PO phenoxymethylpenicillin 24-60mg/kg/day in 3-4 divided doses per day<br><br>In case of frequent/recurent cases:	5 days	Treatment failure:<br><br>trimetroprim sulfamethoxazole (for children)	Allergy:<br><25kg:<br>Erythromycin oral solution (ethyl succinate) 40mg/kg/day in two divided doses for 5 days<br><br>or<br>Clarithromycin (children over 6 months) 15 mg/kg/day in two divided doses for 5 days	None	None

	Amoxicillin 21-42mg/kg/day in three divided doses			25-35kg:<br>Erythromycin enteric capsules 500mg/kg/day in two divided doses for 5 days<br>or<br>Clarithromycin 14 mg/kg/day in two divided doses for 5 days		
Poland	PO amoxicillin<br><40kg: 75-90mg/kg/day in 2 divided doses<br><br>>40kg 3000-4000mg per day in 2 divided doses	If <2 years of age, 10 days duration<br><br>If >2 years of age, 5 days duration	Treatment failure:<br>Amoxicillin/clavulanic acid<br><40kg: 70-90mg/kg/day in two divided doses<br><br>>40kg: 3000mg-4000mg per day in 2 divided doses<br><br>IVceftriaxone<br><40kg 50mg/kg/day once daily for 3 days<br><br>>40kg 1-2g/day once daily for 3 days	Allergy to amoxicillin:<br>PO Cefuroxime axetil<br>>40kg 1000mg/day in 2 divided doses for 5 days<br><40kg 30mg/kg/day in two divided doses for 5 days unless if <2 yoa then for 10 days<br><br>Allergy to amoxicillin and severe infection:<br>Ceftriaxone<br>>40kg- 1-2g IV/IM per day once daily for 3 days<br><40kg- 50mg/kg IV/IM per day once daily for 3 days<br><br>Allergy to beta lactams:<br>Clarithromycin<br><40kg 15-20mg/kg/day in two divided doses;<br>>40kg 500-1000mg/day in two divided doses	First line:<br>Level II /Grade A<br><br>First line duration: Level II /Grade B<br><br>Treatment failure:<br>Level II-III/ Grade A-B<br><br>Allergy treatment:<br>Level I-II/ Grade A-B	First line antibiotic:<br>Level 2a and 3a<br><br>First line duration:<br>Level 2a and 3a<br><br>Treatment failure: Level 2a, 3a, 4, and 5<br><br>Allergy treatment:<br>1a, 2a, 3a

Portugal	Amoxicillin 80-90mg/day in 2 divided doses	5 days routine or <2 years of age<br>7 days if <2 years, recurrent, failure of initial treatment<br>10 days if recurrent AOM	Treatment failure:<br><br>PO/IV Amoxicillin and clavulanic acid 80-90mg/kg/day in 2 divided doses or<br><br>PO Cefuroxime-axetil 30mg/kg/day in 2 divided doses or IV 80-100mg/kg/day in 3 dividied doses<br>7 days if <2 years<br><br>OR<br><br>IM/IV Ceftriaxone 50mg/kg/day once daily	Penicillin allergy:<br>Clarithromycin 50mg/kg/day in 2 divided doses or<br><br>Erythromycin 50mg/kg/day in 3-4 divided doses per day or<br><br>Azithromycin 10mg/kg/day once a day	First line:<br>Level A/ Grade 1<br><br>Treatment failure: Level B/ Grade IIa<br><br>Duration:<br>7 days- Level A / Grade IIa<br><br>5 days- Level A/ Grade 1<br><br>10 days no evidence<br><br>Allergic treatment:<br>Level C/Grade I	First line: level 1a, 2a, or 3a<br><br>Treatment failure: Level 1b, 2b, or 3b<br><br>Duration:<br>7 days- level 1a, 2a, or 3a<br><br>5 days- level 1a, 2a, or 3a<br><br>10 days no evidence<br><br>Allergic treatment Level 4-5 s
Spain	PO Amoxicillin 80-90mg/kg/day in 3 divided doses<br><br>If <6 months, severe symptoms, family history of ENT complications, previous therapeutic failure<br>Amoxicillin/clavulanic acid 80-90mg/kg/day in 3 divided doses for 7-10 days<br><br><br>If <2 months of age:	Routine: 5 days<br><br>Otherwise: 10 days	Treatment failure:<br><br>Amoxicillin-clavulanic acid 80-90mg/kg/day in 3 divided doses for 7-10 days or IM/IV Ceftriaxone 50mg/kg/day for 3 days	Penicillin allergy:<br>Cefuroxime axetil 30mg/kg/day in 2 divided doses<br><br>If anaphylaxis to penicillin:<br>Clarithromycin 15mg/kg/day in 2 divided doses for 7 days or azithromycin 10mg/kg once daily on first day, then 5mg/kg once daily for 4 additional days.<br>Can also give Levofloxacin<br>6 months-5 yoa: 10mg/kg every twelve hours<br><br>>5 yoa give 10mg/kg every 24 hours	First line:<br>Level II/ Grade B<br><br>For children requiring amoxicillin-clavulanic acid as first line:<br>Level II/ Grade B (No evidence for <2 months of age)<br><br><br>Treatment failure:<br>Level III/Grade C; for	First line: 2a<br><br>For children requiring amoxicillin-clavulanic acid as first line: Level 2a-3a<br><br>Treatment failure: Level 4-5 ;<br>Ceftraixone as used in treatment failure: Level 1a

	IV cefotaxime or PO/ IV amoxicillin-clavulanic acid. For PO only if no fever or no symptoms. No LOE				ceftriaxone Level I/Grade A<br><br>Allergic to penicillin/cephalosporin: Level III/Grade C	Allergy to penicillin/cephalosporin: Level 4-5
Sweden	PO Penicillin V 75 mg / kg/day in 3 divided doses<br><br>If recurrent: (new acute otitis media within a month with symptom-free intervals): Penicillin V 75mg / kg/day in 3 divided doses or<br><br>Amoxicillin 60 mg / kg/day in 3 divided doses	Routine: 5 days<br><br>Recurrent: 10 days	Treatment failure:<br><br>Amoxycillin 60mg/kg/day in 3 divided doses for ten days.	Penicillin allergy: Erythromycin 40mg/kg/day in 4 divided doses or 40mg/kg/day in 2 divided doses	Not applicable	Not applicable
Switzerland	Amoxicillin 50mg/kg/day in 2 divided doses<br><br>If risk factors or from country with high rates of penicillin resistance: Amoxicillin 80mg/kg/day in 2 divided doses	Routine: 5 days<br><br><2 years of age, previous otitis media, perforated tympanic membrane or from country with high rates of penicillin resistance : 10 days	‡Amoxicillin/clavulanic acid 80mg/kg/day in two divided doses for 10 days ‡ or<br><br>Ceftriaxone 50mg/kg daily for 1-3 days	None	Not applicable	Not applicable
UK SIGN (refers to BNF for Children)	Amoxicillin	5-7 days	Treatment failure: Amoxicillin-clavulanic acid	Penicillin allergy:	None	None

	Child 1-11 months of age: 125mg three times a day for 5-7 days<br><br>Child 1-4 years: 250mg three times a day for 5-7 days<br><br>(NB: dosage can be both low and high dose amoxicillin dependently on kg; for example If 1 yoa and weight <50th centile, will be given high dose; if 2 yoa and weight at 50th centile, would be given low dose.		(dose dependent on suspension available)	Clarithromycin or erythromycin ; dosage dependent on age		
USA	Amoxicillin (80–90 mg/ kg/ day in 2 divided doses<br><br>OR<br><br>If amoxicillin past 30 days, concurrent purulent conjunctivitis (otitis conjunctivitis syndrome), or history of AOM non responsive to amoxicillin:<br><br>Amoxicillin-clavulanic acid (90 mg/kg/ day amoxicillin dosage in 2 divided doses	<2 years of age: 10 days<br><br>2-5 years of age:7 days<br><br>>6 years of age 5-7 days	Treatment failure:<br><br>Amoxicillin-clavulanic acid 90mg/kg/day 12 hourly<br><br><2 years of age: 10 day course<br><br>2-5 years of age 7 day course<br><br>>6 years of age 5-7 days<br><br>OR<br><br>IM/IV Ceftriaxone 50mg daily 3 days	Penicillin allergy:<br><br>Cefdinir 14mg/kg per day in 1-2 divided doses or cefuroxime 30mg/kg per day in 2 divided doses or cefpodoxime 10mg/kg per day in 2 divided doses or ceftriaxone 50mg IM or IV per day for 1-3 days	First line : B, recommendation; first line amoxil-clav: C, recommendation<br><br>Treatment failure: B, recommendation	First line: 2a or 3a<br><br>First line amoxi clav: 2a, 3a, 4<br><br>Treatment failure: 2a, 3a

WHO	PO amoxicillin 80mg/kg/day in 2 divided doses<br><br>If consider pathogen sensitive, give co-trimaxazole, dose: (trimethoprim component 8mg/kg/day 12 hourly for 5 days)	7-10 days	Repeat antibiotics for another 5 days	None	Low quality evidence/ Strong recommendation	Level 4

NB Amoxicillin-clavulanic acid dose always given in terms of amoxicillin component dosage.

* Routinely to treat with low-dose amoxicillin, but for high dose amoxicillin in the following situations: Switzerland: If from area of high penicillin resistance, age <6 months, severe symptoms. UK: “If necessary.” Germany: area of high penicillin resistance
† Italy risk factors: risk factors for bacterial resistance: age <3 years, day-care attendance, older siblings, recent antibiotic therapy (<1 month), no PCV-7.
‡ Switzerland: If <6 months, severe symptoms, family history of ENT complications, previous therapeutic failure, recent administration of amoxicillin, concurrent purulent conjunctivitis or otorrhoea , region of high penicillin resistance or risk factors for antibiotic resistant for PO amoxicillin-clavulanic acid as first line.
