# Supplementary material for: Clinical practice guidelines for acute otitis media in children: a systematic review and appraisal of European national guidelines
Source: BMJ Open. 2020 May 5;10(5):e035343. doi: 10.1136/bmjopen-2019-035343 (PMC7228535; doi:10.1136/bmjopen-2019-035343)
Supplement: Supplementary data [file bmjopen-2019-035343supp009.pdf]

Clinical practice guidelines for acute otitis media in children: A systematic review and appraisal of European national guidelines

Supplementary File 9: AGREE scores for acute otits media guidelines (AOM) in European, American, and WHO guidelines

a) National AGREE scores (%)

**Guideline**	**Domain 1**	**Domain 2**	**Domain 3**	**Domain 4**	**Domain 5**	**Domain 6**	**Mean National Score**
Belgium	100	56	64	81	10	29	57
Czech Republic	36	25	4	21	0	0	14
Denmark	97	64	83	100	56	54	76
Finland	92	50	59	97	40	42	63
France	64	0	3	83	4	0	26
Germany	83	61	43	92	35	96	68
Ireland	11	3	1	56	2	0	12
Italy	100	83	63	97	40	46	72
Luxembourg	58	6	6	58	4	0	22
Netherlands	69	81	74	92	25	83	71
Norway	53	50	14	86	13	0	36
Poland	67	42	27	92	21	13	44
Portugal	44	33	18	64	33	8	33
Spain	44	28	22	83	15	29	37
Sweden	64	25	9	83	31	58	45
Switzerland	8	0	0	56	0	0	11
UK SIGN	92	92	82	92	58	29	74

European mean	*64*	*41*	*34*	*78*	*23*	*29*	*29*
AAP	97	67	88	89	35	54	72
WHO	94	58	80	92	60	83	78

b) AGREE scores by marker
    i) HS AGREE scores

	**Domain 1**			**Domain 2**			**Domain 3**								**Domain 4**			**Domain 5**				**Domain 6**	
Criteria	**1**	**2**	**3**	**4**	**5**	**6**	**7**	**8**	**9**	**10**	**11**	**12**	**13**	**14**	**15**	**16**	**17**	**18**	**19**	**20**	**21**	**22**	**23**
Belgium	7	7	7	4	3	4	7	7	7	6	5	7	1	1	6	7	5	1	4	1	1	2	3
Czech Republic	7	1	1	6	1	1	1	1	1	1	1	1	3	1	7	4	7	1	1	1	1	1	1
Denmark	6	7	7	5	1	6	7	6	7	5	7	6	7	5	7	7	7	3	7	5	7	5	7
Finland	7	6	6	6	1	6	7	5	7	6	6	5	5	5	6	7	7	7	6	6	4	5	7
France	3	3	5	1	1	1	1	1	1	1	3	1	1	1	5	5	5	2	2	1	1	1	1
Germany	6	5	5	5	1	7	1	5	6	2	6	5	1	7	7	6	6	6	4	5	4	7	7
Ireland	1	1	1	1	1	1	1	1	1	1	2	1	1	1	5	4	6	1	1	1	2	1	1
Italy	7	7	7	7	7	7	7	6	5	6	6	6	6	4	6	7	7	6	5	5	3	5	6
Luxembourg	2	2	7	1	1	1	1	1	1	1	1	1	1	1	5	4	4	3	1	1	1	1	1
Netherlands	3	2	7	6	6	6	7	7	7	5	7	5	7	1	7	6	7	6	4	5	1	7	7
Norway	7	1	4	5	1	7	1	1	1	1	1	4	3	3	6	7	7	3	3	1	2	1	1
Poland	5	4	2	3	1	7	4	1	5	1	6	4	1	1	6	6	7	5	4	4	1	1	1
Portugal	2	1	7	1	1	4	1	1	2	1	2	3	1	4	4	3	3	3	5	2	6	1	3
Spain	4	2	2	5	2	1	1	2	3	1	4	7	1	1	6	6	5	5	2	3	1	1	5
Sweden	6	5	7	6	1	1	1	1	5	1	5	1	1	1	5	7	7	7	5	4	1	5	7
Switzerland	1	1	1	1	1	1	1	1	1	1	1	1	1	1	5	3	6	1	1	1	1	1	1

UK SIGN	6	5	7	6	1	7	7	6	6	7	7	6	7	5	6	6	6	7	5	5	7	3	3
AAP	7	7	7	7	1	7	6	7	6	7	6	7	6	5	5	6	7	5	6	6	2	1	7
WHO	7	7	5	5	1	7	7	5	7	5	6	5	7	6	6	5	7	6	7	7	2	6	7

ii) JED AGREE Scores

	**Domain 1**			**Domain 2**			**Domain 3**								**Domain 4**			**Domain 5**				**Domain 6**	
**Criteria**	**1**	**2**	**3**	**4**	**5**	**6**	**7**	**8**	**9**	**10**	**11**	**12**	**13**	**14**	**15**	**16**	**17**	**18**	**19**	**20**	**21**	**22**	**23**
Belgium	7	7	7	6	4	5	7	7	7	4	4	5	1	1	6	7	4	1	1	1	3	3	3
Czech Republic	5	1	4	5	1	1	1	1	1	1	1	2	2	1	4	7	7	1	1	1	1	1	1
Denmark	7	7	7	7	3	7	7	7	6	1	7	6	5	6	7	7	7	1	6	1	5	1	4
Finland	7	6	7	6	1	4	5	1	5	1	1	7	1	6	7	7	7	1	1	1	1	1	1
France	7	4	7	1	1	1	1	1	1	1	2	1	1	1	7	7	7	1	1	1	1	1	1
Germany	6	7	7	6	2	7	1	1	1	6	5	1	2	7	6	7	7	1	2	2	1	6	7
Ireland	3	2	2	1	1	2	1	1	1	1	1	1	1	1	2	2	7	1	1	1	1	1	1
Italy	7	7	7	7	1	7	4	2	4	1	4	6	3	6	7	7	7	1	4	2	1	1	3
Luxembourg	5	4	7	3	1	1	1	1	1	1	5	3	1	1	6	7	5	1	1	1	1	1	1
Netherlands	6	7	6	6	4	7	7	7	7	4	6	5	4	1	6	7	6	1	1	1	1	5	5
Norway	6	2	5	5	1	5	1	1	1	1	1	2	2	5	5	7	5	1	3	1	1	1	1
Poland	7	5	7	3	1	6	2	1	1	1	5	7	1	1	6	7	7	1	1	1	1	4	1
Portugal	4	1	7	5	1	6	1	1	1	1	2	5	1	6	5	7	7	1	1	1	5	1	1
Spain	5	4	5	6	1	1	1	1	1	1	5	6	1	1	7	7	5	1	1	1	1	1	4
Sweden	4	3	4	5	1	1	1	1	1	2	1	1	1	1	3	7	7	3	1	1	1	1	5
Switzerland	2	3	1	1	1	1	1	1	1	1	1	1	1	1	2	5	5	1	1	1	1	1	1

UK SIGN	7	7	7	7	4	7	4	3	7	6	7	6	5	6	7	7	7	2	3	1	6	1	4
AAP	6	7	7	7	1	7	5	6	7	7	7	7	6	5	6	7	7	7	1	2	1	3	6
WHO	7	7	7	6	1	7	4	1	7	7	7	7	6	6	7	7	7	5	4	4	2	4	7
