# Supplementary material for: Clinical practice guidelines for acute otitis media in children: a systematic review and appraisal of European national guidelines
Source: BMJ Open. 2020 May 5;10(5):e035343. doi: 10.1136/bmjopen-2019-035343 (PMC7228535; doi:10.1136/bmjopen-2019-035343)
Supplement: Author's manuscript [file bmjopen-2019-035343.draft_revisions.pdf]

BMJ Open

BMJ Open is committed to open peer review. As part of this commitment we make the peer review history of every article we publish publicly available.

When an article is published we post the peer reviewers' comments and the authors' responses online. We also post the versions of the paper that were used during peer review. These are the versions that the peer review comments apply to.

The versions of the paper that follow are the versions that were submitted during the peer review process. They are not the versions of record or the final published versions. They should not be cited or distributed as the published version of this manuscript.

BMJ Open is an open access journal and the full, final, typeset and author-corrected version of record of the manuscript is available on our site with no access controls, subscription charges or pay-per-view fees (http://bmjopen.bmj.com).

If you have any questions on BMJ Open's open peer review process please email info.bmjopen@bmj.com

BMJ Open

Clinical practice guidelines for acute otitis media in children: A systematic review and appraisal of European national guidelines

Journal:	*BMJ Open*
Manuscript ID	bmjopen-2019-035343
Article Type:	Original research
Date Submitted by the Author:	04-Nov-2019

Complete List of Authors:	Suzuki, Hijiri; London School of Hygiene and Tropical Medicine Department of Clinical Research, l;<br>Dewez, Juan ; London School of Hygiene and Tropical Medicine, Clinical Research Department<br>Nijman, R; Imperial College London,<br>Yeung, Shunmay; London School of Hygiene and Tropical Medicine

Keywords:	Protocols & guidelines < HEALTH SERVICES ADMINISTRATION & MANAGEMENT, INFECTIOUS DISEASES, Paediatric otolaryngology < OTOLARYNGOLOGY, PAEDIATRICS, PUBLIC HEALTH

SCHOLARONE™
Manuscripts

I, the Submitting Author has the right to grant and does grant on behalf of all authors of the Work (as defined in the below author licence), an exclusive licence and/or a non-exclusive licence for contributions from authors who are: i) UK Crown employees; ii) where BMJ has agreed a CC-BY licence shall apply, and/or iii) in accordance with the terms applicable for US Federal Government officers or employees acting as part of their official duties; on a worldwide, perpetual, irrevocable, royalty-free basis to BMJ Publishing Group Ltd ("BMJ") its licensees and where the relevant Journal is co-owned by BMJ to the co-owners of the Journal, to publish the Work in this journal and any other BMJ products and to exploit all rights, as set out in our licence.

The Submitting Author accepts and understands that any supply made under these terms is made by BMJ to the Submitting Author unless you are acting as an employee on behalf of your employer or a postgraduate student of an affiliated institution which is paying any applicable article publishing charge ("APC") for Open Access articles. Where the Submitting Author wishes to make the Work available on an Open Access basis (and intends to pay the relevant APC), the terms of reuse of such Open Access shall be governed by a Creative Commons licence – details of these licences and which Creative Commons licence will apply to this Work are set out in our licence referred to above.

Other than as permitted in any relevant BMJ Author's Self Archiving Policies, I confirm this Work has not been accepted for publication elsewhere, is not being considered for publication elsewhere and does not duplicate material already published. I confirm all authors consent to publication of this Work and authorise the granting of this licence.

For peer review only

TITLE PAGE

Title: Clinical practice guidelines for acute otitis media in children: A systematic review and appraisal of European national guidelines

Authors: Hijiri G Suzuki, Juan Emmanuel Dewez, Ruud G Nijman, Shunmay Yeung

Correspondence to:
Shunmay Yeung:
London School of Hygiene & Tropical Medicine, Keppel Street, London WC1E 7HT
Email: Shunmay.Yeung@lshtm.ac.uk Phone number: +44 2079272657

Affiliations:
Hijiri G Suzuki (HGS):
1. London School of Hygiene and Tropical Medicine, Clinical Research Department, Faculty of Infectious and Tropical Diseases, Keppel St, Bloomsbury, London WC1E 7HT, United Kingdom

Juan Emmanuel Dewez (JED):
1. London School of Hygiene and Tropical Medicine, Clinical Research Department, Faculty of Infectious and Tropical Diseases, Keppel St, Bloomsbury, London WC1E 7HT, United Kingdom

Ruud G Nijman (RGN):
1. Section of Pediatrics, Division of Infectious Diseases, Department of Medicine, Imperial College London, Norfolk Place, London, W2 1PG, United Kingdom

Shunmay Yeung (SY):
1. MARCH Centre for Maternal, Adolescent, Reproductive and Child Health, London School of Hygiene and Tropical Medicine, Clinical Research Department, Faculty of Infectious and Tropical Diseases, Keppel St, Bloomsbury, London WC1E 7HT, United Kingdom
2. Section of Pediatrics, Division of Infectious Diseases, Department of Medicine, Imperial College London, Norfolk Place, London, W2 1PG, United Kingdom

Key words:
Acute Otitis media
Europe
Guidelines
Children
Systematic review
Antibiotic stewardship

Main text word count:
2,240 (excluding abstract, references, tables, summary boxes, acknowledgements, transparency declaration, funding, competing interests, authorship)

Supplemental Files:

- Appendix
- Four Manuscript Figures
- PRISMA Checklist
- Summary Protocol

For peer review only

Summary Boxes

Section 1: What is already known on this topic

- AOM is one of the most common infections in children and young people accounting for a large proportion of antibiotic prescription in children
- Clinical guidelines are important tools for improving patient care and antibiotic stewardship to reduce antibiotic resistance

Section 2: What this study adds

- There are significant similarities in choice, duration, and indications for antibiotic treatment between European AOM guidelines
- The majority of guidelines do not refer to local antibiotic resistance patterns
- Centrally developed and locally adapted guidelines may provide more targeted recommendations and reduce unnecessary antibiotic administration

For peer review only

ABSTRACT (WORD COUNT: 285)

Objective

To appraise European guidelines for acute otitis media (AOM) in children, including assessing their methodologic quality, describing their evidence-based strength of recommendations (SoR), and assessing whether they include considerations of antibiotic stewardship.

Methods

Design:

A systematic literature review using five search engines, websites of European national paediatric associations and expert contacts to identify national guidelines on the management of AOM in children.

Setting:

European Union and European Free Trade Association countries

Participants:

Children aged <16 years with AOM.

Interventions:

Guidelines were graded using AGREE II criteria. Strength of Recommendations (SoR) of guidelines were compared. Guidelines were assessed for antibiotic stewardship.

Primary outcome measures

Quality of AOM guidelines in Europe and recommendations for antibiotics prescribing

Secondary outcome measures

Strength of recommendations and inclusion of antibiotic stewardship principles

Results

AOM guidelines were obtained from 17 of the 32 EU/EFTA countries. AGREE scores varied between guidelines, with mean scores of less than 40% for most domains. Diagnosis of AOM was based upon similar signs and symptoms across guidelines. The majority (15/17; 88%) described a watchful waiting approach to antibiotic therapy. Amoxicillin was the most common first-line antibiotic agent (14/17; 82%), with treatment duration of five to ten days. Seven countries advocated high dose (75-90mg/kg/day) and five countries low dose (30-60mg/kg/day) of amoxicillin. The most common indication for antibiotics was symptom severity (12/15; 80%) and bilateral infections in infants less than 24 months of age (11/15; 73%). Under half of the guidelines (7/17; 41%) referred to local microbiological and antibiotic resistance data.

Conclusions

The guidelines for managing AOM were similar across European countries. Guideline quality was mostly weak, and lacked consideration of local antibiotic resistance patterns. Co-ordinating efforts to produce a core guideline which can then be adapted by each country may help improve overall guideline quality, helping to tackle antibiotic resistance.

ARTICLE SUMMARY: Strengths and Limitations of this study

For peer review only

- Strengths include comprehensive three-tiered search strategy to identify a large number of guidelines in all European languages
- Usage of AGREE-II, a standardized and internationally recognised guideline appraisal tool.
- Our analysis included an assessment of SoR and whether antibiotic stewardship, a key measure to reduce AMR, was included.
- As the heterogeneity of level of evidence (LoE) across countries limited comparison we decided to compare strength of recommendations (SoR) instead, which is easier but provides weaker information about the quality of evidence.

For peer review only

INTRODUCTION

Acute otitis media (AOM) is one of the commonest infections of childhood;[1 2] approximately 60% of children have had at least one episode by four years of age.[3] It is also one of the most frequently cited reasons for antibiotic prescription in children less than 3 years of age [4 5], accounting for 14% of all antibiotic prescriptions in children in the UK. [6]

Although bacteria are frequently present in the middle ear,[3, 7, 8] studies indicate that approximately half of all patients with AOM may get better without antibiotic treatment.[9 10] The rationale for antibiotic prescription includes symptom control,[11] and the prevention of rare but serious complications, including mastoiditis and meningitis.[12] However, antibiotics are associated with the risk of side effects including vomiting, diarrhoea, and rash.[13] In addition, from a public health perspective the inappropriate use of antibiotics has been identified as one of the key drivers of antibiotic resistance, an emerging global health priority.[5, 14 15, 16] Emerging research has also demonstrated that longer antibiotic courses lead to higher risks of resistance. Thus, providing clear guidance on appropriate antibiotic use in terms of the indications, choice and duration is considered important to help reducing antibiotic resistance.[17]

To promote antibiotic stewardship, the World Health Organization recommends the development of treatment guidelines and the monitoring of local antibiotic resistance to inform the choice of antibiotics.[18] National guidelines for the first-line management of AOM may play a vital role in antibiotic stewardship.[19] To our knowledge there has not been a systematic review of the quality and content of national guidelines for the management of AOM. The aim of this systematic review was to describe European guidelines for AOM in children, to assess their methodologic quality, to describe their evidence-based strength of recommendations, and to assess whether they incorporate consideration of antibiotic stewardship.

METHODOLOGY

To ensure a comprehensive review of nationally endorsed guidelines, we used a three-pronged approach that included (1) a systematic database search; (2) a website search of national societies; and (3) expert consultation.

For peer review only

Firstly, a systematic search of databases was carried out using Medline via Ovid, Embase, Cochrane library, Guidelines International Network (G-I-N), and Trip Medical Database in April 2017. Search terms were a combination of two elements 1) Synonyms for "acute otitis media" AND 2) Synonyms for guidelines. Guidelines were included if they met the following eligibility criteria: 1) were pertaining to the management of simple AOM, excluding the management of chronic or complex otitis media cases requiring specialist (Ear Nose Threat specialist) input; 2) they were national guidelines or endorsed by the national medical society from a European Union (EU) or European Free Trade Area (EFTA) country; and 3) published from the year 2000 to present. The American Association of Paediatrics (AAP) and the WHO guidelines were also included for comparison. The search included all European languages. Additionally, the bibliographies of all guidelines were examined to identify further relevant resources (HGS). An initial review of titles and abstracts was performed by one reviewer (HGS).

Secondly, the websites of national paediatric associations listed by the European Paediatric Association/Union of National European Paediatric Societies and Associations (EPA/ UNEPSA) were hand searched (HGS). Finally, a network of research partners across Europe were contacted (RGN, SY, JED, HGS) to verify if the identified guidelines were the most up to date and widely utilised, and in cases where we had not managed to locate any guidelines, to assist in obtaining them. The choice of search terms and final selection of full-text guidelines was performed by two reviewers (HGS, JED) (Appendix 1-2). If multiple national guidelines were found, the guideline judged to be most up to date, comprehensive, and more commonly utilised in clinical practice was included after discussion between research partners and reviewers (HGS, JED). Data was extracted using tables constructed by the research team.

Patient and Public Involvement

This research was performed without patient involvement.

Guideline Quality Assessment

The AGREE II Instrument was used independently by two reviewers (HGS, JED) to determine the quality of each national guideline.[20] This is a standardised instrument

For peer review only

that appraises the methodologic framework of guideline development. The six domains assessed are 1) Scope and purpose 2) Stakeholder involvement 3) Rigour of development including evidence base 4) Clarity of presentation 5) Applicability and 6) Editorial independence. Domains were scored on a 0 – 10 scale; any score that varied by >3 out of 7 was discussed and revised if this was felt to be reasonable.

Level of evidence and Strength of recommendation

To allow for meaningful comparison of the evidence upon which guidelines were developed, the different national scales for grading levels of evidence and strength of recommendation were initially converted to the Oxford Centre for Evidence Based Medicine (OCEBM) levels of evidence (LoE) and strength of recommendations (SoR) (Appendix 3-4).[21] However, the heterogeneity between grading systems made this difficult. Thus, we opted to focus only upon SoR and to convert them into three categories: highest, moderate, and lowest grade.

Antibiotic Stewardship

As we were unable to find a validated scoring system to assess if a clinical guideline includes antibiotic stewardship considerations. principles, we based our methodology on a study by Elias et al. examining antibiotic stewardship in guidelines.[22] We thus proposed six principles that demonstrate consideration of antibiotic stewardship, based upon the author’s consensus opinion. The principles are the inclusion in the guideline of 1) diagnostic criteria; 2) criteria for initiation of antibiotic therapy; 3) dosage; 4) route of administration of antibiotic therapy; 5) what percentage of antibiotic recommendations was based upon local resistance patterns (i.e. if 2 of 3 recommended antibiotics were supported by local epidemiological data, 67% was awarded) and 6) whether guidelines recommending amoxicillin or amoxicillin-clavulanic acid based the recommended dosage on local resistance data. Amoxicillin/amoxicillin-clavulanic acid was chosen because in contrast to other antibiotics, a higher dosage is recommended to overcome resistant strains.[23]

For peer review only

RESULTS

Overview of existing guidelines

The search retrieved 7340 records (Figure 1). Of these, 19 guidelines were obtained. National guidelines were obtained from 17 of 32 European countries (53%) (Figure 2). The majority of these were from Western Europe and Scandinavia. The remaining 15 countries did not have specific national guidelines on AOM in children. The intended audience of the guidelines obtained was mainly general practitioners and paediatricians, although some included nurses or physician's assistants (Appendix 5).

Diagnostic criteria

Fifteen of 17 (88%) European guidelines outlined the signs and symptoms for diagnosing AOM (Appendix 6) with considerable similarities between the guidelines. Twelve (71%) utilised strict combinations of three diagnostic criteria: 1) Acute onset of symptoms (i.e. otalgia, fever), 2) evidence of middle ear effusion (i.e. tympanic membrane (TM) bulging of tympanic membrane or otorrhea on examination and 3) Inflammation of TM on examination.

Otoscopy

Standard otoscopy was advised by 15 (88%) European guidelines (Appendix 7). Pneumatic otoscopy and tympanometry were also recommended by nine (60%) and seven guidelines (47%) respectively.

Additional investigations

No guidelines advised routine laboratory or radiographic investigations (Appendix 8). Nine of 17 (53%) guidelines stated specific indications for carrying out investigations. Eight of these advised consideration of a culture sample of the middle ear (ME) via tympanocentesis, most commonly for treatment failure (6/9; 67%) and complications such as mastoiditis (4/9; 44%). Three guidelines (3/9; 33%) discussed imaging modalities such as a CT brain when investigating secondary mastoiditis.

Approach to antibiotic administration

There were two approaches towards antibiotic administration: immediate antibiotic prescription and a watchful waiting approach. Fifteen of the European guidelines

For peer review only

(88%) recommended a watchful waiting approach (Table 1). Clinicians were encouraged to prescribe antibiotics if symptoms persisted for 2-3 days or if any clinical deterioration. Bilateral AOM was the most common indication for immediate antibiotic administration (13/15, 87%). (Table 2, Appendix 9). Two guidelines (Czech Republic and Finland) (12%) advocated for administration of immediate antibiotics for every diagnosed case of AOM, although the Finnish guideline included the option of watchful waiting if the child could be reviewed in 2-3 days.

Table 1: Strength of recommendations supporting immediate and watchful waiting approach to antibiotic administration in European, AAP, and WHO guidelines

Treatment approach	Strength of Recommendation
Immediate antibiotics	
WHO	Strong recommendation
Finland	A
Czech Republic	No grade
Watchful waiting approach	
France	A
Italy	A
Spain	A
Denmark	√
Poland	B
Portugal	IIa
UK	B
USA	Recommendation
Belgium	No grade
Germany	No grade
Ireland	No grade
Luxembourg	No grade
Netherlands	No grade
Norway	No grade
Sweden	No grade
Switzerland	No grade

Table 2: Indications for consideration of immediate antibiotic treatment in European and AAP guidelines

Guideline	Age (months)*	Carer input †	Bilateral AOM aged <24 months ‡	Severe symptoms §	Co-morbidities	Recurrent AOM	TM perforation/ Otorrhoea
Italy			+	+			+
Spain	<24		+	+		+	+
Denmark	<6		+	+			+
France	<24			+			
Portugal	<6		+	+		+	+
AAP		+	+	+			
Norway	<12		+				
Poland	<6	+	+	+	+		+
Belgium	<6		+	+	+		+
Finland	<24		+				+
Germany	<6		+	+	+	+	+
Ireland							+
Luxembourg	<24		+	+			
Netherlands	<6		+	+	+		+
Sweden	<12		+	+	+		+
Switzerland	<24		+	+	+		+

*Sweden: also children aged >12 years
† Poland: based on carer input if <24 months of age
‡ Belgium, Finland and Sweden: bilateral at any age; Luxembourg: after consultation with parents
§ Symptoms include fever, otalgia, pain, vomiting and diarrhea. Switzerland: only if <24 months old
NB: Finland- while treatment with antibiotics is the rule, Finland does give the option to watch and wait. In those children, it suggests immediate antibiotics if some criteria are met.

First line antibiotic therapy

Fourteen (82%) European guidelines recommended oral amoxicillin as an option for first line treatment (Figure 3). Seven (50%) recommended a high dose (75-90mg/kg/day), and five (70%) a low dose (30-60mg/kg/day). Stratification to high or low dose amoxicillin for children in the UK SIGN guideline is weight dependent; the Irish guidelines did not specify a dose. All the Nordic countries (ie Denmark, Sweden, and Norway) except Finland included Oral Penicillin V 24-75mg/kg/day as a first line choice (Appendix 10).

Treatment failure and penicillin allergy: Alternative antibiotic treatments

In case of treatment failure, per oral (PO) and intravenous (IV) amoxicillin-clavulanic acid (11/15, 73%) and IV/intramuscular (IM) ceftriaxone (8/15, 53%) were most

For peer review only - http://bmjopen.bmj.com/site/about/guidelines.xhtml

commonly recommended. In case of penicillin allergy, guidelines advised either PO clarithromycin (8/16; 50%) or PO trimethoprim-sulfamethoxazole (6/16; 38%) (Appendix 10).

Quality assessment: AGREE II scores

All guidelines were appraised using the AGREE Criteria (Table 3). In four of seven domains (i.e. 2, 3, 5, and 6), European guidelines obtained a mean score of <42%, while only two domains (i.e. 1 and 4) scored above 63% (Appendix 11-12).

Table 3: AGREE II scores (%) of European, AAP and WHO guidelines

Domain number	Domain name	European Mean (Range)	AAP Mean	WHO Mean
1	Scope and Purpose	57 (10-100)	97	94
2	Stakeholder involvement	41 (0-92)	67	58
3	Rigour of development	34 (0-83)	88	80
4	Clarity of presentation	78 (21-100)	89	92
5	Applicability	23 (0-58)	35	60
6	Editorial independence	29 (0-96)	54	83

SoR and LoE

Ten of 17 European guidelines (59%) based their certainty of evidence (ie Level of evidence (LoE) and Strength of Recommendations (SoR)) upon a variety of methodologies (Appendix 5) 4). The only crossover was between Poland and Spain, who utilised a methodology from the Infectious Diseases Society of America. SoR was often based upon study design (i.e. multiple RCTs) but for some was based on more subjective assessments (i.e. "well conducted studies"). Despite the relative homogeneity in recommendations between guidelines, most recommendations did not specify a SoR (Table 2, Figure 3). Of the ones that did, the highest number (8/10; 80%) was for indications for antibiotic administration.

Antibiotic stewardship

The majority of guidelines provided diagnostic criteria for AOM, specifications on when to start antibiotics, the route of administration and the duration of treatment (Table 4). However, less than half referred to local AMR patterns, only four (24%) also included

For peer review only

both local AMR data and specified resistance levels to amoxicillin/amoxicillin-clavulanic acid to guide local choices.

Table 4: Antibiotic stewardship and AOM guidelines

	Do guidelines provide diagnostic criteria?	Do guidelines specify when to initiate antibiotics?	Do guidelines specify route of administration?	Do guidelines specify duration of antibiotic regimens?	Are antibiotic recommendations based on local AMR patterns?		
					Percentage of antibiotic recommendations based on local local AMR patterns	Amoxicillin dosage based on l local AMR patterns	Amoxicillin-clavulanic acid dosage based on local AMR patterns
Belgium	Yes	Yes	Yes	Yes	80%	Yes	Yes
Czech Rep.	Yes	Yes	Yes	Yes	0%	Unclear	Unclear
Denmark	Yes	Yes	Yes	Yes	0%	Not applicable	Unclear
Finland	Yes	Yes	Yes	Yes	62.50%	Yes	Yes
France	Yes	Yes	Yes	Yes	0%	Unclear	Unclear
Germany	Yes	Yes	Yes	Yes	0%	Unclear	Unclear
Ireland	Unclear	Yes	Yes	Unclear	0%	Unclear	Not applicable
Italy	Yes	Yes	Yes	Yes	67%	Yes	Yes
Luxembourg	Yes	Yes	Yes	Yes	0%	Unclear	Unclear
Netherlands	Yes	Yes	Yes	Yes	100%	Yes	Yes
Norway	Yes	Yes	Yes	Yes	0%	Unclear	Not applicable
Poland	Yes	Yes	Yes	Yes	100%	Yes	Not applicable
Portugal	Yes	Yes	Yes	Yes	71%	Yes	Yes
Spain	Yes	Yes	Yes	Yes	100%	Yes	Yes
Sweden	Yes	Yes	Yes	Yes	100%	Unclear	Not applicable
Switzerland	Unclear	Yes	Yes	Yes	0%	Unclear	Unclear
UK	Yes	Yes	Yes	Yes	0%	Unclear	Unclear
USA	Yes	Yes	Yes	Yes	100%	Yes	Yes
WHO	Yes	Yes	Yes	Yes	Not applicable	Not applicable	Not applicable

DISCUSSION

Approximately half of the 32 EU/EFTA countries have AOM guidelines. Diagnosis of AOM was based upon similar signs and symptoms. The vast majority of European guidelines advocated for a watchful waiting approach to antibiotic therapy. Amoxicillin for five to ten days was the most commonly recommended first-line antibiotic, with a similar number of countries advising for high and low dosages. The most common indications were severe symptoms, and bilateral infection in infants <24 months of age. Tympanocentesis was commonly reserved for treatment failure. Our quality

assessment found low mean AGREE II scores of less that 45% in most domains. Just over half of the guidelines specified SoR to support treatment directives. Less than half of the guidelines referred to local patterns of AMR.

Strengths of our study include the comprehensiveness of our three-tiered search strategy to identify a large number of guidelines and the use of AGREE-II, an internationally recognised guideline appraisal tool. Our analysis included an assessment of SoR and whether antibiotic stewardship, a key measure to reduce AMR, was included. However, our results should be interpreted in light of some limitations. The OCEBM recommends guidelines to contain both LoE and SoR to support clinical recommendations. However, the heterogeneity of LoE across countries limited useful comparison. Thus, we decided to compare SoR, which is easier but provides weaker information about the quality of evidence.

Previously published works demonstrated a common consensus in criteria for AOM diagnosis, and that watchful waiting period was the standard of care in Europe; amoxicillin was also found to be the most commonly recommended antibiotic.[24-26] In comparison to these studies, our work aimed to compare multiple facets of AOM management in Europe, including grading their quality, comparison of SoR, and assessing their inclusion of local AMR data.[26] Zeng *et al* also used AGREE II scores to assess quality of upper respiratory tract infections guidelines including three AOM guidelines from Japan, USA, and the UK.[27] We note a >10-point discrepancy in scoring in two of six domains between Zeng and ourselves for UK SIGN and US AAP AOM guidelines. This may indicate inter-user variability in AGREE II scoring.[28 29] Elias et al. assessed global infectious diseases guidelines and found that local AMR patterns were taken into account in 50-75% of recommendations, which is in range of our finding of 53%.[22]

The development of clinical guidelines according to the high standards of the AGREEII criteria is a resource intensive exercise. Despite existing resources dedicated to guideline development, many guidelines received low AGREE II scores.[30 31] Duplicating this process across European countries to reach similar conclusions does not seem efficient. For processes that do not depend on local factors (such as identifying the scope and purpose of guidelines, literature search, and the appraisal of

For peer review only

the evidence), perhaps guideline developers could consider formulating general recommendations through centralised European organisations. This is already the case for other medical specialities, for example, the European Joint Task Force for cardiovascular disease prevention involves provides guidelines that can be used across Europe[32] However, antimicrobial stewardship requires a local adaptation of guidelines because the local AMR patterns are influenced by many local factors. For example, not all European countries have introduced multivalent pneumococcal conjugated vaccines (PCVs) into their routine immunisation schedule. This is likely to impact local epidemiology of resistant organisms through an increase in non-vaccine type strains.[33 34] Therefore, countries would need to ensure the final recommendations fits local antibiotic resistance patterns. The latter implies the implementation of robust epidemiological surveillance systems in each country, and linkage between guidelines developers and these surveillance systems. This approach would allow the development of guidelines of better quality and better adapted to local contexts, which might contribute to reducing the spread of AMR.

CONCLUSION

Review of guidelines reveals major similarities in AOM management across Europe. Existing European guidelines scored poorly in most AGREE II domains. Consideration of local epidemiology and resistance patterns appears to be limited. Centrally produced guidelines adapted for local care pathways, user and patient preferences, as well as for local antimicrobial resistance patterns may provide more targeted recommendations and reduce unnecessary antibiotic administration, and help reducing the spread of antibiotic resistance.

SUMMARY BOXES

Section 1: What is already known on this topic

- AOM is one of the most common infections in children and young people accounting for a large proportion of antibiotic prescription in children
- Clinical guidelines are important tools for improving patient care and antibiotic stewardship to reduce antibiotic resistance

Section 2: What this study adds

- There are significant similarities in choice, duration, and indications for antibiotic treatment between European AOM guidelines

For peer review only

- The majority of guidelines do not refer to local antibiotic resistance patterns
- Centrally developed and locally adapted guidelines may provide more targeted recommendations and reduce unnecessary antibiotic administration

ACKNOWLEDGEMENTS

We would like to acknowledge Professor Mike Levin and our colleagues from the PERFORM consortium and all our contacts below who helped us in obtaining national guidelines:

Daniela Klobassa (Austria)
Jan Verbakel (Belgium)
Žaneta Jelčić (Croatia)
Stephanie Menikou (Cyprus)
Linda Alderson (Czech Republic)
Ivan Peychl (Czech Republic)
Juri Lindy Pedersen (Denmark)
Rikke Jorgensen (Denmark)
Piia Jogi (Estonia)
Eda Tamm (Estonia)
Irja Lutsar (Estonia)
Inga Ivaskeviciene (Estonia)
Niina Valtanen (Finland)
Esa Korpi (Finland)
Jean Christophe Mercier (France)
Romain Basmaci (France)
Ulrich von Both (Germany)
Florian Gothe (Germany)
Johanna Krone (Germany)
Christoph Bidlingmanier (Germany)
Veronika Louizou (Greece)
Konstatinos Kakleas (Greece)
Maeve Kelleher (Ireland)
Silvia Bressan (Italy)
Dace Zavadska (Latvia)
Simona Sabulyte (Lithuania)
Armand Biver (Luxembourg)
Simon Attard-Montalto (Malta)
David Pace (Malta)
Arne Martin Slatsve (Norway)
Ingebjørg Fagerli (Norway)
Maria Pokorska-Śpiewak (Poland)
Kacper Toczylowski (Poland)
Magdalena Marczyńska (Poland)
Carolina Costa (Portugal)
Irina Branescu (Romania)

For peer review only

Diana Moldovan (Romania)
Keti Vincek (Slovenia)
Frederico Maritinon Torres (Spain)
Giannos Orfanos (Sweden)
Irina Rivero (Spain)
Pablo Obando Pacheco (Spain)
Philipp Agyeman (Sweden)
Hermione Lyall (UK)
Ana Turkova (UK)

ADDITIONAL REQUIREMENTS

Ethics Approval

Not applicable

Transparency Declaration

The lead author Hijiri G Suzuki* affirms that this manuscript is an honest, accurate, and transparent account of the study being reported; that no important aspects of the study have been omitted; and that any discrepancies from the study as planned (and, if relevant, registered) have been explained.

*The manuscript's guarantor.

Funding

HGS did not receive any funding. RGN was supported by NIHR Academic clinical fellowship and lectureship award programme. JED and SY are supported by PERFORM, a consortium funded by the European Union's Horizon 2020 programme, under grant agreement No. 668303. The funding sources did not take part in the design, analysis, interpretation of data, writing of the report or decision to submit the article for publication. All authors had full access to all the data, and can take responsibility for the integrity of the data and the accuracy of the data analysis.

Data Sharing Statement

The primary data for this study was treatment guidelines, these can be shared on request to the corresponding author

Competing Interests Declaration

For peer review only

No competing interests. All authors have completed the ICMJE uniform disclosure form at www.icmje.org/coi_disclosure.pdf and declare: no support from any organisation for the submitted work; no financial relationships with any organisations that might have an interest in the submitted work in the previous three years; no other relationships or activities that could appear to have influenced the submitted work.

Authorship Contributions:

All authors contributed to the conception of the study, study design, interpretation of the results, and contributed significantly to the drafting and revising of the final manuscript. All authors contacted experts in their scientific networks to obtain additional guidelines and check the use and validity of those identified. HGS was responsible for the systematic database search. HGS and JED was responsible for the AGREE II scoring. JED was responsible for the six principles of antibiotic stewardship in this manuscript. All authors have seen and agree with the final version of the manuscript.

References

1. Schilder AG, Chonmaitree T, Cripps AW, et al. Otitis media. *Nat Rev Dis Primers* 2016;2:16063. doi: 10.1038/nrdp.2016.63 pmid 27604644
2. Palma S, Rosafio C, Del Giovane C, et al. The impact of the Italian guidelines on antibiotic prescription practices for acute otitis media in a paediatric emergency setting. *Ital J Pediatr* 2015;41:37. doi:10.1186/s13052-015-0144-4 pmid 4428229
3. Kaur R, Morris M, Pichichero ME. Epidemiology of Acute Otitis Media in the Postpneumococcal Conjugate Vaccine Era. *Pediatrics* 2017;140(3) doi: 10.1542/peds.2017-0181 pmid28784702
4. Coker TR, Chan LS, Newberry SJ, et al. Diagnosis, microbial epidemiology, and antibiotic treatment of acute otitis media in children: a systematic review. *Jama* 2010;304:2161-69. doi: 10.1001/jama.2010.1651 pmid 21081729
5. Leibovitz E. Acute otitis media in pediatric medicine: current issues in epidemiology, diagnosis, and management. *Paediatr Drugs* 2003;5 Suppl 1:1-12. pmid 14632101
6. Thompson PL, Gilbert RE, Long PF, et al. Has UK guidance affected general practitioner antibiotic prescribing for otitis media in children? *J Public Health (Oxf)* 2008;30:479-86. doi: 10.1093/pubmed/fdn072 pmid 18765405
7. Chen YJ, Hsieh YC, Huang YC, et al. Clinical manifestations and microbiology of acute otitis media with spontaneous otorrhea in children. *J Microbiol Immunol Infect* 2013;46:382-8. doi: 10.1016/j.jmii.2013.04.001 pmid 23757372
8. van Dongen TM, Venekamp RP, Wensing AM, et al. Acute otorrhea in children with tympanostomy tubes: prevalence of bacteria and viruses in the post-pneumococcal conjugate vaccine era. *Pediatr Infect Dis J* 2015;34:355-60. doi: 10.1097/inf.0000000000000595 pmid 25764097
9. Tähtinen PA, Laine MK, Huovinen P, et al. A Placebo-Controlled Trial of Antimicrobial Treatment for Acute Otitis Media. *New England Journal of Medicine* 2011;364(2):116-26. doi: 10.1056/NEJMoa1007174

For peer review only

10. Hoberman A, Paradise JL, Rockette HE, et al. Treatment of Acute Otitis Media in Children under 2 Years of Age. *New England Journal of Medicine* 2011;364:105-15. doi: 10.1056/NEJMoa0912254 pmid 21226576
11. Rovers MM, Glasziou P, Appelman CL, et al. Antibiotics for acute otitis media: a meta-analysis with individual patient data. *Lancet* 2006;368:1429-35. doi: 10.1016/s0140-6736(06)69606-2 pmid 17055944
12. Groth A, Enoksson F, Hermansson A, et al. Acute mastoiditis in children in Sweden 1993-2007-no increase after new guidelines. *Int J Pediatr Otorhinolaryngol* 2011;75(12):1496-501. doi: 10.1016/j.ijporl.2011.08.015
13. Venekamp RP, Sanders S, Glasziou PP, et al. Antibiotics for acute otitis media in children. *Cochrane Database Syst Rev* 2013(1):Cd000219. doi: 10.1002/14651858.CD000219.pub3 pmid 21945244
14. Centres for Disease Control and Prevention. Antibiotic Resistance Threats in the United States. 2013. Available from: https://www.cdc.gov/drugresistance/pdf/ar-threats-2013-508.pdf

15. Costelloe C, Metcalfe C, Lovering A, et al. Effect of antibiotic prescribing in primary care on antimicrobial resistance in individual patients: systematic review and meta-analysis. *Bmj* 2010;340:c2096. doi: 10.1136/bmj.c2096 pmid 20483949

16. Sibbald AD. Acute otitis media in infants: the disease and the illness. Clinical distinctions for the new treatment paradigm. *Otolaryngol Head Neck Surg* 2012;147:606-10. doi: 10.1177/0194599812455780 pmid 26941560
17. Pouwels Koen B HS, Llewelyn Martin J, Walker Ann Sarah, McNulty Cliodna AM, Robotham Julie V et al. . Duration of antibiotic treatment for common infections in English primary care: cross sectional analysis and comparison with guidelines. *BMJ* 2019;364(364) doi: https://doi.org/10.1136/bmj.l440
18. WHO. Global action plan on antimicrobial resistance. 2015. Available from: http://www.wpro.who.int/entity/drug_resistance/resources/global_action_plan_eng.pdf
19. Laxminarayan R, Duse A, Wattal C, et al. Antibiotic resistance-the need for global solutions. *Lancet Infect Dis* 2013;13:1057-98. doi: 10.1016/s1473-3099(13)70318-9 pmid 24252483
20. The AGREE Next Steps Consortium. The AGREE II Instrument. 2009. Available from: https://www.agreetrust.org/wp-content/uploads/2013/10/AGREE-II-Users-Manual-and-23-item-Instrument_2009_UPDATE_2013.pdf
21. Jeremy Howick, Iain Chalmers, Paul Glasziou, Trish Greenhalgh, Carl Heneghan, Alessandro Liberati, Ivan Moschetti, Bob Phillips, and Hazel Thornton. "Explanation of the 2011 Oxford Centre for Evidence-Based Medicine (OCEBM) Levels of Evidence (Background Document)". Oxford Centre for Evidence-Based Medicine. https://www.cebm.net/index.aspx?o=5653
22. Elias C, Moja L, Mertz D, et al. Guideline recommendations and antimicrobial resistance: the need for a change. *BMJ Open* 2017;7:e016264. doi: 10.1136/bmjopen-2017-016264 pmid 28751488
23. Opatowski L, Mandel J, Varon E, et al. Antibiotic dose impact on resistance selection in the community: a mathematical model of beta-lactams and Streptococcus pneumoniae dynamics. *Antimicrob Agents Chemother* 2010;54:2330-37. doi: 10.1128/AAC.00331-09 pmid 20231396
24. Ovnat Tamir S, Shemesh S, Oron Y, Marom T. Acute otitis media guidelines in selected developed and developing countries: uniformity and diversity. *Arch Dis Child* 2017;102:450-57. doi: 10.1136/archdischild-2016-310729 pmid 27601361
25. Andre M AI, Hemlin C, Nasta F. Behandling av akut otitis media i andra länder - översikt över 28 nationella riktlinjer [English Title: Treatment of acute otitis media in other countries- a survey of 28 national guidelines]. *Information från Läkemedelsverket* 2010;21:37-43.
26. Toll EC, Nunez DA. Diagnosis and treatment of acute otitis media: review. *J Laryngol Otol* 2012;126:976-83. doi: 10.1017/s0022215112001326 pmid 22809689

For peer review only

27. Zeng L, Zhang L, Hu Z, et al. Systematic review of evidence-based guidelines on medication therapy for upper respiratory tract infection in children with AGREE instrument. *PLoS One* 2014;9:e87711. doi: 10.1371/journal.pone.0087711 pmid 24586287

28. Marciano NJ, Merlin TL, Bessen T, et al. To what extent are current guidelines for cutaneous melanoma follow up based on scientific evidence? *Int J Clin Pract* 2014;68:761-70. doi: 10.1111/ijcp.12393 pmid 24548269

29. Brouwers MC, Kho ME, Browman GP, et al. AGREE II: advancing guideline development, reporting and evaluation in health care. *CMAJ* 2010;182:E839-42. doi:10.1503/cmaj.090449 pmid 20603348

30. Lomotan EA, Michel G, Lin Z, et al. How “should” we write guideline recommendations? Interpretation of deontic terminology in clinical practice guidelines: survey of the health services community. *Qual Saf Health Care.* 2010;19:509-13. doi: 10.1136/qshc.2009.032565 pmid 20702437

31. Guyatt GH, Oxman AD, Schünemann HJ, et al. GRADE guidelines: a new series of articles in the Journal of Clinical Epidemiology. *J Clin Epidemiol* 2011;64:380-2. doi: 10.1016/j.jclinepi.2010.09.011 pmid 21185693

32. Hoes AW, Agewall S, Albus C et al. 2016 European Guidelines on cardiovascular disease prevention in clinical practice: The Sixth Joint Task Force of the European Society of Cardiology and Other Societies on Cardiovascular Disease Prevention in Clinical Practice (constituted by representatives of 10 societies and by invited experts): Developed with the special contribution of the European Association for Cardiovascular Prevention & Rehabilitation (EACPR). *Eur J Prev Cardiol* 2016;37: 2315-2381. doi: 10.1177/2047487316653709. pmid 27222591

33. European Centre for Disease Prevention and Control. Surveillance of antimicrobial resistance in Europe 2016: Annual Report of the European Antimicrobial Resistance Surveillance Network (EARS-Net). 2017. Available from: https://ecdc.europa.eu/sites/portal/files/media/en/publications/Publications/antimicrobial-resistance-europe-2015.pdf

34. Henriques-Normark B, Normark S. Bacterial vaccines and antibiotic resistance. *Ups J Med Sci* 2014;119:205-8. doi: 10.3109/03009734.2014.903324 pmid 24694025

35. Institut National D'Assurance Maladie-invalidité. L'usage rationnel des antibiotiques chez l'enfant en ambulatoire.[The rational usage of antibiotics in ambulating children]. 2016. Available https://www.inami.fgov.be/SiteCollectionDocuments/consensus_texte_court_20160602.pdf

36. Bébrová Elizabeth JB, Cizek H, Vaclav D et al._Doporučený postup pro antibiotickou léčbu komunitních respiračních infekcí v primární péči. [The recommended procedure for antibiotic treatment of community respiratory infections]

37. DSAM Luftvejsinfektioner - diagnose og behandling 2014 [Respiratory tract infections - diagnosis and treatment, the Danish Society of General Practitioners, 2014]. 2014. Available from http://vejledninger.dsam.dk/luftvejsinfektioner/

38. Heikkinen T, Jero J, Klockars T, et al. Välikorvatulehdus (lasten äkillinen) [Otitis media acute in children]. 2017. Available https://www.kaypahoito.fi/hoi31050

39. Agence française de sécurité sanitaire des produits de santé.(AFSSAPS) Recommandations de Bonne Pratique- Antibiothérapie par voie générale en pratique courante dans les infections respiratoires hautes [Recommendations for good practice- Antimicrobials by general route in current practice in upper respiratory tract infections]. 2011. Available http://www.infectiologie.com/UserFiles/File/medias/Recos/2011-infections-respir-hautes-recommandations.pdf

40. Deutsche Gesellschaft für Allegemeinmedizin und Familienmedizin (DEGAM). DEGAM-Leitlinie Nr 7-Ohrenschmerzen [Earache]. 2014. Available https://www.awmf.org/en/clinical-practice-guidelines/search-for-guidelines.html#result-list

41. Health Services Executive and Royal College of Physicians Ireland . Acute Otitis Media. 2012. Available from https://www.hse.ie/eng/services/publications/clinical-strategy-and-programmes/paediatrics-acute-otitis-media.pdf

For peer review only

42. Marchisio P, Bellussi L, Di Mauro G et al. Acute otitis media: From diagnosis to prevention. Summary of the Italian guideline. *Int J Pediatr Otorhinolaryngol* 2014;74:1209-16. doi 10.1016/j.ijporl.2010.08.016 pmid 20843561

43. Conseil Scientifique Domaine de la Santé. Otite moyenne aiguë. [Acute Otitis media]. 2017.

44. Damoiseaux R, Venekamp R, Eekhof J, Bennebroek Gravenhorst F et al. NHG- Standaard Otitis media acuta bij kinderen Otitis media acuta bij kinderen [NHG Standard Acute Otitis media in children]. 2014. Available from https://www.nhg.org/standaarden/volledig/nhg-standaard-otitis-media-acuta-bij-kinderen.

45 Antibiotikasenteret for primærmedisin. Antibiotikabruk i primærhelsetjenesten- akutt otitis media [Acute otitis media]. 2016. Available from http://www.antibiotikaiallmennpraksis.no/index.php?action=showtopic&topic=VMpmsqDE\

46. Hryniewicz W, Piotr AP, Radzikowski, A, Hassmann-Poznańska, E et al.Rekomendacje postępowania w pozaszpitalnych zakażeniach układu oddechowego.[Recommendations for the treatment of oupatient respiratory infections] 2016. Available from http://www.antybiotyki.edu.pl/szpitalna-polit-rekomendacje-diagnost.php

47. Departamento da Qualidade na Saúde. Diagnóstico e tratamento da Otite Média Aguda na Idade Pediátrica. [Diagnosis and Treatement of Acute Otitis Media in Paediatrics]. 2014.

48. del Castillo Martína F BAF, de la Calle Cabrerab T, López Roblesc MV, et al. Asociación Española de Pediatría- Documento de consenso sobre etiología, diagnóstico y tratamiento de la otitis media aguda. *An Pediatr* 2012;77:8. doi: http://dx.doi.org/10.1016/j.anpedi.2012.05.026

49. *Läkemedelsverket*. Diagnostik, behandling och uppföljning av akut mediaotit (AOM) – ny rekommendation. [Diagnosis, treatment and follow up of acute otitis media- new recommendations].*Information från Läkemedelsverket* 2010;21:10-23.

50. Pediatric Infectious Disease Group of Switzerland. Recommendations pour le diagnostic et le traitement de Otite moyenne aiguë, Sinusite aiguë, Pneumonie (community-acquired), et Pharyngo-amygdalite chez l'enfant. [Recommendations for the diagnosis and treatment of acute otitis media, acute sinusitis, pneumonia (community acquired), and pharyngitis. 2010. Available http://www.pigs.ch/pigs/frames/documentsframe.html

51. Scottish Intercollegiate Guidelines Network. Diagnosis and management of childhood otitis media in primary care: A national clinical guideline. 2003.

Legends:

Figure 2

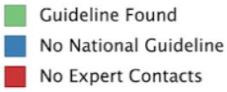

Figure 3

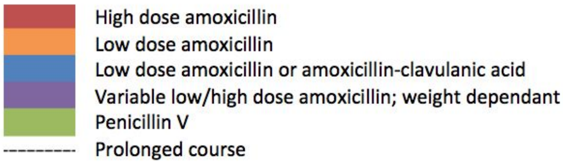

For peer review only

Table 1 and 2

Key
Highest grade
Moderate grade
Lowest grade
No grade

Table 4

Promotes antibiotic stewardship

Partially promotes antibiotic stewardship

Does not promote antibiotic stewardship

For peer review only

Figure 1: PRISMA Flow Diagram

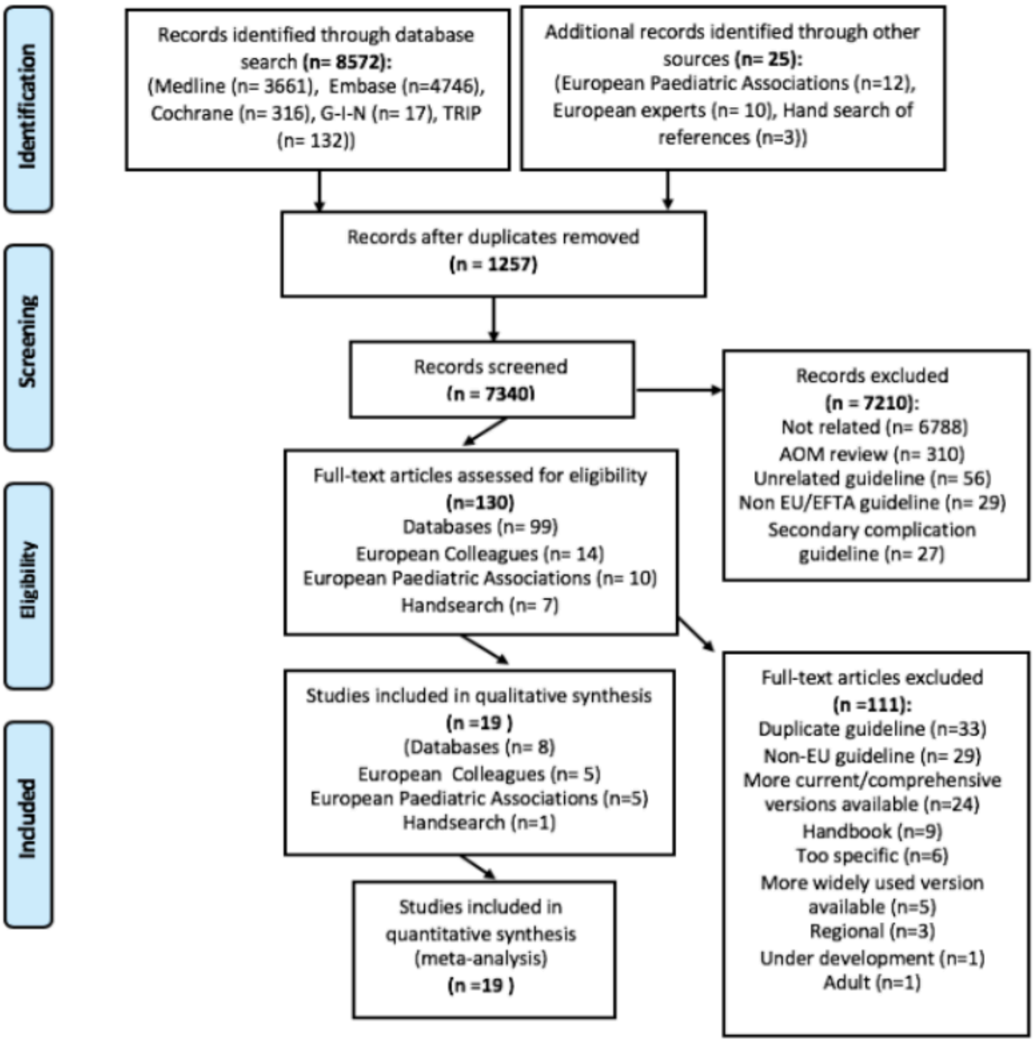

Figure 2: European AOM Guidelines (Lead group and year published)

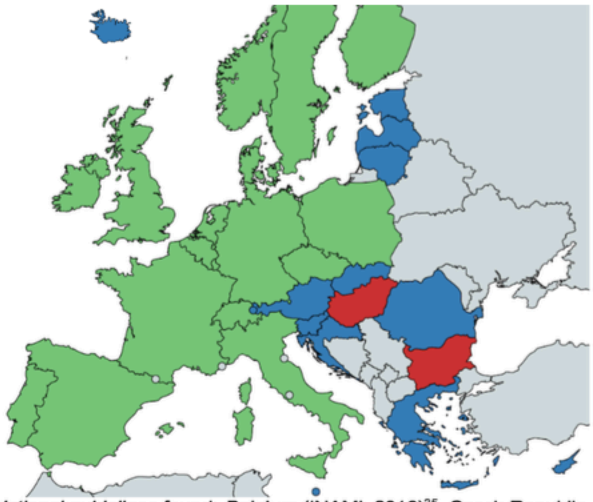

National guidelines found: Belgium (INAMI, 2016)[35], Czech Republic (CzMA, date unspecified)[36], Denmark (DSAM 2014)[37], Finland (Duodecim 2017)[38], France (AFSSAPS 2011)[39], Germany (DEGAM 2014)[40], Ireland (HSE 2012)[41], Italy (SIP 2010)[42], Luxembourg (CSDS 2007)[43], Netherlands (NHG 2014)[44], Norway (ASP 2016)[45], Poland (NIL 2016)[46], Portugal (DGS 2014)[47], Spain (AEPED 2012)[48], Sweden (MPA 2010)[49], Switzerland (PIGS 2010)[50], United Kingdom (SIGN 2003)[51]

er review only

Figure 3: Routine first line antibiotics: Initiation, choice, duration, and SoR

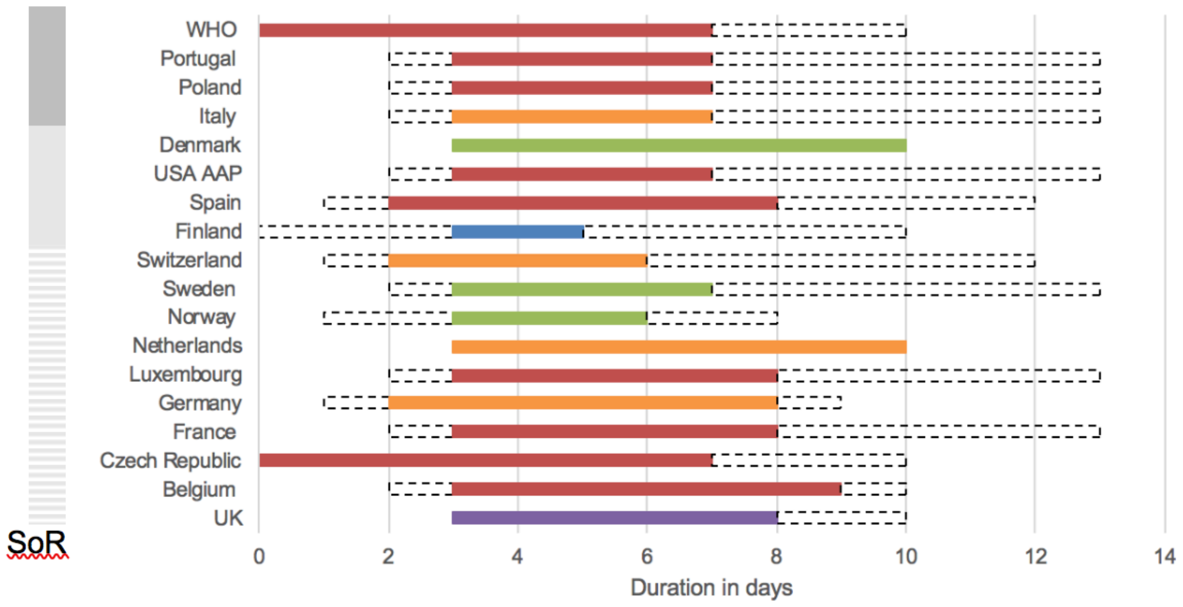

For peer review only

Protocol:

Clinical practice guidelines for acute otitis media in children: A systematic review and appraisal of European national guidelines

Project lead:	Shunmay Yeung
Research team members:	Ruud Nijman, Manuel Dewez, and Hijiri Suzuki

For peer review only

Clinical practice guidelines for acute otitis media in children: A systematic review and appraisal of European national guidelines

1. BACKGROUND

Management of febrile children can be challenging, and diagnostic and treatment practices vary across Europe. Guidelines are increasingly used to direct paediatric clinical practice towards a standardised level of care.

There have been several studies that have examined differences between guidelines from different countries. However, few studies exist that have systematically compared clinical guidelines across Europe. Such a comparison would provide important information regarding the current standard of care and the quality of guidelines used. This is particularly relevant in light of the increasing recognition that guidelines play an important role in antibiotic stewardship.

Acute otitis media (AOM) is an common cause of fever and frequent reason for consultation and antibiotic prescription in children.

2. AIMS:

To review guidelines for the management of AOM in children in Europe in order to compare the current approaches to diagnosis and clinical care with a focus on recommendations with regards antibiotics.

3. OBJECTIVES :

i. To collate national guidelines for management of AOM from European countries in the European Union (EU) or European Free Trade Association (EFTA)
ii. To collate national guidelines for management of AOM from the United States (US) and the World Health Organisation (WHO) as an external point of comparison
iii. To assess the quality of the guidelines
iv. To compare and describe key differences and similarities between national guidelines:
    - What are the differences, if any, in what informs how rapidly a child with AOM should be assessed (i.e. **triage process**)?
    - What are the differences, if any, in aspects of history, examination, and investigations (particularly in terms of using point of care tests) that inform the **diagnosis** of AOM in Europe? **What is the strength of evidence that informs the diagnosis?**
    - What are the differences, if any, in aspects of history, examination, diagnosis, and investigations (particularly in terms of using point of care tests) that inform the antibiotic **treatment** of AOM in Europe? **What is the strength of evidence that informs treatment decisions?**
    - What are the differences, if any, in aspects of history, examination, diagnosis and investigations (particularly in terms of using) that

For peer review only

inform the referral/ **admission criteria** for a child with AOM in Europe?

4. SEARCH METHODS

i. Electronic Databases

A literature search will be undertaken using Medline via Ovid, Embase, Cochrane Library, SIGN, G-I-N, BMJ Clinical Evidence and TRIP. Search terms will be decided upon by two reviewers, and performed by one reviewer. Search terms will be available in the Appendix of the review.

ii. Websites

The websites of national paediatric associations will be searched individually to find protocols. National associations will be found via international paediatric associations including the European Paediatric Association/Union of Union of National European Paediatric Societies and Associations (EPA/UNEPSA), and the European Academy of Paediatrics (EAP).

iii. Expert research network consultations

A network of research partners will be contacted to verify whether the identified guidelines are currently utilised, as well as to provide guidelines not obtained by the above procedures. All documents in English, Spanish, French, and Dutch will be read and translated into English by the reviewers. Other documents will be translated into English by electronic means. Translations will be verified by research partners fluent in that language.

iv. Reference searches

Bibliographies of papers and guidelines will be hand searched to identify further resources.

5. DATA SELECTION

i. Inclusion criteria (all criteria need to be met)

- National guideline or a guideline endorsed by a national society (i.e. Royal College of Paediatric and Child Health)
- Guideline must be published since 2000.
- Guideline from the EU, EFTA, USA, or the WHO.
- Guidelines for febrile children, using the national definition of childhood
- Guidelines either based upon a symptom (i.e. “Sore ear”) or upon a diagnosis (i.e. “acute otitis media”).
- A guideline that does not provide an overview of management, but only a specific aspect (i.e. “Antibiotic choices for children with pneumonia”) can be included **only** if no other national guideline that provides more comprehensive information is available

Importantly, inclusion criteria does not include a minimum result from the quality assessment, as one objective of the review is to assess the quality of all identified guidelines.

For peer review only

ii. Exclusion criteria

- Local hospital guidelines, either of a EU or non-EU country
- Guidelines that do not clearly differentiate between management of children and adults, whereby data specific to children cannot be extracted (i.e. only differentiate between adult and paediatric antibiotic dosage)
- Guidelines specific to a population of children with underlying syndromes, complex pathology, or significant co-morbidities (i.e. “Management of AOM in children with Down’s syndrome”).

6. METHODS OF REVIEW

Details of methods

Two reviewers will screen guidelines independently using a three-stage approach to reviewing the title, abstract, and full text. Reviewers will then confer to decide which meet inclusion criteria. Should reviewers disagree, the final decision will be made by the Project Lead.

Quality Assessment

i. Assessment of methodological quality

Methodological quality of each guideline will be assessed according to the AGREE II criteria, a standardized appraisal method that grades guidelines in multiple domains. Two reviewers will independently grade guidelines, and the mean of their results will be used as a final result as per AGREE II methodology.

ii. Assessment of levels of evidence

The second method will be to convert the level of evidence provided by the authors of each guideline to support strength of recommendations for each national guideline into the Oxford Centre for Evidence Based Medicine (CEBM) Levels of Evidence. One reviewer will be responsible for this, and results will be independently reviewed by a second reviewer.

iii. Antibiotic Stewardship

Consideration of principles of antibiotic stewardship should receive a score of Yes, No, or maybe based upon the following

- Provision of diagnostic criteria
- Initiation of antibiotics
- Route of antibiotic administration
- Duration of antibiotic administration
- Are guidelines based upon local antimicrobial resistance patterns?

7. DATA EXTRACTION

Data from guidelines will be independently extracted by two reviewers via a standardised template.

For peer review only

For peer review only

Appendix

Table of Contents

1) Search terms
   a) Medline via Ovid
   b) Embase via Ovid
   c) Cochrane Library
   d) Guidelines International Network (G-I-N)
   e) TRIP medical database
2) European Paediatric Association (EPA) Search
3) AOM protocols in Europe
4) National guidelines' level of evidence converted to Oxford Centre for Evidence Based Medicine (OCEBM)
5) National guidelines' Strength of recommendation (SoR) converted to OCEBM SoR
6) Locally defined Strength of Recommendation (SoR) for diagnostic criteria for AOM in Europe and the USA
7) Examination tools as recommended by European national guidelines
8) What investigations are advised in European, American, and WHO guidelines for AOM management in children
9) Indications for consideration of immediate antibiotic treatment
10) What antibiotic treatment is recommended?
11) AGREE scores by country (%)
12) AGREE scores by marker
   a) HS AGREE scores
   b) JED AGREE Scores
13) Acknowledgements

For peer review only

1) Search terms

a) Medline via Ovid

1	exp Otitis Media/
2	otitis media.tw.
3	acute otitis media.mp.
4	exp Respiratory Tract Infections/
5	aom.mp.
6	middle ear infect*.mp.
7	guideline.mp. or exp GUIDELINE/ or exp PRACTICE GUIDELINE/
8	guide.mp.
9	manage*.mp.
10	exp Clinical Protocols/
11	1 or 2 or 3 or 4 or 5 or 6
12	7 or 8 or 9 or 10
13	11 and 12
14	limit 13 to (yr="2007 - 2017" and "all child (0 to 18 years)")

For peer review only

b) Embase via Ovid

1	1. exp otitis media/
2	otitis media.tw.
3	acute otitis media/
4	exp respiratory tract infection/
5	aom.mp.
6	middle ear infect*.mp
7	exp practice guideline/
8	guide.mp.
9	manage*.mp.
10	exp clinical protocol/
11	1 or 2 or 3 or 4 or 5 or 6
12	7 or 8 or 9 or 10
13	13. 11 and 12
14	14. limit 13 to (yr="2007 - 2017" and child)

c) Cochrane library

	Search term/strategy
1	Exp otitis media
2	Otitis media
3	Glue ear
4	midd ear adj5 (infect* or inflame*)
5	Ome or aom
6	Guideline or practice guideline
7	Management of manage*

For peer review only

8	Clinical protocol
9	#1 or #2 or #3 or #4 or #5
10	#6 or #7 or #8
11	#9 or #10

Results then limited to 16/11/2007-16/11/2017

d) Guidelines International Network (G-I-N)

	Search term/strategy
1	Otitis and
2	Acute Otis media

e) TRIP Medical Database

	Search term/strategy
1	Acute otitis media and
2	Otitis
3	Limit Guidelines

2) European Paediatric association (EPA) Hand search:

http://www.epa-unepsa.org/?q=page/epa-unepsa-full-members

For peer review only

3) AOM protocols in Europe

Leading author organisations (Local language)	Leading author organisations (English translation)	Country/ Region	Year published/ updated	Aimed audience	Patient age group	What system used for LoE/SoR?
Institut national d'ass urance maladie-invalidité Comité d'évaluation des pratiques médicales en matière de medicaments (INAMI)	National Institute for Health and Disability Insurance Committee for the Medical Practice on Medicinal Products Evaluation	Belgium	2016	-	Children 0-15 years	Institutional
Odbornáspolečnost praktických dětských lékařů ČLS JEP, Společnost všeobecného lékařství ČLS JEP	CzMA Society of General Practice Society	Czech Republic	-	-	Children and Adults	None
Dansk Selskab for Almen Medicin (DSAM)	Danish Society for General Practice	Denmark	2014	General Practitioners, Parents and carers	Children 0-5 years	GRADE and Institutional
Suomalainen Lääkäriseura Duodecim	Finnish Medical Society Duodecim	Finland	2017	Primary health care	Children under school age	Institutional
Agence Française de Sécurité Sanitaire des Produits de Santé (AFSSAPS); Agence Nationale de Sécurité du Médicament et des Produits de Santé (ANSM)	French Agency for the Safety of Medicines and Health Products (AFSSAPS) (currently known as National Agency for the Safety of Medicines and Health Products (ANSM)	France	2011	-	Children >3 months of age	National Agency for Accreditation and Evaluation in Health (ANAES)

For peer review only

AWMF (Arbeitsgemeinschaft der wissenschaltlichen medizinischen Fachgesellschaften). Lead society: Deutsche Gesellschaft für Allgemeinmedizin und Familienmedizin (DEGAM)	Association of the Scientific Medical Societies in Germany. Lead society: Society of General Medicine and Family Medicine	Germany	2014	General practitioners, paediatricians, ENT surgeons, junior doctors, audiologists, and primary care workers.	Children and adult (outpatients)	Institutional (AWMF Consensus process S2k)
Health Service Executive and Royal College of Physicians Ireland	-	Ireland	2012	-	Children	None
Società Italiana di Pediatria (SIP) and Società Italiana di Otorinolaringologia Pediatrica	Italian Society of Paediatrics (SIP) and Italian Society of Pediatric Otolaryngology (SIOP)	Italy	2010	Paediatricians, ENT surgeons, general practitioners, nurses, physicians assistants	Children aged 2 months -18 years	Manual for Writing Clinical Practice Guidelines of the Programma Nazionale Linee Guida (PNLG)
Secrétariat du Conseil Scientifique - Domaine de la Santé	Scientific Council of the Ministry of Health	Luxembourg	2007	-	Children >3months of age	None
Nederlands Huisartsen Genootschap (NHG)	Dutch College of General Practitioners	Netherlands	2014	-	Children and adolescents up to 18 years of age	None

For peer review only

Antibiotikasenteret for primærmedisin (ASP)	National Antibiotics Centre for Primary Care	Norway	2016	Physicians, GPs, dentists, private practitioners, medical students	Children and adults	None
Narodowy Instytut Leków (NIL)	National Medicine Institute	Poland	2016	All specialities, including GPs, paediatricians, physicians, Respiratory physicians, ENT surgeons	Children and adults	Infectious Disease Society of America
Departamento da Qualidade na Saúde (DGS)	Department of Health Quality	Portugal	2014	Physicians of the health system	Children	European Society of Cardiology
Asociación Española de Pediatría (AEPED)	Spanish Association of Paediatrics	Spain	2012	-	Children	Infectious Disease Society of America
Läkemedelsverket	Swedish Medical Products Agency	Sweden	2010	-	Children and adults	None
Paediatric Infectious Diseases Group of Switzerland (PIGS)	-	Switzerland	2010	-	Children	None

For peer review only

Scottish Intercollegiate Guidelines Network (SIGN)	-	UK	2003 **Has been retracted by institution**	"All people working with children" including general practitioners (GPs), practice nurses, audiologists, paediatricians, otolaryngologists, audiological physicians, health visitors, social workers, public health physicians, users of services and all other professions caring for children.	-	Institutional
American Association of Paediatrics (AAP)	-	USA	2013	Paediatricians, GPs, Emergency specialists, ENT surgeons, Nurse Practitioners, Physician's assistants	6 months- 12 years	Institutional
World Health Organisation (WHO)	-	N/A	2013	Doctors, senior nurses, senior health workers	Sick young children in low-resource setting	Institutional

4) National guidelines' Levels of Evidence (LoE) converted to Oxford Centre for Evidence Based Medicine (OCEBM) LoE

For peer review only

**Oxford Centre for EBM Levels of Evidence**		**Belgium**	**Denmark**	**France**	**Finland**	**Italy**	**Norway**	**Poland and Spain**	**Portugal**	**UK SIGN**	**AAP**	**WHO**
1a	SR of RCTs	A/B/C	1a	1	A	I	1a	I	A	1++	A	High
1b	Individual RCT	A/B/C	1b	1	B	I/ II	1b	-	B	1++/ 1+/1-	-	-
1c	All or none	-	1c	-	-	-	-	-	-	-	X	-
2a	SR with homogeneity of cohort studies	-	2a	2	A	III	2a	II	A	2++	B/ C	High-moderate
2b	Individual cohort study	-	2b	-	B/ C	III	2a	-	B	2++/ 2+/2-	-	-
2c	Outcomes research; ecological studies	-	2c	-	C	-	-	-	-	2++/ 2+/2-	-	-
3a	SR with homogeneity of case-control studies	-	3a	3	A	IV	1b, 2a	II	A	2++/2+/2-	B/C	High-moderate
3b	Individual case-control study	-	3b	-	B/C	IV	1b, 2a	-	B	2++/2+/2-	-	-
4	Case series (and poor quality cohort and case-control studies	-	4	4	C	V	3	III	C	3	C/D	Low- very low

For peer review only

5	Expert opinion	-	5	-	D	VI	4	III	C	4	D	-
X	LOE that does not match Oxford	-	DS	-	-	-	2b	-	-	-	-	-

For peer review only

5) National guidelines' Strength of recommendation (SoR) converted to OCEBM SoR

Oxford Centre for EBM Strength of Recommendations		Belgium	Denmark	France	Finland	Italy	Norway	Poland and Spain	Portugal	SIGN	AAP	WHO
A	Consistent level 1 studies	-	A	A	A	A/ E	A	A/E	A	A	Strong recommendation/ Option	Strong
B	Consistent level 2 or 3 studies *or* extrapolations from level 1 studies	-	B	B	A/B	-	B	-	B	B	Strong recommendation/ Recommendation/ Option	Conditional /Weak
C	Level 4 studies *or* extrapolations from level 2 or 3 studies	-	C	C	C	-	C	-	C	C	Recommendation/Option	Conditional /Weak
D	Level 5 evidence *or* troublingly inconsistent or inconclusive studies of any level	-	D/ √	C	D	-	D	-	D	D	Option/ No recommendation	No Recommendation

X	*SOR that does not match Oxford CEBM*	1/2	DS	-		B/ C/ D		B/ C/ D	-	-		

6) Locally defined Strength of Recommendation (SoR) for diagnostic criteria for AOM in Europe and the USA

Guideline	Diagnostic criteria included?	National SoR
Finland	Yes	A
Italy	Yes	A
Poland	Yes	A
Portugal	Yes	I
USA	Yes	Recommendation
Belgium	Yes	No grade
Czech Republic	Yes	No grade
Denmark	Yes	No grade
France	Yes	No grade
Germany	Yes	No grade
Ireland	No	No grade
Luxembourg	Yes	No grade
Netherlands	Yes	No grade
Norway	Yes	No grade
Spain	Yes	No grade

Sweden	Yes	No grade
Switzerland	No	No grade
United Kingdom	Yes	No grade
WHO	Yes	No grade

For peer review only

7) Examination tools as recommended by European, American and WHO guidelines

Country	Otoscope	Pneumatic otoscope	Tympanometry	Other	National Level of evidence/ strength of recommendation	OCEBM level of evidence/strength of recommendation
Czech Republic	+				-	-
Denmark	+	+	+		-	-
Finland	+	+	+		-/B	-/B
France	+				-	-
Germany	+	+	+		-	-
Ireland	+				-	-
Italy	+	+	+	Mirror	II/ B<br>II/ A	1b/ X<br>Ib/ A
Luxembourg	+	+		Mirror<br>Ear,<br>Endoscope,<br>Operating microscope	-	-

Netherlands	+				-	-
Norway	+	+			-	-
Poland	+	+	+		A/ II	A/ 2a-2c
Portugal	+				-	-
Spain	+	+			-	-
Sweden	+	+	+		-	-
SIGN	+	+	+		2+/ -	2b-3b/ -
AAP	+	+	+		B/ Recommendatio n	2a-3a/B-C
WHO	+				-	-

No examination tools specified in Belgian and Swiss guidelines

For peer review only

8) What investigations are advised in European, American, and WHO guidelines for AOM management in children

**Country**	**Investigation**	**Circumstances**	*National LOE / SOR*	*Oxford LOE /SOR*

Czech Republic	• Culture via tympanocentesis or swab<br>• CRP	• Children <2 years of age	-	-
Finland	• Culture	• Complications of AOM<br>• Underlying immunodeficiency or significant co-morbidity<br>• Unwell patient requiring hospitalization<br>• To relieve extreme pain	-	-
France	• Culture via tympanocentesis	• Treatment failure	-	-
Germany	• Blood tests (FBC, CRP, Acute phase protein i.e. interleukin 6/BSG)<br>• Culture via tympanocentesis<br>• Ultrasound or xray	• Complications<br>• Recurrent AOM	-	-
Italy	• Blood tests (CRP, ESR, WCC)<br>• Culture via tympanocentesis<br>• Nasopharyngeal aspirate (only quantitatively)<br>• CT scan	For blood tests:<br>• For follow up<br><br>For tympanocetesis:<br>• Complications<br>• Immunocompromised<br>• Neonate<br>• Spontaneous otorrhea<br>• Sepsis<br>• Treatment failure<br>• Research purposes	Typanocentesis: V/ B<br>NPA: IV/B<br>CT scan: IV/A	Tympanocentesis: 4 / X<br>NPA: 3a-3b/X<br>CT scan: 3a-3b/ A

For peer review only

Luxembourg	• Culture via Tympanocentesis	• Aged <3 months<br>• Severe pain and convex ear on otoscopy<br>• Treatment failure	-	-
Norway	• Culture via tympanocentesis or swab of otorrhoea	• Treatment failure	-	-
Spain	• Blood tests (FBC, CRP, blood culture)<br>• Culture (Via tympanocentesis or swab of otorrhea)<br>• Lumbar puncture<br>• Skull/Temporal bone CT	• Complications<br>• Recurrent AOM<br>• Spontaneous otorrhea<br>• Treatment failure	-	-
Sweden	• Culture via tympanocentesis<br>• Nasopharyngeal swab	• Treatment failure	-	-
AAP	• Culture via tympanocentesis<br>• Nasopharyngeal aspirate	• Treatment failure	-	-

No Investigations advised: Belgium, Denmark, Ireland, Netherlands, Poland, Portugal, Switzerland, UK SIGN, WHO

For peer review only

For peer review only

9) **Indications for consideration of immediate antibiotic treatment**

Guideline	Age < 6 months	Age < 12 months	Age <24 months *	Co-morbidities	Recurrent AOM	Carer input †	Family history	Bilateral AOM aged <24 months‡	Severe symptoms §	TM perforation/ Otorrhoea	National SoR	
Italy								+	+	+	A	
Spain			+		+		+	+	+	+	A	-
Denmark	+							+	+	+	A	√
France			+						+		A	B
Portugal	+				+			+	+	+	Ia	IIa
AAP						+		+	+		SR	R
Norway		+						+			B	
Poland	+			+		+		+	+	+	Grade B	
Belgium	+			+				+	+	+	−	
Finland			+					+		+	−	
Germany			+	+	+			+	+	+	−	
Ireland										+	−	
Luxembourg			+					+	+		−	
Netherlands	+			+				+	+	+	−	
Sweden		+		+				+	+	+	−	
Switzerland			+	+				+	+	+	−	

*Sweden: also children aged >12 years

For peer review only

† Poland: based on carer input if <24 months of age
‡ Belgium, Finland and Sweden: bilateral at any age; Luxembourg: after consultation with parents
§ Symptoms include fever, otalgia, pain, vomiting and diarrhoea . Switzerland: only if <24 months old
NB: Finland- while treatment with antibiotics is the rule, Finland does give the option to watch and wait. In those children, it suggests immediate antibiotics if some criteria are met. UK SIGN- no indications outlined

For peer review only

10) What antibiotic treatment is recommended?

Guideline	First line*	Duration of first line therapy	Second line/Treatment failure	Third line/ Allergy to first line	National LOE / SOR	Oxford LOE /SOR
Belgium	PO amoxicillin 75mg-100/kg/day in 3 divided doses	7 days	Treatment failure :<br><br>cefuroxime axetil 30-50 mg / kg in 3 doses<br><br>amoxicillin-clavulanic acid 50 / 37.5 mg / kg in 3 doses	In case of Allergy to cephalosporins , either:<br>Co-trimoxazole (Trimethoprim 8 mg/kg/day and Sulfumethoxazole 40 mg/kg/day) in 3 divided doses<br><br>Levofloxacin 10 mg/ kg/day in 2 divided doses	For treatment choice: Expert Opinion/Weak recommendation<br><br>Duration: GRADE A - B, low recommendation)	For treatment choice: 5 /X<br><br>For duration: 1a-1b/ X
Czech Republic	PO amoxicillin 75-90mg/kg/day in 3 divided doses	7-10 days	Discusses targeted therapy to specific bacterial agents, but no empiric antibiotic for treatment failure	Allergy to beta lactams: co-trimoxazole	None	None
Denmark	PO Penicillin V 60mg/kg/day in 3 divided doses	7 days	Treatment failure<br><br><2 years of age:<br>Amoxicillin-clavulanic acid 10/2.5mg/kg/dose 8 hourly for 7 days	Penicillin allergy:<br>clarithromycin 7.5mg.kg/dose x2 for 7 days	Level 1a-1c and 5 / Grade A and √<br><br>For duration: No LOE/SOR	Level 1a-1c and 5

			2-12 years of age:<br>amoxicillin – clavulanic acid 1-/2.5mg/kg/dose 8 hourly for 7 days			
Finland	PO Amoxicillin 40mg/kg/day 8-12 hourly<br>OR<br>PO amoxicillin-clavulanic acid 40/5.7mg/kg/day in 2-3 divided doses	5 -7 days	If vomiting: IM Ceftriaxone (one dose)	Penicillin allergy:<br>cefaclor, cefuroximexetil, sulfa trimethoprim, azithromycin or clarithromycin	First line: C / C<br><br>(None for treatment duration)	1b, 2b-2c, and 3b-4
France	PO amoxicillin 80-90mg/kg/day in 2-3 divided doses	5 day duration if >2 years of age, 8-10 days if <2 years of age	Treatment failure:<br>PO Amoxicillin/clavulanic acid 80mg/kg/day) AND PO amoxicillin 70mg/kg/day)<br>OR<br>IM/IV ceftriaxone 50mg/kg daily for 3 days<br><br>PO Amoxicillin/clavulanic acid = otitis conjunctivitis syndrome	Allergy to beta lactams erythromycin-sulfafuraole or cotrimoxazole<br><br>Allergy to penicillins without allergy to cephalosporins: cefpodoxime	Only duration has LOE:<br><br>Level 1 and Professional Agreement/ Grade A<br><br>Duration for child <2 years of age is Level 1/ Grade A<br>>2 years of age is professional agreement (No LOE)<br><br>3rd line treatment: Professional	Level 1a

For peer review only

					agreement (No LOE)	
Germany	PO Amoxicillin 50mg/kg/day in 2-3 divided doses<br><br>If from country with high rates of penicillin resistance: PO amoxicillin 80-90mg/kg/day	7 days	Treatment failure: PO amoxicillin 80-90mg/kg/day<br><br>Second choice: PO cephalosporin including cefuroxime axetil (20-30mg/kg/day for 5 days),	Allergy to penicillins/cephalosporins: macrolide ie erythromycin 7 days	None	None
Ireland	Amoxicillin (no duration, no route, no frequency)	None	None	None	None	None
Italy	Mild symptoms and no otorrhea nor risk factors† :<br>PO Amoxicillin 50mg/kg/day in 2-3 divided doses<br><br>Severe symptoms, otorrhea, or risk factors for bacterial resistance<br>Amoxicillin-clavulanic acid 80-90mg/kg/day in 2-3 divided doses	Duration:<br><br>10 days <2 years of age<br>5 days >2 years of age	Treatment failure:<br>If they were treated with amoxicillin or cefaclor: amoxicillin plus clavulanic acid or cefpodoxime proxetil or cefuroxime axetil.<br><br>If they were being treated with a broad-spectrum antibiotic: intramuscular or intravenous ceftriaxone 50mg/kg once daily<br><br>Duration:	Penicillin allergy: macrolide	First line (for both) : Level I/ Grade A<br><br>Treatment failure: Level II/ Grade B<br><br>Duration Level I/ Grade B<br><br>Allergy: VI/D	First line: Level1a- 1b/ A<br><br>Treatment failure: Level 1b/ X<br><br>Duration: Level 1a-1b /X

For peer review only

			10 days <2 years of age or spontaneous otorrhea<br>5 days >2 years of age<br>3 days for ceftriaxone			
Luxembourg	Amoxycillin 80-90mg /kg/day in 3 divided doses<br><br>Alternatively if 'very severe cases'<br><br>Amoxicillin-clavulanic acid 80-90mg/kg/day in 3 divided doses	<6 years of age 10 days treatment<br><br>>6 years 5-7 days treatment	Treatment failure:<br><br>Amoxycillin/ clavulanate<br><br>Otherwise<br><br>cefuroxime axetil no dose, no frequency) or<br><br>ceftriaxone 50mg/kg/day for 3 days<br><br>or<br><br>azithromycin or clarithromycin or clindamycin	If vomiting: Ceftriaxone 50 mg / kg once daily for 3 days<br><br>Penicillin allergy: Cefuroxime 30mg/kg in two divided doses<br><br>Allergy to penicillin + cephalosporin: Azithromycin 10mg/kg/day for 6 days or clarithromycin 15mg/kg/day in two divided doses<br><br>Otherwise: sulfamethoxazole-trimethoprim 6019ng.kg of trimethoprim per day or clindamycin 30-40mg/kg in three divided doses	Not applicable	None<br>Not applicable
Netherlands	Amoxicillin 40mg/kg/day in 3 divided doses	7 days	Second line and also treatment failure:<br><br>Amoxicillin-clavulanic acid 40/10mg/kg/day in 3 divided doses for 7 days	Penicillin allergy: cotrimoxazole 36mg/kg/day in two divided doses; 5-7 days	Not applicable	Not applicable

For peer review only

Norway	PO phenoxymethylpenicillin 24-60mg/kg/day in 3-4 divided doses per day<br><br>In case of frequent/recurent cases:<br>Amoxicillin 21-42mg/kg/day in three divided doses	5 days	Treatment failure:<br><br>trimetroprim sulfamethoxazole (for children)	Allergy:<br><25kg:<br>Erythromycin oral solution (ethyl succinate) 40mg/kg/day in two divided doses for 5 days<br><br>or<br>Clarithromycin (children over 6 months) 15 mg/kg/day in two divided doses for 5 days<br><br>25-35kg:<br>Erythromycin enteric capsules 500mg/kg/day in two divided doses for 5 days<br>or<br>Clarithromycin 14 mg/kg/day in two divided doses for 5 days	None	None
Poland	PO amoxicillin<br><40kg: 75-90mg/kg/day in 2 divided doses<br><br>>40kg 3000-4000mg per day in 2 divided doses	If <2 years of age, 10 days duration<br><br>If >2 years of age, 5 days duration	Treatment failure:<br>Amoxicillin/clavulanic acid<br><40kg: 70-90mg/kg/day in two divided doses<br><br>>40kg: 3000mg-4000mg per day in 2 divided doses<br><br>IVceftriaxone	Allergy to amoxicillin:<br>PO Cefuroxime axetil<br>>40kg 1000mg/day in 2 divided doses for 5 days<br><40kg 30mg/kg/day in two divided doses for 5 days unless if <2 yoa then for 10 days<br><br>Allergy to amoxicillin and severe infection:<br>Ceftriaxone<br>>40kg- 1-2g IV/IM per day once daily for 3 days	First line:<br>Level II /Grade A<br><br>First line duration: Level II /Grade B<br><br>Treatment failure:<br>Level II-III/ Grade A-B	First line antibiotic: Level 2a and 3a<br><br>First line duration: Level 2a and 3a<br><br>Treatment failure: Level 2a, 3a, 4, and 5

For peer review only

			<40kg 50mg/kg/day once daily for 3 days<br><br>>40kg 1-2g/day once daily for 3 days	<40kg- 50mg/kg IV/IM per day once daily for 3 days<br><br>Allergy to beta lactams: Clarithromycin<br><40kg 15-20mg/kg/day in two divided doses;<br>>40kg 500-1000mg/day in two divided doses	Allergy treatment: Level I-II/ Grade A-B	Allergy treatment: 1a, 2a, 3a
Portugal	Amoxicillin 80-90mg/day in 2 divided doses	5 days routine or <2 years of age<br>7 days if <2 years, recurrent, failure of initial treatment<br>10 days if recurrent AOM	<u>Treatment failure:</u><br><br>PO/IV Amoxicillin and clavulanic acid 80-90mg/kg/day in 2 divided doses or<br><br>PO Cefuroxime-axetil 30mg/kg/day in 2 divided doses or IV 80-100mg/kg/day in 3dividied doses<br>7 days if <2 years<br><br>OR<br><br>IM/IV Ceftriaxone 50mg/kg/day once daily	<u>Penicillin allergy:</u><br>Clarithromycin 50mg/kg/day in 2 divided doses or<br><br>Erythromycin 50mg/kg/day in 3-4 divided doses per day or<br><br>Azithromycin 10mg/kg/day once a day	First line: Level A/ Grade 1<br><br>Treatment failure: Level B/ Grade IIa<br><br>Duration:<br>7 days- Level A / Grade IIa<br><br>5 days- Level A/ Grade 1<br><br>10 days no evidence<br><br>Allergic treatment: Level C/Grade I	First line: level 1a, 2a, or 3a<br><br>Treatment failure: Level 1b, 2b, or 3b<br><br>Duration:<br>7 days- level 1a, 2a, or 3a<br><br>5 days- level 1a, 2a, or 3a<br><br>10 days no evidence<br><br>Allergic treatment Level 4-5 s
Spain	PO Amoxicillin 80-90mg/kg/day in 3 divided doses<br><br><u>If <6 months, severe symptoms, family history of</u>	Routine: 5 days<br><br>Otherwise: 10 days	<u>Treatment failure:</u><br><br>Amoxicillin-clavulanic acid 80-90mg/kg/day in 3 divided doses for 7-10 days or IM/IV	<u>Penicillin allergy:</u><br>Cefuroxime axetil 30mg/kg/day in 2 divided doses<br><br><u>If anaphylaxis to penicillin:</u>	First line: Level II/ Grade B<br><br>For children requiring amoxicillin-	First line: 2a<br><br>For children requiring amoxicillin-clavulanic acid

For peer review only

	ENT complications, previous therapeutic failure<br>Amoxicillin/clavulanic acid 80-90mg/kg/day in 3 divided doses for 7-10 days<br><br>If <2 months of age:<br>IV cefotaxime or PO/ IV amoxicillin-clavulanic acid. For PO only if no fever or no symptoms.<br>No LOE		Ceftriaxone 50mg/kg/day for 3 days	Clarithromycin 15mg/kg/day in 2 divided doses for 7 days or azithromycin 10mg/kg once daily on first day, then 5mg/kg once daily for 4 additional days.<br>Can also give Levofloxacin<br>6 months-5 yoa: 10mg/kg every twelve hours<br><br>>5 yoa give 10mg/kg every 24 hours	clavulanic acid as first line: Level II/ Grade B (No evidence for <2 months of age)<br><br>Treatment failure: Level III/Grade C; for ceftriaxone Level I/Grade A<br><br>Allergic to penicillin/cephalosporin: Level III/Grade C	as first line: Level 2a-3a<br><br>Treatment failure: Level 4-5 ; Ceftraixone as used in treatment failure: Level 1a<br><br>Allergy to penicillin/cephalosporin: Level 4-5
Sweden	PO Penicillin V 75 mg / kg/day in 3 divided doses<br><br>If recurrent: (new acute otitis media within a month with symptom-free intervals): Penicillin V 75mg / kg/day in 3 divided doses or<br><br>Amoxicillin 60 mg / kg/day in 3 divided	Routine: 5 days<br><br>Recurrent: 10 days	Treatment failure:<br><br>Amoxycillin 60mg/kg/day in 3 divided doses for ten days.	Penicillin allergy:<br>Erythromycin 40mg/kg/day in 4 divided doses or 40mg/kg/day in 2 divided doses	Not applicable	Not applicable

For peer review only

	doses					
Switzerland	Amoxicillin 50mg/kg/day in 2 divided doses<br><br>If risk factors or from country with high rates of penicillin resistance: Amoxicillin 80mg/kg/day in 2 divided doses	Routine: 5 days<br><br><2 years of age, previous otitis media, perforated tympanic membrane or from country with high rates of penicillin resistance : 10 days	‡Amoxicillin/clavulanic acid 80mg/kg/day in two divided doses for 10 days ‡ or<br><br>Ceftriaxone 50mg/kg daily for 1-3 days	None	Not applicable	Not applicable
UK SIGN (refers to BNF for Children)	Amoxicillin<br><br>Child 1-11 months of age: 125mg three times a day for 5-7 days<br><br>Child 1-4 years: 250mg three times a day for 5-7 days<br><br>(NB: dosage can be both low and high dose amoxicillin dependently on kg; for example If 1 yoa and weight <50th centile, will be given high dose; if 2 yoa and weight at 50th centile, would be given low dose.	5-7 days	Treatment failure: Amoxicillin-clavulanic acid<br><br>(dose dependent on suspension available)	Penicillin allergy: Clarithromycin or erythromycin ; dosage dependent on age	None	None

For peer review only

USA	Amoxicillin (80–90 mg/ kg/ day in 2 divided doses<br><br>OR<br><br>If amoxicillin past 30 days, concurrent purulent conjunctivitis (otitis conjunctivitis syndrome), or history of AOM non responsive to amoxicillin:<br><br>Amoxicillin-clavulanic acid (90 mg/kg/ day amoxicillin dosage in 2 divided doses	<2 years of age: 10 days<br><br>2-5 years of age:7 days<br><br>>6 years of age 5-7 days	Treatment failure:<br><br>Amoxicillin-clavulanic acid 90mg/kg/day 12 hourly<br><br><2 years of age: 10 day course<br><br>2-5 years of age 7 day course<br><br>>6 years of age 5-7 days<br><br>OR<br><br>IM/IV Ceftriaxone 50mg daily 3 days	Penicillin allergy:<br><br>Cefdinir 14mg/kg per day in 1-2 divided doses or cefuroxime 30mg/kg per day in 2 divided doses or cefpodoxime 10mg/kg per day in 2 divided doses or ceftriaxone 50mg IM or IV per day for 1-3 days	First line : B, recommendation; first line amoxil-clav: C, recommendation<br><br>Treatment failure: B, recommendation	First line: 2a or 3a<br><br>First line amoxi clav: 2a, 3a, 4<br><br>Treatment failure: 2a, 3a
WHO	PO amoxicillin 80mg/kg/day in 2 divided doses<br><br>If consider pathogen sensitive, give co-trimaxazole, dose: (trimethoprim component 8mg/kg/day 12 hourly for 5 days)	7-10 days	Repeat antibiotics for another 5 days	None	Low quality evidence/ Strong recommendation	Level 4

NB Amoxicillin-clavulanic acid dose always given in terms of amoxicillin component dosage.

For peer review only

* Routinely to treat with low-dose amoxicillin, but for high dose amoxicillin in the following situations: Switzerland: If from area of high penicillin resistance, age <6 months, severe symptoms. UK: "If necessary." Germany: area of high penicillin resistance
† Italy risk factors: risk factors for bacterial resistance: age <3 years, day-care attendance, older siblings, recent antibiotic therapy (<1 month), no PCV-7. ‡ Switzerland: If <6 months, severe symptoms, family history of ENT complications, previous therapeutic failure, recent administration of amoxicillin, concurrent purulent conjunctivitis or otorrhoea , region of high penicillin resistance or risk factors for antibiotic resistant for PO amoxicillin-clavulanic acid as first line.

11) AGREE scores by country (%)

Guideline	Domain 1	Domain 2	Domain 3	Domain 4	Domain 5	Domain 6	Mean National Score
Belgium	100	56	64	81	10	29	57
Czech Republic	36	25	4	21	0	0	14
Denmark	97	64	83	100	56	54	76
Finland	92	50	59	97	40	42	63
France	64	0	3	83	4	0	26
Germany	83	61	43	92	35	96	68
Ireland	11	3	1	56	2	0	12
Italy	100	83	63	97	40	46	72
Luxembourg	58	6	6	58	4	0	22
Netherlands	69	81	74	92	25	83	71
Norway	53	50	14	86	13	0	36
Poland	67	42	27	92	21	13	44
Portugal	44	33	18	64	33	8	33

For peer review only

Spain	44	28	22	83	15	29	37
Sweden	64	25	9	83	31	58	45
Switzerland	8	0	0	56	0	0	11
UK SIGN	92	92	82	92	58	29	74
European mean	*64*	*41*	*34*	*78*	*23*	*29*	*29*
AAP	97	67	88	89	35	54	72
WHO	94	58	80	92	60	83	78

12) AGREE scores by marker

a) HS AGREE scores

	**Domain 1**			**Domain 2**			**Domain 3**								**Domain 4**			**Domain 5**				**Domain 6**	
Criteria	**1**	**2**	**3**	**4**	**5**	**6**	**7**	**8**	**9**	**10**	**11**	**12**	**13**	**14**	**15**	**16**	**17**	**18**	**19**	**20**	**21**	**22**	**23**
Belgium	7	7	7	4	3	4	7	7	7	6	5	7	1	1	6	7	5	1	4	1	1	2	3
Czech Republic	7	1	1	6	1	1	1	1	1	1	1	1	3	1	7	4	7	1	1	1	1	1	1
Denmark	6	7	7	5	1	6	7	6	7	5	7	6	7	5	7	7	7	3	7	5	7	5	7
Finland	7	6	6	6	1	6	7	5	7	6	6	5	5	5	6	7	7	7	6	6	4	5	7
France	3	3	5	1	1	1	1	1	1	1	3	1	1	1	5	5	5	2	2	1	1	1	1
Germany	6	5	5	5	1	7	1	5	6	2	6	5	1	7	7	6	6	6	4	5	4	7	7
Ireland	1	1	1	1	1	1	1	1	1	1	2	1	1	1	5	4	6	1	1	1	2	1	1

For peer review only

Italy	7	7	7	7	7	7	7	6	5	6	6	6	6	4	6	7	7	6	5	5	3	5	6
Luxembourg	2	2	7	1	1	1	1	1	1	1	1	1	1	1	5	4	4	3	1	1	1	1	1
Netherlands	3	2	7	6	6	6	7	7	7	5	7	5	7	1	7	6	7	6	4	5	1	7	7
Norway	7	1	4	5	1	7	1	1	1	1	1	4	3	3	6	7	7	3	3	1	2	1	1
Poland	5	4	2	3	1	7	4	1	5	1	6	4	1	1	6	6	7	5	4	4	1	1	1
Portugal	2	1	7	1	1	4	1	1	2	1	2	3	1	4	4	3	3	3	5	2	6	1	3
Spain	4	2	2	5	2	1	1	2	3	1	4	7	1	1	6	6	5	5	2	3	1	1	5
Sweden	6	5	7	6	1	1	1	1	5	1	5	1	1	1	5	7	7	7	5	4	1	5	7
Switzerland	1	1	1	1	1	1	1	1	1	1	1	1	1	1	5	3	6	1	1	1	1	1	1
UK SIGN	6	5	7	6	1	7	7	6	6	7	7	6	7	5	6	6	6	7	5	5	7	3	3
AAP	7	7	7	7	1	7	6	7	6	7	6	7	6	5	5	6	7	5	6	6	2	1	7
WHO	7	7	5	5	1	7	7	5	7	5	6	5	7	6	6	5	7	6	7	7	2	6	7

b) JED AGREE Scores

	Domain 1			Domain 2			Domain 3								Domain 4			Domain 5				Domain 6	
**Criteria**	**1**	**2**	**3**	**4**	**5**	**6**	**7**	**8**	**9**	**10**	**11**	**12**	**13**	**14**	**15**	**16**	**17**	**18**	**19**	**20**	**21**	**22**	**23**
Belgium	7	7	7	6	4	5	7	7	7	4	4	5	1	1	6	7	4	1	1	1	3	3	3
Czech Republic	5	1	4	5	1	1	1	1	1	1	1	2	2	1	4	7	7	1	1	1	1	1	1
Denmark	7	7	7	7	3	7	7	7	6	1	7	6	5	6	7	7	7	1	6	1	5	1	4
Finland	7	6	7	6	1	4	5	1	5	1	1	7	1	6	7	7	7	1	1	1	1	1	1
France	7	4	7	1	1	1	1	1	1	1	2	1	1	1	7	7	7	1	1	1	1	1	1

For peer review only

Germany	6	7	7	6	2	7	1	1	1	6	5	1	2	7	6	7	7	1	2	2	1	6	7
Ireland	3	2	2	1	1	2	1	1	1	1	1	1	1	1	2	2	7	1	1	1	1	1	1
Italy	7	7	7	7	1	7	4	2	4	1	4	6	3	6	7	7	7	1	4	2	1	1	3
Luxembourg	5	4	7	3	1	1	1	1	1	1	5	3	1	1	6	7	5	1	1	1	1	1	1
Netherlands	6	7	6	6	4	7	7	7	7	4	6	5	4	1	6	7	6	1	1	1	1	5	5
Norway	6	2	5	5	1	5	1	1	1	1	1	2	2	5	5	7	5	1	3	1	1	1	1
Poland	7	5	7	3	1	6	2	1	1	1	5	7	1	1	6	7	7	1	1	1	1	4	1
Portugal	4	1	7	5	1	6	1	1	1	1	2	5	1	6	5	7	7	1	1	1	5	1	1
Spain	5	4	5	6	1	1	1	1	1	1	5	6	1	1	7	7	5	1	1	1	1	1	4
Sweden	4	3	4	5	1	1	1	1	1	2	1	1	1	1	3	7	7	3	1	1	1	1	5
Switzerland	2	3	1	1	1	1	1	1	1	1	1	1	1	1	2	5	5	1	1	1	1	1	1
UK SIGN	7	7	7	7	4	7	4	3	7	6	7	6	5	6	7	7	7	2	3	1	6	1	4
AAP	6	7	7	7	1	7	5	6	7	7	7	7	6	5	6	7	7	7	1	2	1	3	6
WHO	7	7	7	6	1	7	4	1	7	7	7	7	6	6	7	7	7	5	4	4	2	4	7

13) Acknowledgements

We would like to acknowledge Professor Mike Levin and our colleagues from the PERFORM consortium and all our contacts below who helped us in obtaining national guidelines:

National Experts:
Daniela Klobassa (Austria)
Jan Verbakel (Belgium)
Žaneta Jelčić (Croatia)
Stephanie Menikou (Cyprus)
Linda Alderson (Czech Republic)
Ivan Peychl (Czech Republic)
Juri Lindy Pedersen (Denmark)
Rikke Jorgensen (Denmark)
Piia Jogi (Estonia)
Eda Tamm (Estonia)
Irja Lutsar (Estonia)
Inga Ivaskeviciene (Estonia)
Niina Valtanen (Finland)
Esa Korpi (Finland)
Jean Christophe Mercier (France)
Romain Basmaci (France)
Ulrich von Both (Germany)
Florian Gothe (Germany)
Johanna Krone (Germany)
Christoph Bidlingmanier (Germany)
Veronika Louizou (Greece)
Konstatinos Kakleas (Greece)
Maeve Kelleher (Ireland)
Silvia Bressan (Italy)
Dace Zavadska (Latvia)
Simona Sabulyte (Lithuania)
Armand Biver (Luxembourg)
Simon Attard-Montalto (Malta)
David Pace (Malta)
Arne Martin Slatsve (Norway)
Ingebjørg Fagerli (Norway)
Maria Pokorska-Śpiewak (Poland)
Kacper Toczylowski (Poland)
Magdalena Marczyńska (Poland)

For peer review only

Carolina Costa (Portugal)
Irina Branescu (Romania)
Diana Moldovan (Romania)
Keti Vincek (Slovenia)
Frederico Maritinon Torres (Spain)
Giannos Orfanos (Sweden)
Irina Rivero (Spain)
Pablo Obando Pacheco (Spain)
Philipp Agyeman (Sweden)
Hermione Lyall (UK)
Ana Turkova (UK)

For peer review only

PRISMA 2009 Checklist

Section/topic	#	Checklist item	Reported on page #
**TITLE**			
Title	1	Identify the report as a systematic review, meta-analysis, or both.	1, 2, 3
**ABSTRACT**			
Structured summary	2	Provide a structured summary including, as applicable: background; objectives; data sources; study eligibility criteria, participants, and interventions; study appraisal and synthesis methods; results; limitations; conclusions and implications of key findings; systematic review registration number.	2
**INTRODUCTION**			
Rationale	3	Describe the rationale for the review in the context of what is already known.	4
Objectives	4	Provide an explicit statement of questions being addressed with reference to participants, interventions, comparisons, outcomes, and study design (PICOS).	N/A
**METHODS**			
Protocol and registration	5	Indicate if a review protocol exists, if and where it can be accessed (e.g., Web address), and, if available, provide registration information including registration number.	No protocol
Eligibility criteria	6	Specify study characteristics (e.g., PICOS, length of follow-up) and report characteristics (e.g., years considered, language, publication status) used as criteria for eligibility, giving rationale.	4-5
Information sources	7	Describe all information sources (e.g., databases with dates of coverage, contact with study authors to identify additional studies) in the search and date last searched.	4-5
Search	8	Present full electronic search strategy for at least one database, including any limits used, such that it could be repeated.	5, Appendix 1
Study selection	9	State the process for selecting studies (i.e., screening, eligibility, included in systematic review, and, if applicable, included in the meta-analysis).	5
Data collection process	10	Describe method of data extraction from reports (e.g., piloted forms, independently, in duplicate) and any processes for obtaining and confirming data from investigators.	5
Data items	11	List and define all variables for which data were sought (e.g., PICOS, funding sources) and any assumptions and simplifications made.	N/A
Risk of bias in individual studies	12	Describe methods used for assessing risk of bias of individual studies (including specification of whether this was done at the study or outcome level), and how this information is to be used in any data synthesis.	N/A
Summary measures	13	State the principal summary measures (e.g., risk ratio, difference in means).	N/A

For peer review only

PRISMA 2009 Checklist

Synthesis of results	14	Describe the methods of handling data and combining results of studies, if done, including measures of consistency (e.g., I^2) for each meta-analysis.	6

Section/topic	#	Checklist item	Reported on page #
Risk of bias across studies	15	Specify any assessment of risk of bias that may affect the cumulative evidence (e.g., publication bias, selective reporting within studies).	N/A
Additional analyses	16	Describe methods of additional analyses (e.g., sensitivity or subgroup analyses, meta-regression), if done, indicating which were pre-specified.	6
**RESULTS**			
Study selection	17	Give numbers of studies screened, assessed for eligibility, and included in the review, with reasons for exclusions at each stage, ideally with a flow diagram.	Figure 2
Study characteristics	18	For each study, present characteristics for which data were extracted (e.g., study size, PICOS, follow-up period) and provide the citations.	7
Risk of bias within studies	19	Present data on risk of bias of each study and, if available, any outcome level assessment (see item 12).	N/A
Results of individual studies	20	For all outcomes considered (benefits or harms), present, for each study: (a) simple summary data for each intervention group (b) effect estimates and confidence intervals, ideally with a forest plot.	N/A
Synthesis of results	21	Present results of each meta-analysis done, including confidence intervals and measures of consistency.	Page 6-10
Risk of bias across studies	22	Present results of any assessment of risk of bias across studies (see Item 15).	N/A
Additional analysis	23	Give results of additional analyses, if done (e.g., sensitivity or subgroup analyses, meta-regression [see Item 16]).	N/A
**DISCUSSION**			
Summary of evidence	24	Summarize the main findings including the strength of evidence for each main outcome; consider their relevance to key groups (e.g., healthcare providers, users, and policy makers).	12
Limitations	25	Discuss limitations at study and outcome level (e.g., risk of bias), and at review-level (e.g., incomplete retrieval of identified research, reporting bias).	12
Conclusions	26	Provide a general interpretation of the results in the context of other evidence, and implications for future research.	13-14
**FUNDING**			
Funding	27	Describe sources of funding for the systematic review and other support (e.g., supply of data); role of funders for the systematic review.	15

From: Moher D, Liberati A, Tetzlaff J, Altman DG, The PRISMA Group (2009). Preferred Reporting Items for Systematic Reviews and Meta-Analyses: The PRISMA Statement. PLoS Med 6(7): e1000097.

For peer review only

For peer review only - http://bmjopen.bmj.com/site/about/guidelines.xhtml

PRISMA 2009 Checklist

doi:10.1371/journal.pmed1000097

For more information, visit: www.prisma-statement.org.

For peer review only

For peer review only - http://bmjopen.bmj.com/site/about/guidelines.xhtml

BMJ Open

Clinical practice guidelines for acute otitis media in children: A systematic review and appraisal of European national guidelines

Journal:	*BMJ Open*
Manuscript ID	bmjopen-2019-035343.R1
Article Type:	Original research
Date Submitted by the Author:	31-Dec-2019
Complete List of Authors:	Suzuki, Hijiri; London School of Hygiene and Tropical Medicine Department of Clinical Research, l;<br>Dewez, Juan ; London School of Hygiene and Tropical Medicine, Clinical Research Department<br>Nijman, R; Imperial College London,<br>Yeung, Shunmay; London School of Hygiene and Tropical Medicine
**Primary Subject Heading**:	Paediatrics
Secondary Subject Heading:	Infectious diseases, Public health, Ear, nose and throat/otolaryngology
Keywords:	Protocols & guidelines < HEALTH SERVICES ADMINISTRATION & MANAGEMENT, INFECTIOUS DISEASES, Paediatric otolaryngology < OTOLARYNGOLOGY, PAEDIATRICS, PUBLIC HEALTH

SCHOLARONE™
Manuscripts

I, the Submitting Author has the right to grant and does grant on behalf of all authors of the Work (as defined in the below author licence), an exclusive licence and/or a non-exclusive licence for contributions from authors who are: i) UK Crown employees; ii) where BMJ has agreed a CC-BY licence shall apply, and/or iii) in accordance with the terms applicable for US Federal Government officers or employees acting as part of their official duties; on a worldwide, perpetual, irrevocable, royalty-free basis to BMJ Publishing Group Ltd ("BMJ") its licensees and where the relevant Journal is co-owned by BMJ to the co-owners of the Journal, to publish the Work in this journal and any other BMJ products and to exploit all rights, as set out in our licence.

The Submitting Author accepts and understands that any supply made under these terms is made by BMJ to the Submitting Author unless you are acting as an employee on behalf of your employer or a postgraduate student of an affiliated institution which is paying any applicable article publishing charge ("APC") for Open Access articles. Where the Submitting Author wishes to make the Work available on an Open Access basis (and intends to pay the relevant APC), the terms of reuse of such Open Access shall be governed by a Creative Commons licence – details of these licences and which Creative Commons licence will apply to this Work are set out in our licence referred to above.

Other than as permitted in any relevant BMJ Author's Self Archiving Policies, I confirm this Work has not been accepted for publication elsewhere, is not being considered for publication elsewhere and does not duplicate material already published. I confirm all authors consent to publication of this Work and authorise the granting of this licence.

For peer review only

TITLE PAGE

Title: Clinical practice guidelines for acute otitis media in children: A systematic review and appraisal of European national guidelines

Authors: Hijiri G Suzuki, Juan Emmanuel Dewez, Ruud G Nijman, Shunmay Yeung

Correspondence to:
Shunmay Yeung:
London School of Hygiene & Tropical Medicine, Keppel Street, London WC1E 7HT
Email: Shunmay.Yeung@lshtm.ac.uk Phone number: +44 2079272657

Affiliations:
Hijiri G Suzuki (HGS):
1. London School of Hygiene and Tropical Medicine, Clinical Research Department, Faculty of Infectious and Tropical Diseases, Keppel St, Bloomsbury, London WC1E 7HT, United Kingdom

Juan Emmanuel Dewez (JED):
1. London School of Hygiene and Tropical Medicine, Clinical Research Department, Faculty of Infectious and Tropical Diseases, Keppel St, Bloomsbury, London WC1E 7HT, United Kingdom

Ruud G Nijman (RGN):
1. Section of Pediatrics, Division of Infectious Diseases, Department of Medicine, Imperial College London, Norfolk Place, London, W2 1PG, United Kingdom

Shunmay Yeung (SY):
1. MARCH Centre for Maternal, Adolescent, Reproductive and Child Health, London School of Hygiene and Tropical Medicine, Clinical Research Department, Faculty of Infectious and Tropical Diseases, Keppel St, Bloomsbury, London WC1E 7HT, United Kingdom
2. Section of Pediatrics, Division of Infectious Diseases, Department of Medicine, Imperial College London, Norfolk Place, London, W2 1PG, United Kingdom

Key words:
Acute Otitis media
Europe
Guidelines
Children
Systematic review
Antibiotic stewardship

Main text word count:
2,573 excluding tables

For peer review only

ABSTRACT

Objectives:

To appraise European guidelines for acute otitis media (AOM) in children, including methodological quality, level of evidence and strength of recommendations (SoR), and consideration of antibiotic stewardship.

Design:

Systematic review of the literature

Data sources:

Three-pronged search of 1) Databases: Medline, Embase, Cochrane library, Guidelines International Network, and Trip Medical Database; 2) websites of European national paediatric associations and 3) contact of European experts. Data was collected between January 2017-February 2018.

Eligibility criteria:

National guidelines of European countries for the clinical management of AOM in children aged <16 years.

Data extraction and synthesis:

Data was extracted using tables constructed by the research team.
Guidelines were graded using AGREE II criteria. Level of Evidence (LoE) and Strength of Recommendations (SoR) were compared. Guidelines were assessed for principles of antibiotic stewardship.

Results:

AOM guidelines were obtained from 17 of the 32 EU/EFTA countries. The mean AGREE II score was ≤41% across most domains. Diagnosis of AOM was based upon similar signs and symptoms. The most common indication for antibiotics was tympanic membrane perforation/otorrhoea (14/15,93%). The majority (15/17;88%) recommended a watchful waiting approach to antibiotics. Amoxicillin was the most common first-line antibiotic (14/17;82%). Recommended treatment duration varied from five to ten days. Seven countries advocated high dose (75-90mg/kg/day) and five

For peer review only

For peer review only - http://bmjopen.bmj.com/site/about/guidelines.xhtml

low dose (30-60mg/kg/day) amoxicillin. Less than 60% of guidelines used a national or international scale system to rate level of evidence to support recommendations. Under half of the guidelines (7/17; 41%) referred to country-specific microbiological and antibiotic resistance data.

Conclusions:

Guidelines for managing AOM were similar across European countries. Guideline quality was mostly weak, and often did not refer to country-specific antibiotic resistance patterns. Co-ordinating efforts to produce a core guideline which can then be adapted by each country may help improve overall quality and contribute to tackling antibiotic resistance.

Strengths and limitations of this study:

- Strengths: The methodology includes the use of a comprehensive three-pronged search strategy with no language restrictions to identify guidelines from across Europe, the use of a standardized and internationally recognised guideline appraisal tool (AGREE II), the assessment of Leveles of Evidence and Strength of Recommendations, and the assessment of whether antibiotic stewardship, a key measure to reduce antimicrobial resistance (AMR), was considered.
- Limitations: The review focused only on AOM without complications; guidelines for complex otitis media requiring specialist (Ear Nose Throat) input were not included. Another limitation is the consideration of whether guidelines developers used country-specific AMR patterns to assess if the recommendations of antibiotics were based on AMR data. However, there is often wide heterogeneity in terms of AMR patterns within each country.

For peer review only - http://bmjopen.bmj.com/site/about/guidelines.xhtml

INTRODUCTION

Acute otitis media (AOM) is one of the commonest infections of childhood;[1,2] approximately 60% of children have had at least one episode by four years of age.[3] It is also one of the most frequently cited reasons for antibiotic prescription in children less than 3 years of age,[4,5] accounting for 14% of all antibiotic prescriptions in children in the UK.[6] While both bacterial and/or viral pathogens can cause AOM,[7,8] it is usually considered to be a bacterial complication of a viral upper respiratory tract infection.[9]

The rationale for antibiotic prescription includes symptom control,[10] and the prevention of rare but serious complications, including mastoiditis and meningitis.[11] However, studies show that up to 80% of cases resolve spontaneously without antibiotics[12,13] and antibiotics are associated with the risk of side effects including vomiting, diarrhoea, and rash.[13,14] In addition, the inappropriate use of antibiotics has been identified as one of the key drivers of antibiotic resistance, a global health priority.[15-17] Emerging research has also demonstrated that longer antibiotic courses can lead to higher risks of resistance. Thus, providing clear guidance on appropriate antibiotic use in terms of the indications, choice and duration is considered important to help reducing antibiotic resistance.[18]

To promote antibiotic stewardship, the World Health Organization (WHO) recommends the development of treatment guidelines and the monitoring of local antibiotic resistance to inform the choice of antibiotics.[19] National guidelines for the first-line management of AOM may play a vital role in antibiotic stewardship.[20] To our knowledge there has not been a systematic review of the quality and content of national guidelines for the management of AOM. The aim of this systematic review was to describe European guidelines for AOM in children, to assess their methodological quality, to describe their evidence-based strength of recommendations, and to assess whether they incorporate consideration of antibiotic stewardship.

METHODOLOGY

To ensure a comprehensive review of nationally endorsed guidelines, we used a three-pronged approach that included (1) a systematic database search; (2) a website search of European national societies; and (3) expert consultation.

For peer review only - http://bmjopen.bmj.com/site/about/guidelines.xhtml

Firstly, a systematic search of databases was carried out using Medline, Embase, Cochrane library, Guidelines International Network (G-I-N), and Trip Medical Database from April 2017 to February 2018. Search terms were a combination of two elements 1) Synonyms for “acute otitis media” AND 2) Synonyms for guidelines. Guidelines were included if they met the following eligibility criteria: 1) were pertaining to the management of simple AOM, excluding the management of chronic or complex otitis media cases requiring specialist (Ear Nose Threat specialist) input; 2) they were national guidelines or endorsed by the national medical society from a European Union (EU) or European Free Trade Area (EFTA) country; and 3) published from the year 2000 to present. The American Association of Paediatrics (AAP)[21] and the WHO[22] guidelines were also included for comparison as they are widely recognised and utilised internationally. The search included all European languages. An initial review of titles and abstracts was performed by one reviewer (HGS). Additionally, the bibliographies of all guidelines were examined to identify further relevant resources (HGS). Secondly, the websites of national paediatric associations listed by the European Paediatric Association/Union of National European Paediatric Societies and Associations (EPA/ UNEPSA) were hand searched (HGS). Finally, a network of paediatric partners across Europe were contacted (RGN, SY, JED, HGS) to verify if the identified guidelines were the most up to date and widely utilised, and in cases where we had not managed to locate any guidelines, to assist in obtaining them. The choice of search terms and final selection of full-text guidelines was performed by two reviewers (HGS, JED) (Supplementary Files 1-2). If multiple national guidelines were found, the guideline judged to be most up to date, comprehensive, and more commonly utilised in clinical practice was included after discussion between paediatrics partners and reviewers (HGS, JED). Data was extracted using tables constructed by the research team.

Patient and Public Involvement

This systematic review was performed without patient involvement.

Guideline Quality Assessment

The AGREE II Instrument was used independently by two reviewers (HGS, JED) to determine the quality of each national guideline.[23] This is a standardised instrument that appraises the methodological framework of guideline development. The six

For peer review only - http://bmjopen.bmj.com/site/about/guidelines.xhtml

domains assessed are 1) Scope and purpose 2) Stakeholder involvement 3) Rigour of development including evidence base 4) Clarity of presentation 5) Applicability and 6) Editorial independence. Domains were scored on a 1-7 scale; any score that varied by >3 out of 7 was discussed and revised if this was felt to be reasonable.

Level of evidence and Strength of recommendation

National scales for grading levels of evidence and strength of recommendation were converted to Oxford Centre for Evidence Based Medicine (OCEBM) levels of evidence (LoE) and Strength of Recommendations (SoR) (Supplementary Files 3-4). However heterogeneity between grading systems meant a meaningful comparison was difficult. Therefore in order to compare Level of Evidence (LoE) between guidelines, we reviewed 1) whether guidelines used a national/international scale of evidence, 2) whether principles of risk versus harm were assessed, 3) whether strengths and limitations of evidence were assessed, and 4) if evidence was linked to a strength of recommendation. To allow for more meaningful comparison between guidelines, we used our scores for AGREE II items 11, 9, and 12 for the above 2) 3) and 4) respectively. We converted Strength of Recommendations (SoR) into three categories: highest, moderate, and lowest grade, indicated by shading of results in tables (Legend of Table 1 and 2).

Antibiotic Stewardship

As we were unable to find a standard scoring system to assess if a clinical guideline includes consideration of antibiotic stewardship, we based our methodology on a study by Elias et al.[24] We thus proposed six principles that demonstrate consideration of antibiotic stewardship based upon the author's consensus opinion. The principles are the inclusion in the guideline of 1) diagnostic criteria; 2) criteria for initiation of antibiotic therapy; 3) dosage; 4) route of administration; 5) what percentage of antibiotic recommendations was based upon country-specific resistance patterns (i.e. if 2 of 3 recommended antibiotics were supported by country-specific antibiotic resistance data, 67% was awarded) and 6) whether guidelines recommending amoxicillin or amoxicillin-clavulanic acid based the recommended dosage on country-specific resistance data. These two antibiotics were chosen because in contrast to other antibiotics, a higher dosage is recommended to overcome resistant strains.[25]

For peer review only - http://bmjopen.bmj.com/site/about/guidelines.xhtml

RESULTS

Overview of existing guidelines

The search retrieved 7340 records (Figure 1). Of these, 19 guidelines were obtained. National guidelines were obtained from 17 of 32 European countries[26-42] (53%) (Figure 2), and two non-European countries/organisations (USA and WHO). The majority of these were from Western Europe and Scandinavia. The intended audience of the obtained guidelines was mainly general practitioners and paediatricians, although some included nurses or physician's assistants.. Four of seventeen European guidelines clearly stated they based their findings upon other national guidelines, including those of the American Academy of Paediatrics, French Agence Française de Sécurité Sanitaire des Produits de Santé (AFSSAPS, now known as Agence Nationale de Sécurité du Médicament et des Produits de Santé or ANSM), and UK Scottish Intercollegiate Guidelines Network (SIGN).

Diagnostic criteria

Fifteen of 17 (88%) European guidelines outlined the signs and symptoms for diagnosing AOM (Supplementary File 5) with considerable similarities between the guidelines. Twelve (71%) utilised strict combinations of three diagnostic criteria: 1) Acute onset of symptoms (i.e. otalgia, fever), 2) evidence of middle ear effusion (i.e. tympanic membrane (TM) bulging of tympanic membrane or otorrhea on examination and 3) Inflammation of TM on examination.

Otoscopy

Examination tools including standard otoscopy was advised by 15 (88%) European guidelines (Supplementary File 6). Pneumatic otoscopy (9/15; 60%)and tympanometry (7/15; 50%) were also recommended.

Additional investigations

No guidelines advised routine laboratory or radiographic investigations (Supplementary File 7). Nine of 17 (53%) guidelines stated specific indications for carrying out investigations. Eight of these advised consideration of a culture sample of the middle ear (ME) via tympanocentesis, most commonly for treatment failure (6/9;

For peer review only - http://bmjopen.bmj.com/site/about/guidelines.xhtml

67%) and complications such as mastoiditis (4/9; 44%). Three guidelines (3/9; 33%) discussed imaging modalities such as a CT brain when investigating secondary mastoiditis.

Approach to antibiotic administration

There were two approaches towards antibiotic administration: a watchful waiting approach and immediate antibiotic prescription (Table 1). Fifteen (88%) of the European guidelines recommended a watchful waiting approach where clinicians were encouraged to prescribe antibiotics if symptoms persisted for 1-3 days or if any clinical deterioration. Tympanic membrane perforation/otorrhea (14/15, 93%) and severity of symptoms (13/15, 87%) were the most common indication for immediate antibiotic administration (Table 2). WHO guidelines recommended all children with confirmed AOM be given antibiotics.

Table 1: Strength of recommendations supporting immediate or watchful waiting approach to antibiotic administration in European, AAP, and WHO guidelines

Treatment approach	Strength of Recommendation
Immediate antibiotics for any AOM	
WHO	Strong recommendation
Immediate antibiotics for any AOM can be considered	
Finland	A
USA	Recommendation
Czech Republic	No grade
Watchful waiting approach (except for indications outlined in Table 2)	
France	A
Italy	A
Spain	A
Denmark	√
Poland	B
Portugal	IIa
UK	B
Belgium	No grade
Germany	No grade

For peer review only - http://bmjopen.bmj.com/site/about/guidelines.xhtml

Ireland	No grade
Luxembourg	No grade
Netherlands	No grade
Norway	No grade
Sweden	No grade
Switzerland	No grade

Table 2: Indications for consideration of immediate antibiotic treatment in European and AAP guidelines

Guideline	Age (months)*	Parental input †	Uni-lateral AOM ‡	Bilateral AOM aged <24 months §	Severe symptoms ¶	Co-morbidities	Recurrent AOM	TM perfora-tion/ Otorrhoea
Italy	-	-	+	+	+	-	-	+
Spain	<24	-	-	+	+	-	+	+
Denmark	<6	-	-	+	+	-	-	+
France	<24	+	-	-	+	-	-	-
Portugal	<6	-	-	+	+	-	+	+
USA	-	+	+	+	+	-	-	-
Norway	<12	-	-	+	-	-	-	+
Poland	<6	+	+	+	+	+	+	+
Belgium	<6	-	-	+	+	+	-	+
Czech Republic	-	-	-	-	+	-	-	+
Finland	<24	-	-	+	-	-	-	+
Germany	<24	-	-	+	+	+	+	+
Ireland	-	-	-	-	-	-	-	+
Luxembourg	<24	-	-	-	+	-	-	-
Netherlands	<6	-	-	+	+	+	-	+
Sweden	<12	-	-	+	+	+	-	+
Switzerland	<24	-	-	+	+	+	+	+
UK	-	-	-	-	-	-	-	-

Highest grade
Moderate grade
Lowest grade
No grade

*Sweden: also children aged >12 years, Switzerland <24 months of age, only if the child appears unwell

† France: Give antibiotics if parents are considered unreliable. USA: join decision making with parents at any age. Poland: joint decision making with parents if child is <24 months of age

Legend: Table 1-2 and Figure 3

For peer review only - http://bmjopen.bmj.com/site/about/guidelines.xhtml

‡Unilateral: Italy: If age <6 months, Poland: if age <24 months then can give after joint decision making with parents

§Belgium, Finland and Sweden: bilateral at any age; Luxembourg: after consultation with parents

¶Symptoms include fever, otalgia, pain, vomiting and diarrhea. Switzerland: only if <24 months old

NB: The "-" indicates that those indications are not mentioned in the guideline.

First line antibiotic therapy

Fourteen of 17 (82%) European guidelines recommended oral amoxicillin as an option for first line treatment (Figure 3), of which seven (50%) recommended a high dose (75-90mg/kg/day), and five (36%) a low dose (30-60mg/kg/day). Stratification to high or low dose amoxicillin for children in the UK SIGN guideline is weight dependent; the Irish guidelines did not specify a dose. All the Nordic countries (i.e. Denmark, Sweden, and Norway) except Finland included Oral Penicillin V 24-75mg/kg/day as a first line choice (Supplementary File 8).

Treatment failure and penicillin allergy: Alternative antibiotic treatments

In case of treatment failure, amoxicillin-clavulanic acid per oral (PO) or intravenous (IV) (11/15, 73%), and IV/intramuscular (IM) ceftriaxone (8/15, 53%) were the most commonly recommended. In case of penicillin allergy, guidelines advised either PO clarithromycin (8/16; 50%) or PO trimethoprim-sulfamethoxazole (6/16; 38%) (Supplementary File 8).

Quality assessment: AGREE II scores

All guidelines were appraised using the AGREE Criteria (Table 3). In four of seven domains (i.e. 2, 3, 5, and 6), European guidelines obtained a mean score of ≤41% while only two domains (i.e. 1 and 4) scored above 63% (Supplementary File 9a-b)

Table 3: AGREE II scores (%) of European, AAP and WHO guidelines

Domain number	Domain name	European Mean (Range)	AAP Mean	WHO Mean
1	Scope and Purpose	57 (10-100)	97	94
2	Stakeholder involvement	41 (0-92)	67	58
3	Rigour of development	34 (0-83)	88	80
4	Clarity of presentation	78 (21-100)	89	92
5	Applicability	23 (0-58)	35	60
6	Editorial independence	29 (0-96)	54	83

For peer review only - http://bmjopen.bmj.com/site/about/guidelines.xhtml

Loe and SoR

Ten of 17 European guidelines (59%) based their certainty of evidence (i.e. Level of evidence-LoE) and Strength of Recommendations (SoR) upon a variety of methodologies (Table 4). The only crossover was between Poland and Spain, who utilised a methodology from the Infectious Diseases Society of America. AGREE II scores for quality of the LoE were variable, and approximately half of European guidelines (8/17; 47%) scored ≤4 across all items. SoR was often based upon study design (i.e. multiple RCTs) but for some was based on more subjective assessments (i.e. “well conducted studies”).

Table 4: Level of evidence (LoE) in AOM guidelines

Country	Scale of LoE used*	Score: Consideration of benefits and harms (AGREE II Item 11**)	Score: Strengths and limitations of the evidence (AGREE II Item 9)	Score: Link between recommendations and evidence (AGREE II Item 12)
Belgium	INAMI	5	7	6
Czech Republic	-	1	1	2
Denmark	OCEBM	7	7	6
Finland	Duodecim	1	6	6
France	ANAES	3	1	1
Germany	AWMF	6	3	3
Ireland	-	1	1	1
Italy	PNLG	5	5	6
Luxembourg	-	3	1	2
Netherlands	-	7	7	5
Norway	-	1	1	3
Poland	Infectious Disease Society of America	6	3	5
Portugal	European Society of Cardiology	2	2	4
Sweden	-	3	3	1
Switzerland	-	1	1	1
Spain	Infectious Disease Society of America	5	2	7
UK	SIGN	7	7	6
USA	AAP	7	7	7
WHO	GRADE	7	7	6

For peer review only - http://bmjopen.bmj.com/site/about/guidelines.xhtml

*If no LoE scale used, denoted by -
**AGREE II scores: 1= no information in the guideline; 7= exceptional reporting

Antibiotic stewardship

The majority of guidelines provided diagnostic criteria for AOM, specifications on when to start antibiotics, the route of administration and the duration of treatment (Table 5). However, less than half referred to country-specific AMR patterns, and four (24%) included both country-specific AMR data and specified resistance levels to amoxicillin/amoxicillin-clavulanic acid to guide local choices.

Table 5: Antibiotic stewardship and AOM guidelines

	Do guidelines provide diagnostic criteria?	Do guidelines specify when to initiate antibiotics?	Do guidelines specify route of administration?	Do guidelines specify duration of antibiotic regimens?	Do antibiotic recommendations refer to country-specific AMR patterns?		
					Percentage of antibiotic recommendations that refer to country-specific AMR patterns	Amoxicillin dosage that refer to country-specific AMR patterns	Amoxicillin-clavulanic acid dosage that refer to country-specific AMR patterns
Belgium	Yes	Yes	Yes	Yes	80%	Yes	Yes
Czech Rep.	Yes	Yes	Yes	Yes	0%	Unclear	Unclear
Denmark	Yes	Yes	Yes	Yes	0%	Not applicable	Unclear
Finland	Yes	Yes	Yes	Yes	62.50%	Yes	Yes
France	Yes	Yes	Yes	Yes	0%	Unclear	Unclear
Germany	Yes	Yes	Yes	Yes	0%	Unclear	Unclear
Ireland	Unclear	Yes	Yes	Unclear	0%	Unclear	Not applicable
Italy	Yes	Yes	Yes	Yes	67%	Yes	Yes
Luxembourg	Yes	Yes	Yes	Yes	0%	Unclear	Unclear
Netherlands	Yes	Yes	Yes	Yes	100%	Yes	Yes
Norway	Yes	Yes	Yes	Yes	0%	Unclear	Not applicable
Poland	Yes	Yes	Yes	Yes	100%	Yes	Not applicable
Portugal	Yes	Yes	Yes	Yes	71%	Yes	Yes
Promotes antibiotic stewardship							
Spain	Yes	Yes	Yes	Yes	100%	Yes	Yes
Sweden	Yes	Yes	Yes	Yes	100%	Unclear	Not applicable
Switzerland	Unclear	Yes	Yes	Yes	0%	Unclear	Unclear
UK	Yes	Yes	Yes	Yes	0%	Unclear	Unclear
USA	Yes	Yes	Yes	Yes	100%	Yes	Yes
WHO	Yes	Yes	Yes	Yes	Not applicable	Not applicable	Not applicable

Legend:

For peer review only - http://bmjopen.bmj.com/site/about/guidelines.xhtml

Partially promotes antibiotic stewardship
Does not promote antibiotic stewardship

DISCUSSION

Approximately half of the 32 EU/EFTA countries have AOM guidelines. Diagnosis of AOM was based upon similar signs and symptoms. Tympanocentesis was commonly reserved for treatment failure. The vast majority of European guidelines advocated for a watchful waiting approach to antibiotic therapy with the most common indications for treatment being tympanic membrane perforation, and severity of symptoms. Amoxicillin was the most commonly recommended first-line antibiotic, but with differences in terms of recommended duration and dosage. Our quality assessment found low mean AGREE II scores of ≤41% in most domains. Less than 60% of guidelines used a national or international scale system to rate level of evidence to support recommendations. Less than half of the guidelines referred to country-specific patterns of AMR.

Strengths of our study include the comprehensiveness of our three-pronged search strategy, the use of AGREE-II, an internationally recognised guideline appraisal tool, and an assessment of which LoE and SoR were used. Our analysis also included a qualitative assessment of whether antibiotic stewardship was considered in the development of guidelines, based on five criteria. In order to provide a broad sense on whether AMR data were considered, one of the criteria was whether the antibiotic recommendations referred to country-specific AMR data. However, the limitation of this as a criteria is that there is often wide heterogeneity in terms of AMR patterns within each country, and, as well as referring to the AMR data on which the antibiotic recommendation is based, guidelines should ideally recommend that the antibiotic choice should be adapted based on local AMR data, if available. Another limitation is our focus on simple AOM and exclusion of guidelines about complex cases requiring Ear Nose Throat specialist input.

Previously published works demonstrated a common consensus in criteria for AOM diagnosis, and that watchful waiting period was the standard of care in Europe; amoxicillin was also found to be the most commonly recommended antibiotic.[43-45] In

For peer review only

For peer review only - http://bmjopen.bmj.com/site/about/guidelines.xhtml

comparison to these studies, our work aimed to compare additional facets of AOM management in Europe, including grading their quality, comparison of LoE and SoR, and assessing their inclusion of country-specific AMR data. Zeng *et al* also used AGREE II scores to assess quality of upper respiratory tract infections guidelines including three AOM guidelines from Japan, USA, and the UK.[46] We note a >10-point discrepancy in scoring in two of six domains between Zeng and ourselves for UK SIGN and US AAP AOM guidelines. This may indicate inter-user variability in AGREE II scoring.[47] Elias et al. assessed global infectious diseases guidelines and found that local AMR patterns were taken into account in 50-75% of recommendations, which is similar to our findings.

The development of clinical guidelines according to the high standards of the AGREEII criteria is a resource intensive exercise and this may be one of the reasons why we did not identify any guidelines from Eastern European countries. Many guidelines in this study received low AGREE II scores. Many of the resource intensive initial steps in guidelines development are universal, for example defining the objectives, the clinical questions, the target populations of patients and end-users; designing a comprehensive search strategy to identify relevant evidence from the literature, a process to appraise the evidence, a way to present recommendations unambiguously, and strategies to successfully implement guidelines. Replicating this process in each country to reach similar conclusions does not seem necessary nor efficient, and it may make sense for these or some of these processes to be undertaken by a core group of experts from across Europe. This is already the case for other medical specialities, for example, the European Joint Task Force for cardiovascular disease prevention provides guidelines that can be used across Europe.[48] The centrally-developed guidelines could then be adapted in each country for recommendations, such as choice of antibiotics, which depends on local AMR patterns and immunisation coverages against the main pathogens causing AOM. This implies the implementation of robust epidemiological and standardised AMR surveillance systems in each country, which is currently underway with the support of international initiatives such as the European Centre for Disease Prevention and Control (ECDC) surveillance systems,[49] and the WHO Global Antimicrobial Resistance Surveillance System (GLASS).[50] Other aspects that could lead to local adaptation could be local care pathways, and user and patient preferences. This approach would allow the

For peer review only - http://bmjopen.bmj.com/site/about/guidelines.xhtml

development of guidelines of better quality and better adapted to local contexts, and might contribute to reducing the spread of AMR.

CONCLUSION

Review of guidelines reveals major similarities in AOM management recommendations across Europe. Existing European guidelines scored poorly in most AGREE II domains, including items related to how evidence was gathered and appraised. Consideration of country-specific antibiotic resistance patterns appears to be limited. Centrally produced guidelines adapted for local care pathways, user and patient preferences, as well as for local antimicrobial resistance patterns may provide more targeted recommendations, reduce unnecessary antibiotic administration, and help reducing the spread of antibiotic resistance.

ACKNOWLEDGEMENTS

We would like to acknowledge Professor Mike Levin and our colleagues from the PERFORM consortium and all our contacts below who helped us in obtaining national guidelines:

Daniela Klobassa (Austria)
Jan Verbakel (Belgium)
Žaneta Jelčić (Croatia)
Stephanie Menikou (Cyprus)
Linda Alderson (Czech Republic)
Ivan Peychl (Czech Republic)
Juri Lindy Pedersen (Denmark)
Rikke Jorgensen (Denmark)
Piia Jogi (Estonia)
Eda Tamm (Estonia)
Irja Lutsar (Estonia)
Inga Ivaskeviciene (Estonia)
Niina Valtanen (Finland)
Esa Korpi (Finland)
Jean Christophe Mercier (France)
Romain Basmaci (France)
Ulrich von Both (Germany)
Florian Gothe (Germany)
Johanna Krone (Germany)
Christoph Bidlingmanier (Germany)
Veronika Louizou (Greece)
Konstatinos Kakleas (Greece)
Maeve Kelleher (Ireland)

For peer review only

Silvia Bressan (Italy)
Dace Zavadska (Latvia)
Simona Sabulyte (Lithuania)
Armand Biver (Luxembourg)
Simon Attard-Montalto (Malta)
David Pace (Malta)
Arne Martin Slatsve (Norway)
Ingebjørg Fagerli (Norway)
Maria Pokorska-Śpiewak (Poland)
Kacper Toczylowski (Poland)
Magdalena Marczyńska (Poland)
Carolina Costa (Portugal)
Irina Branescu (Romania)
Diana Moldovan (Romania)
Keti Vincek (Slovenia)
Frederico Maritinon Torres (Spain)
Giannos Orfanos (Sweden)
Irina Rivero (Spain)
Pablo Obando Pacheco (Spain)
Philipp Agyeman (Sweden)
Hermione Lyall (UK)
Ana Turkova (UK)

ADDITIONAL REQUIREMENTS

Ethics Approval

Not applicable

Transparency Declaration

The lead author Hijiri G Suzuki* affirms that this manuscript is an honest, accurate, and transparent account of the study being reported; that no important aspects of the study have been omitted; and that any discrepancies from the study as planned (and, if relevant, registered) have been explained.

*The manuscript's guarantor.

Funding

HGS did not receive any funding. RGN was supported by NIHR Academic clinical fellowship and lectureship award programme. JED and SY are supported by PERFORM, a consortium funded by the European Union's Horizon 2020 programme, under grant agreement No. 668303. The funding sources did not take part in the design, analysis, interpretation of data, writing of the report or decision to

For peer review only

For peer review only - http://bmjopen.bmj.com/site/about/guidelines.xhtml

submit the article for publication. All authors had full access to all the data, and can take responsibility for the integrity of the data and the accuracy of the data analysis.

Data Sharing Statement

The primary data for this study was treatment guidelines, these can be shared on request to the corresponding author

Competing Interests Declaration

No competing interests. All authors have completed the ICMJE uniform disclosure form at www.icmje.org/coi_disclosure.pdf and declare: no support from any organisation for the submitted work; no financial relationships with any organisations that might have an interest in the submitted work in the previous three years; no other relationships or activities that could appear to have influenced the submitted work.

Authorship Contributions:

SY conceived the study. HGS, JED, RGN and SY all contributed to the study design. HGS was responsible for the systematic database search. JED, RGN and SY all contacted experts in their scientific networks to obtain additional guidelines and check the use and validity of those identified. HGS and JED were responsible for data extraction including LoE, SoR and antibiotic sterwardship and AGREE II scoring. HGS, JED, RGN and SY all contributed to the interpretation of the results, the drafting and revision of the manuscript and agree with the final version.

For peer review only

References

1. Schilder AG, Chonmaitree T, Cripps AW, et al. Otitis media. *Nat Rev Dis Primers* 2016;2:16063. doi: 10.1038/nrdp.2016.63 27604644.
2. Palma S, Rosafio C, Del Giovane C, et al. The impact of the Italian guidelines on antibiotic prescription practices for acute otitis media in a paediatric emergency setting. *Ital J Pediatr* 2015;41:37. doi: 10.1186/s13052-015-0144-4.
3. Kaur R, Morris M, Pichichero ME. Epidemiology of Acute Otitis Media in the Postpneumococcal Conjugate Vaccine Era. *Pediatrics* 2017;140(3). doi: 10.1542/peds.2017-0181.
4. Coker TR, Chan LS, Newberry SJ, et al. Diagnosis, microbial epidemiology, and antibiotic treatment of acute otitis media in children: a systematic review. *Jama* 2010;304(19):2161-9. doi: 10.1001/jama.2010.1651.

For peer review only - http://bmjopen.bmj.com/site/about/guidelines.xhtml

5. Leibovitz E. Acute otitis media in pediatric medicine: current issues in epidemiology, diagnosis, and management. *Paediatr Drugs* 2003;5 (Suppl 1):1-12.
6. Thompson PL, Gilbert RE, Long PF, et al. Has UK guidance affected general practitioner antibiotic prescribing for otitis media in children? *J Public Health (Oxf)* 2008;30(4):479-86. doi: 10.1093/pubmed/fdn072.
7. Chen YJ, Hsieh YC, Huang YC, et al. Clinical manifestations and microbiology of acute otitis media with spontaneous otorrhea in children. *J Microbiol Immunol Infect* 2013;46(5):382-8. doi: 10.1016/j.jmii.2013.04.001.
8. Van Dongen TM, Venekamp RP, Wensing AM, et al. Acute otorrhea in children with tympanostomy tubes: prevalence of bacteria and viruses in the post-pneumococcal conjugate vaccine era. *Pediatr Infect Dis J* 2015;34(4):355-60. doi: 10.1097/inf.0000000000000595.
9. Nokso-Koivisto J, Marom T, Chonmaitree T. Importance of viruses in acute otitis media. *Curr Opin Pediatr.* 2015;27(1):110-15. doi10.1097/MOP.0000000000000184.
10. Rovers MM, Glasziou P, Appelman CL, et al. Antibiotics for acute otitis media: a meta-analysis with individual patient data. *Lancet* 2006;368(9545):1429-35. doi: 10.1016/s0140-6736(06)69606-2.
11. Groth A, Enoksson F, Hermansson A, et al. Acute mastoiditis in children in Sweden 1993-2007--no increase after new guidelines. *Int J Pediatr Otorhinolaryngol* 2011;75(12):1496-501. doi: 10.1016/j.ijporl.2011.08.015.
12. Rosenfeld RM, Vertrees JE, Carr J, et al. Clinical efficacy of antimicrobial drugs for acute otitis media: metaanalysis of 5400 children from thirty-three randomized trials. *J Pediatr* 1994;124(3):355-67. doi: 10.1016/s0022-3476(94)70356-6.
13. Venekamp RP, Sanders SL, Glasziou PP, et al. Antibiotics for acute otitis media in children. *Cochrane Database Syst Rev* 2015(6)CD000219. doi: 10.1002/14651858.CD000219.pub4.
14. Sakulchit T, Goldman RD. Antibiotic therapy for children with acute otitis media. *Can Fam Physician.* 2017;63(9):685-87.
15. Centres for Disease Control and Prevention. Antibiotic Resistance Threats in the United States, 2019. Atlanta, GA. U.S. Department of Health and Human Services, CDC; 2019. Available from www.cdc.gov/DrugResistance/Biggest-Threats.html (accessed 3 February 2017).
16. Costelloe C, Metcalfe C, Lovering A, et al. Effect of antibiotic prescribing in primary care on antimicrobial resistance in individual patients: systematic review and meta-analysis. *BMJ.* 2010;340:c2096. doi: 10.1136/bmj.c2096.
17. Sibbald AD. Acute otitis media in infants: the disease and the illness. Clinical distinctions for the new treatment paradigm. *Otolaryngol Head Neck Surg* 2012;147(4):606-10. doi: 10.1177/0194599812455780.
18. Pouwels KB, Llewelyn MJ, Walker AS, et al. Duration of antibiotic treatment for common infections in English primary care: cross sectional analysis and comparison with guidelines. *BMJ* 2019;364(l440). doi: 10.1136/bmj.l440
19. World Health Organisation. Global action plan on antimicrobial resistance, 2015. Geneva, Switzerland. Available from https://www.who.int/antimicrobial-resistance/publications/global-action-plan/en/ (accessed 7 June 2018).
20. Laxminarayan R, Duse A, Wattal C, et al. Antibiotic resistance-the need for global solutions. *Lancet Infect Dis* 2013;13(12):1057-98. doi: 10.1016/s1473-3099(13)70318-9.

For peer review only

For peer review only - http://bmjopen.bmj.com/site/about/guidelines.xhtml

21. Lieberthal AS, Carroll AE, Chonmaitree T, et al. The Diagnosis and Management of Acute Otitis Media. *Pediatrics* 2013;131(3):e964-e99. doi: 10.1542/peds.2012-3488 (accessed 15 January 2017).
22. World Health Organisation. Recommendations for management of common childhood conditions. Evidence for technical update of pocket book recommendations. Geneva, Switzerland. 2012. Available from https://www.who.int/maternal_child_adolescent/documents/management_childhood_conditions/en/ (accessed 20 January 2017).
23. Brouwers M, Kho ME, Browman GP, et al. for the AGREE Next Steps Consortium. AGREE II: Advancing guideline development, reporting and evaluation in healthcare. *CMAJ 2010;182(18):E839-42.* doi:10.1503/cmaj.090449.
24. Elias C, Moja L, Mertz D, et al. Guideline recommendations and antimicrobial resistance: the need for a change. *BMJ Open* 2017;7(7):e016264. doi: 10.1136/bmjopen-2017-016264.
25. Opatowski L, Mandel J, Varon E, et al. Antibiotic dose impact on resistance selection in the community: a mathematical model of beta-lactams and Streptococcus pneumoniae dynamics. *Antimicrob Agents Chemother* 2010;54(6):2330-37. doi: 10.1128/AAC.00331-09.
26. Institut National D'Assurance Maladie-invalidité. L'usage rationnel des antibiotiques chez l'enfant en ambulatoire, 2016. [The rational usage of antibiotics in ambulating children] Available from https://www.inami.fgov.be/SiteCollectionDocuments/consensus_texte_court_20160602.pdf (accessed 5 January 2018).
27. Bébrová EJB, Cizek H, Vaclav D, et al. Doporučený postup pro antibiotickou léčbu komunitních respiračních infekcí v primární péči.[The recommended procedure for antibiotic treatment of community respiratory infections] *Vox pediatrae* 2011; 11(2).
28. Dansk Selskab for Almen Medicin. Luftvejsinfektioner.− diagnose og behandling 2014. [Respiratory tract infections- diagnosis and treatment 2014). Available from http://vejledninger.dsam.dk/luftvejsinfektioner/ (accessed10 January 2017).
29. Suomalainen Lääkäriseura Duodecim. Välikorvatulehdus (lasten äkillinen). [Otitis media sudden onset of children]. 2017. Available from https://www.kaypahoito.fi/hoi31050 (accessed 12 December 2017).
30. Agence française de sécurité sanitaire des produits de santé. Recommandations de Bonne Pratique- Antibiothérapie par voie générale en pratique courante dans les infections respiratoires hautes [Recommendations for good practice- Antimicrobials by general route in current practice in upper respiratory tract infections]. 2011. Available from http://www.infectiologie.com/UserFiles/File/medias/Recos/2011-infections-respir-hautes-recommandations.pdf (accessed 10 April 2017).
31. Deutsche Gesellschaft für Allegemeinmedizin und Familienmedizin (DEGAM). DEGAM-Leitlinie Nr 7-Ohrenschmerzen [DEGAM guideline Number 7-Earache]. 2014. Available from https://www.awmf.org/en/clinical-practice-guidelines/search-for-guidelines.html#result-list (accessed 4 April 2017).
32. Health Services Executive and Royal College of Physicians Ireland. Acute Otitis Media. 2012. Available from https://www.hse.ie/eng/services/publications/clinical-strategy-and-programmes/paediatrics-acute-otitis-media.pdf (accessed 12 April 2017).

For peer review only - http://bmjopen.bmj.com/site/about/guidelines.xhtml

33. Marchisio P, Bellussi L, Di Mauro G, et al. Acute otitis media: From diagnosis to prevention. Summary of the Italian guideline. *Int J Pediatr Otorhinolaryngol* 2014;74:1209-16. doi:10.1016/j.ijporl.2010.08.016.
34. Conseil Scientifique Domaine de la Santé. Otite moyenne aiguë. [Acute Otitis media]. Luxembourg, Luxembourg. 2017.
35. Damoiseaux RAMJ, Venekamp RP, Eekhof JAH, et al. NHG Standaard Otitis media acuta bij kinderen [NHG Standard Otitis media acute in children]. *Huisarts Wet* 2014; 57(12)648. Available from https://www.nhg.org/standaarden/volledig/nhg-standaard-otitis-media-acuta-bij-kinderen (accessed October 15, 2017).
36. Antibiotikasenteret for primærmedisin. Akutt otitis media [Acute otitis media]. 2016. Available from http://www.antibiotikaiallmennpraksis.no/index.php?action=showtopic&topic=VMpmsqDE\ (accessed 5 January 2017).
37. Hryniewicz W, Piotr Albrecht, P., Radzikowski, A et al. Rekomendacje postępowania w pozaszpitalnych zakażeniach układu oddechowego. [Recommendations for management of in-hospital respiratory infections]. 2016. Available from http://antybiotyki.edu.pl/ (accessed 10 July 2017).
38. Departamento da Qualidade na Saúde. Diagnóstico e tratamento da Otite Média Aguda na Idade Pediátrica. [Diagnosis and Treatement of Acute Otitis Media in Paediatrics]. 2014. Available from https://www.dgs.pt/directrizes-da-dgs/normas-e-circulares-normativas/norma-n-0072012-de-16122012-png.aspx (accessed 15 April 2017).
39. Del Castillo Martín F, Bacquero Artigao F, de la Calle Cabrera T, et al. Asociación Española de Pediatría- Documento de consenso sobre etiología, diagnóstico y tratamiento de la otitis media aguda. [Consensus document on aetiology, diagnosis, and treatment of acute otitis media]. *An Pediatr (Barcelona)* 2012;77(5):8. doi: http://dx.doi.org/10.1016/j.anpedi.2012.05.026.
40. Läkemedelsverket. Diagnostik, behandling och uppföljning av akut mediaotit (AOM) – ny rekommendation. [Diagnosis, treatment and follow up of acute otitis media- new recommendations]. *Information från Läkemedelsverket* 2010;21:10-23. Available from https://lakemedelsverket.se/otit (accessed 5 January 2018).
41. Pediatric Infectious Disease Group of Switzerland. Recommendations pour le diagnostic et le traitement de Otite moyenne aiguë, Sinusite aiguë, Pneumonie (community-acquired), et Pharyngo-amygdalite chez l'enfant. [Recommendations for the diagnosis and treatment of acute otitis media, acute sinusitis, pneumonia (community acquired), and pharyngitis in children]. 2010. Available from http://www.pigs.ch/pigs/frames/documentsframe.html (accessed 2 May 2017).
42. Scottish Intercollegiate Guidelines Network. Diagnosis and management of childhood otitis media in primary care: A national clinical guideline. Edinburgh, UK. 2003.
43. Ovnat TS, Shemesh S, Oron Y, et al. Acute otitis media guidelines in selected developed and developing countries: uniformity and diversity. *Arch Dis Child* 2017;102(5):450-57. doi: 10.1136/archdischild-2016-310729.
44. Andre M AI, Hemlin C, Nasta F. Behandling av akut otitis media i andra länder - översikt över 28 nationella riktlinjer [Treatment of acute otitis media in other countries- a survey of 28 national guidelines]. *Information från Läkemedelsverket* 2010;21(5):37-43.

For peer review only - http://bmjopen.bmj.com/site/about/guidelines.xhtml

45. Toll EC, Nunez DA. Diagnosis and treatment of acute otitis media: review. *J Laryngol Otol* 2012;126(10):976-83. doi: 10.1017/s0022215112001326.
46. Zeng L, Zhang L, Hu Z, et al. Systematic review of evidence-based guidelines on medication therapy for upper respiratory tract infection in children with AGREE instrument. *PLoS One* 2014;9(2):e87711 doi:10.1371/journal.pone.0087711.
47. Marciano NJ, Merlin TL, Bessen T, et al. To what extent are current guidelines for cutaneous melanoma follow up based on scientific evidence? *Int J Clin Pract* 2014;68(6):761–770. doi:10.1111/ijcp.12393.
48. Piepoli MF, Hoes AW, Agewall S, et al. 2016 European Guidelines on cardiovascular disease prevention in clinical practice: The Sixth Joint Task Force of the European Society of Cardiology and Other Societies on Cardiovascular Disease Prevention in Clinical Practice (constituted by representatives of 10 societies and by invited experts) Developed with the special contribution of the European Association for Cardiovascular Prevention & Rehabilitation (EACPR). *Eur Heart J* 2016;37(29):2315-81. doi: 10.1093/eurheartj/ehw106.
49. European Centre for Disease Prevention and Control. TESSy- The European Surveillance System: Antimicrobial resistance (AMR) reporting protocol 2018. Stockholm, Sweden. Available from https://www.ecdc.europa.eu/sites/default/files/documents/EARS-Net%20reporting%20protocol%202018.%20docx.pdf (accessed 10 December 2019).
50. World Health Organisation. Global antimicrobial resistance surveillance system (GLASS) report: early implementation 2017-2018. Geneva, Switzerland. Available from https://www.who.int/glass/resources/publications/early-implementation-report-2017-2018/en/ (accessed 10 December 2019).

LEGENDS

Figure 1: PRISMA flow diagramme

Figure 2: European AOM guidelines (lead group and year published)

National guideline found
National guideline not found
No expert contacts

Figure 3: Routine first line antibiotics: Initiation, choice, duration and Strength of Recommendation

High dose amoxicillin
Low dose amoxicillin
Penicillin V
Low dose Amoxicillin/Amoxicillin-clavulanic acid
Variable low dose/high dose amoxicillin (weight dependent)
- Consider prolonged course

For peer review only - http://bmjopen.bmj.com/site/about/guidelines.xhtml

Figure 1: PRISMA Flow Diagramme

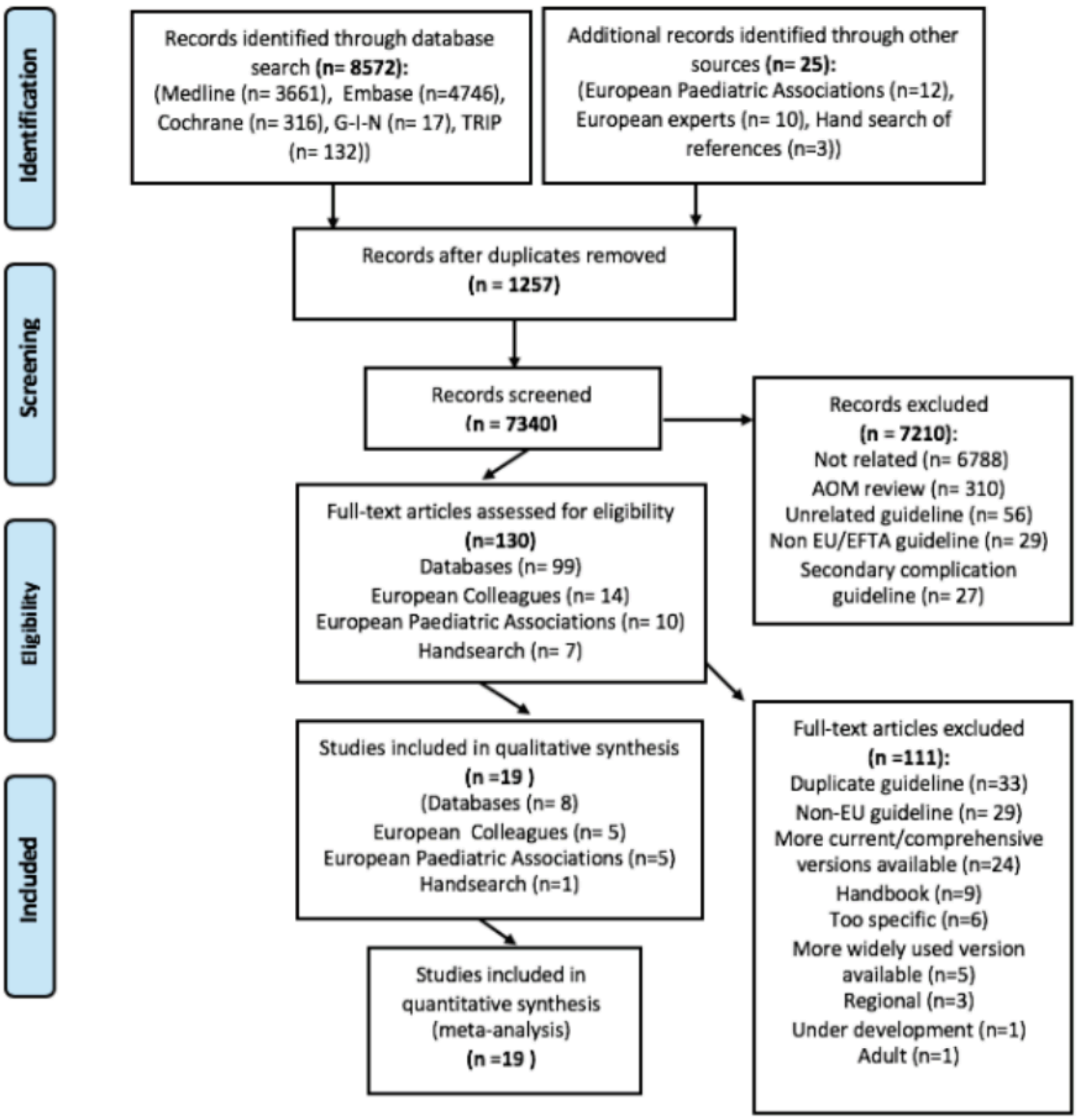

From: Moher D, Liberati A, Tetzlaff J, Altman DG, The PRISMA Group (2009). *Preferred Reporting Items for Systematic Reviews and Meta-Analyses: The PRISMA Statement.* PLoS Med 6(7): e1000097. doi:10.1371/journal.pmed1000097

For more information, visit www.prisma-statement.org.

Figure 2: European AOM guidelines (Lead group and year published)

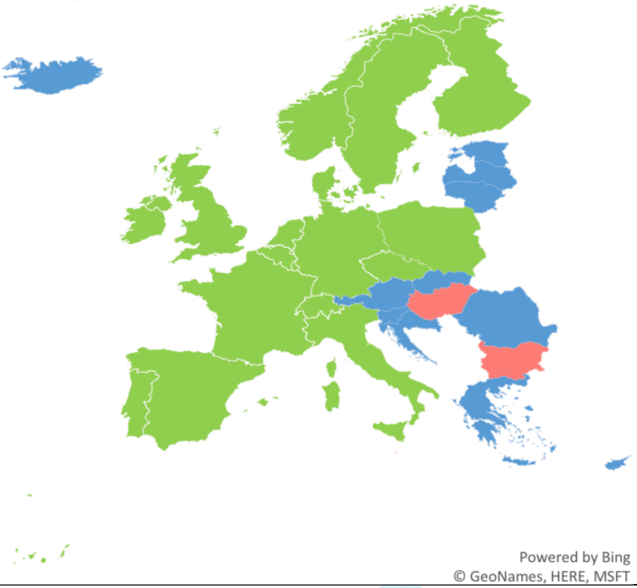

National guidelines found: Belgium (INAMI 2016), Czech Republic (CzMA 2011), Denmark (DSAM 2014),Finland (Duodecim 2017), France (AFSSAPS 2011), Germany (DEGAM 2014), Ireland (HSE 2012), Italy (SIP 2010), Luxembourg (CSDS 2007), Netherlands (NHG 2014), Norway (ASP 2016), Poland (NIL 2016), Portugal (DGS 2014), Spain (AEPED 2012), Sweden (MPA 2010), Switzerland (PIGS 2010), United Kingdom (SIGN 2003)

For peer review only

Figure 3: Routine first line antibiotics: Initiation, choice, duration and Strength of Recommendation

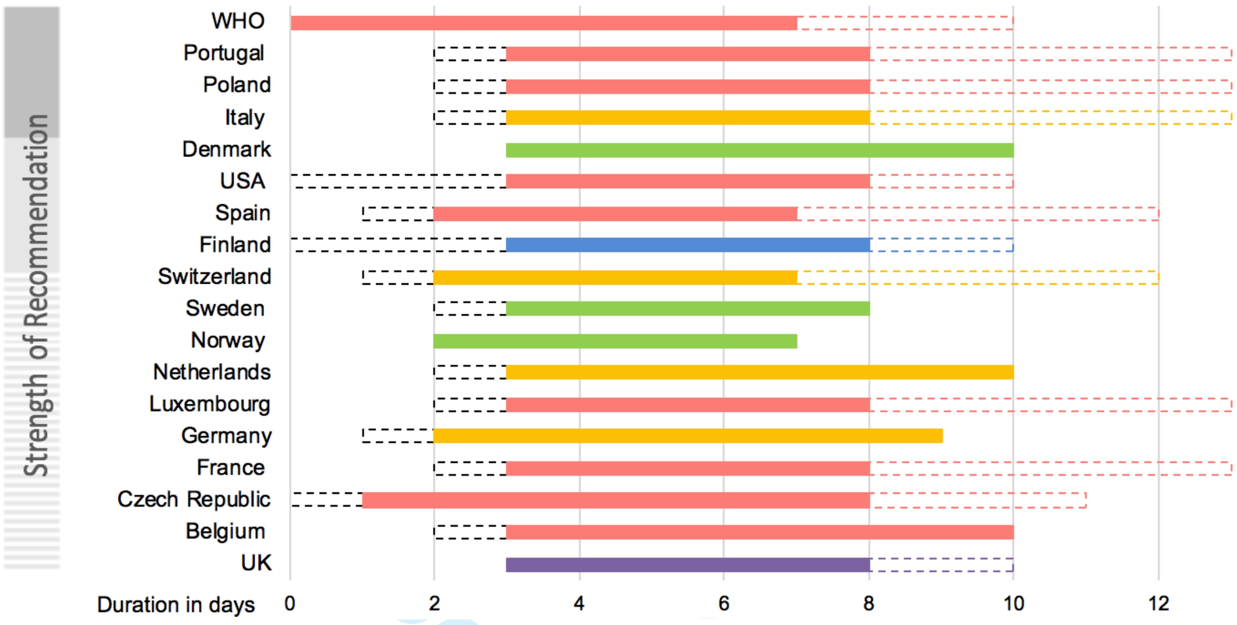

For peer review only - http://bmjopen.bmj.com/site/about/guidelines.xhtml

Clinical practice guidelines for acute otitis media in children: A systematic review and appraisal of European national guidelines

Supplementary file 1: Electronic search strategies

a) Medline via Ovid

1	exp Otitis Media/
2	otitis media.tw.
3	acute otitis media.mp.
4	exp Respiratory Tract Infections/
5	aom.mp.
6	middle ear infect*.mp.
7	guideline.mp. or exp GUIDELINE/ or exp PRACTICE GUIDELINE/
8	guide.mp.
9	manage*.mp.
10	exp Clinical Protocols/
11	1 or 2 or 3 or 4 or 5 or 6
12	7 or 8 or 9 or 10
13	11 and 12
14	limit 13 to (yr="2007 - 2017" and "all child (0 to 18 years)")

b) Embase via Ovid

1	1. exp otitis media/
2	otitis media.tw.
3	acute otitis media/
4	exp respiratory tract infection/
5	aom.mp.
6	middle ear infect*.mp
7	exp practice guideline/
8	guide.mp.
9	manage*.mp.
10	exp clinical protocol/
11	1 or 2 or 3 or 4 or 5 or 6
12	7 or 8 or 9 or 10

For peer review only

13	13. 11 and 12
14	14. limit 13 to (yr="2007 - 2017" and child)

c) Cochrane library

	Search term/strategy
1	Exp otitis media
2	Otitis media
3	Glue ear
4	midd ear adj5 (infect* or inflame*)
5	Ome or aom
6	Guideline or practice guideline
7	Management of manage*
8	Clinical protocol
9	#1 or #2 or #3 or #4 or #5
10	#6 or #7 or #8
11	#9 or #10

Results then limited to 16/11/2007-16/11/2017

d) Guidelines International Network (G-I-N)

	Search term/strategy
1	Otitis and
2	Acute Otis media

e) TRIP Medical Database

	Search term/strategy
1	Acute otitis media and
2	Otitis
3	Limit Guidelines

For peer review only

Clinical practice guidelines for acute otitis media in children: A systematic review and appraisal of European national guidelines

Supplementary File 2: Acute otitis media (AOM) guidelines in Europe

Leading author organisations (Local language)	Leading author organisations (English translation)	Country/ Region	Year published/ updated	Aimed audience	Patient age group	What system used for LoE/SoR?
Institut national d'ass urance maladie-invalidité Comité d'évaluation des pratiques médicales en matière de medicaments (INAMI)	National Institute for Health and Disability Insurance Committee for the Medical Practice on Medicinal Products Evaluation	Belgium	2016	-	Children 0-15 years	Institutional
Odbornáspolečnost praktických dětských lékařů ČLS JEP, Společnost všeobecného lékařství ČLS JEP	CzMA Society of General Practice Society	Czech Republic	2011	-	Children and Adults	None
Dansk Selskab for Almen Medicin (DSAM)	Danish Society for General Practice	Denmark	2014	General Practitioners, Parents and carers	Children 0-5 years	GRADE and Institutional
Suomalainen Lääkäriseura Duodecim	Finnish Medical Society Duodecim	Finland	2017	Primary health care	Children under school age	Institutional
Agence Française de Sécurité Sanitaire des Produits de Santé (AFSSAPS); Agence Nationale de Sécurité du Médicament et des Produits de Santé (ANSM)	French Agency for the Safety of Medicines and Health Products (AFSSAPS) (currently known as National Agency for the Safety of Medicines and Health Products (ANSM)	France	2011	-	Children >3 months of age	National Agency for Accreditation and Evaluation in Health (ANAES)

For peer review only

AWMF (Arbeitsgemeinschaft der wissenschaltlichen medizinischen Fachgesellschaften). Lead society: Deutsche Gesellschaft für Allgemeinmedizin und Familienmedizin (DEGAM)	Association of the Scientific Medical Societies in Germany. Lead society: Society of General Medicine and Family Medicine	Germany	2014	General practitioners, paediatricians, ENT surgeons, junior doctors, audiologists, and primary care workers.	Children and adult (outpatients)	Institutional (AWMF Consensus process S2k)
Health Service Executive and Royal College of Physicians Ireland	-	Ireland	2012	-	Children	None
Società Italiana di Pediatria (SIP) and Società Italiana di Otorinolaringologia Pediatrica	Italian Society of Paediatrics (SIP) and Italian Society of Pediatric Otolaryngology (SIOP)	Italy	2010	Paediatricians, ENT surgeons, general practitioners, nurses, physicians assistants	Children aged 2 months -18 years	Manual for Writing Clinical Practice Guidelines of the Programma Nazionale Linee Guida (PNLG)
Secrétariat du Conseil Scientifique - Domaine de la Santé	Scientific Council of the Ministry of Health	Luxembourg	2007	-	Children >3months of age	None
Nederlands Huisartsen Genootschap (NHG)	Dutch College of General Practitioners	Netherlands	2014	-	Children and adolescents up to 18 years of age	None

For peer review only

Antibiotikasenteret for primærmedisin (ASP)	National Antibiotics Centre for Primary Care	Norway	2016	Physicians, GPs, dentists, private practitioners, medical students	Children and adults	None
Narodowy Instytut Leków (NIL)	National Medicine Institute	Poland	2016	All specialities, including GPs, paediatricians, physicians, Respiratory physicians, ENT surgeons	Children and adults	Infectious Disease Society of America
Departamento da Qualidade na Saúde (DGS)	Department of Health Quality	Portugal	2014	Physicians of the health system	Children	European Society of Cardiology
Asociación Española de Pediatría (AEPED)	Spanish Association of Paediatrics	Spain	2012	-	Children	Infectious Disease Society of America
Läkemedelsverket	Swedish Medical Products Agency	Sweden	2010	-	Children and adults	None
Paediatric Infectious Diseases Group of Switzerland (PIGS)	-	Switzerland	2010	-	Children	None
Scottish Intercollegiate Guidelines Network (SIGN)	-	UK	2003 **Has been retracted by institution**	"All people working with children" including general practitioners (GPs), practice nurses, audiologists,	-	Institutional

				paediatricians, otolaryngologists, audiological physicians, health visitors, social workers, public health physicians, users of services and all other professions caring for children.		
American Association of Paediatrics (AAP)	-	USA	2013	Paediatricians, GPs, Emergency specialists, ENT surgeons, Nurse Practitioners, Physician's assistants	6 months- 12 years	Institutional
World Health Organisation (WHO)	-	N/A	2013	Doctors, senior nurses, senior health workers	Sick young children in low-resource setting	Institutional

For peer review only

Clinical practice guidelines for acute otitis media in children: A systematic review and appraisal of European national guidelines:

Supplementary File 3: National guidelines' Level of evidence (LoE) converted to Oxford Centre for Evidence Based Medicine (OCEBM)

**Oxford Centre for EBM Levels of Evidence**		**Belgium**	**Denmark**	**France**	**Finland**	**Italy**	**Norway**	**Poland and Spain**	**Portugal**	**UK SIGN**	**AAP**	**WHO**
1a	SR of RCTs	A/B/C	1a	1	A	I	1a	I	A	1++	A	High
1b	Individual RCT	A/B/C	1b	1	B	I/ II	1b	-	B	1++/ 1+/1-	-	-
1c	All or none	-	1c	-	-	-	-	-	-	-	X	-
2a	SR with homogeneity of cohort studies	-	2a	2	A	III	2a	II	A	2++	B/ C	High-moderate
2b	Individual cohort study	-	2b	-	B/ C	III	2a	-	B	2++/ 2+/2-	-	-
2c	Outcomes research; ecological studies	-	2c	-	C	-	-	-	-	2++/ 2+/2-	-	-
3a	SR with homogeneity of case-control studies	-	3a	3	A	IV	1b, 2a	II	A	2++/2+/2-	B/C	High-moderate
3b	Individual case-control study	-	3b	-	B/C	IV	1b, 2a	-	B	2++/2+/2-	-	-

For peer review only

4	Case series (and poor quality cohort and case-control studies	-	4	4	C	V	3	III	C	3	C/D	Low- very low
5	Expert opinion	-	5	-	D	VI	4	III	C	4	D	-
X	LOE that does not match Oxford	-	DS	-	-	-	2b	-	-	-	-	-

For peer review only

Clinical practice guidelines for acute otitis media in children: A systematic review and appraisal of European national guidelines:

Supplementary File 4: National guidelines' Strength of recommendation (SoR) converted to OCEBM SoR

Oxford Centre for EBM Strength of Recommendations		Belgium	Denmark	France	Finland	Italy	Norway	Poland and Spain	Portugal	SIGN	AAP	WHO
A	Consistent level 1 studies	-	A	A	A	A/ E	A	A/E	A	A	Strong recommendation/ Option	Strong
B	Consistent level 2 or 3 studies *or* extrapolations from level 1 studies	-	B	B	A/B	-	B	-	B	B	Strong recommendation/ Recommendation/ Option	Conditional /Weak
C	Level 4 studies *or* extrapolations from level 2 or 3 studies	-	C	C	C	-	C	-	C	C	Recommendation/Option	Conditional /Weak
D	Level 5 evidence *or* troublingly inconsistent or inconclusive studies of any level	-	D/ √	C	D	-	D	-	D	D	Option/ No recommendation	No Recommendation
X	*SOR that does not match Oxford CEBM*	1/2	DS	-		B/ C/ D		B/ C/ D	-	-		

Clinical practice guidelines for acute otitis media in children: A systematic review and appraisal of European national guidelines

Supplementary File 5: Locally defined Strength of Recommendation (SoR) for diagnostic criteria for AOM in Europe and the USA

Guideline	Diagnostic criteria included?	National SoR
Finland	Yes	A
Italy	Yes	A
Poland	Yes	A
Portugal	Yes	I
USA	Yes	Recommendation
Belgium	Yes	No grade
Czech Republic	Yes	No grade
Denmark	Yes	No grade
France	Yes	No grade
Germany	Yes	No grade
Ireland	No	No grade
Luxembourg	Yes	No grade
Netherlands	Yes	No grade
Norway	Yes	No grade
Spain	Yes	No grade
Sweden	Yes	No grade

Switzerland	No	No grade
United Kingdom	Yes	No grade
WHO	Yes	No grade

For peer review only

For peer review only

Clinical practice guidelines for acute otitis media in children: A systematic review and appraisal of European national guidelines

Supplementary File 6: Examination tools recommended by European, American and WHO guidelines for acute otitis media (AOM) in children

Country	Otoscope	Pneumatic otoscope	Tympanometry	Other	National Level of evidence/ strength of recommendation	OCEBM level of evidence/strength of recommendation
Czech Republic	+				-	-
Denmark	+	+	+		-	-
Finland	+	+	+		-/B	-/B
France	+				-	-
Germany	+	+	+		-	-
Ireland	+				-	-
Italy	+	+	+	Mirror	II/ B<br>II/ A	1b/ X<br>Ib/ A
Luxembourg	+	+		Mirror<br>Ear, Endoscope,<br>Operating microscope	-	-
Netherlands	+				-	-
Norway	+	+			-	-
Poland	+	+	+		A/ II	A/ 2a-2c
Portugal	+				-	-
Spain	+	+			-	-
Sweden	+	+	+		-	-
SIGN	+	+	+		2+/ -	2b-3b/ -
AAP	+	+	+		B/ Recommendation	2a-3a/B-C
WHO	+				-	-

No examination tools specified in Belgian and Swiss guidelines

For peer review only

Clinical practice guidelines for acute otitis media in children: A systematic review and appraisal of European national guidelines

Supplementary File 7: Investigations recommended by European, American, and WHO guidelines for acute otitis media (AOM) in children

**Country**	**Investigation**	**Circumstances**	*National LOE / SOR*	*Oxford LOE /SOR*
Czech Republic	• Culture via tympanocentesis or swab<br>• CRP	• Children <2 years of age	-	-
Finland	• Culture	• Complications of AOM<br>• Underlying immunodeficiency or significant co-morbidity<br>• Unwell patient requiring hospitalization<br>• To relieve extreme pain	-	-
France	• Culture via tympanocentesis	• Treatment failure	-	-
Germany	• Blood tests (FBC, CRP, Acute phase protein i.e. interleukin 6/BSG)<br>• Culture via tympanocentesis<br>• Ultrasound or xray	• Complications<br>• Recurrent AOM	-	-
Italy	• Blood tests (CRP, ESR, WCC)<br>• Culture via tympanocentesis<br>• Nasopharyngeal aspirate (only quantitatively)	For blood tests:<br>• For follow up<br><br>For tympanocetesis:<br>• Complications<br>• Immunocompromised<br>• Neonate	Typanocentesis: V/ B<br>NPA: IV/B<br>CT scan: IV/A	Tympanocentesis: 4 / X<br>NPA: 3a-3b/X<br>CT scan: 3a-3b/ A

	• CT scan	• Spontaneous otorrhea<br>• Sepsis<br>• Treatment failure<br>• Research purposes		
Luxembourg	• Culture via Tympanocentesis	• Aged <3 months<br>• Severe pain and convex ear on otoscopy<br>• Treatment failure	-	-
Norway	• Culture via tympanocentesis or swab of otorrhoea	• Treatment failure	-	-
Spain	• Blood tests (FBC, CRP, blood culture)<br>• Culture (Via tympanocentesis or swab of otorrhea)<br>• Lumbar puncture<br>• Skull/Temporal bone CT	• Complications<br>• Recurrent AOM<br>• Spontaneous otorrhea<br>• Treatment failure	-	-
Sweden	• Culture via tympanocentesis<br>• Nasopharyngeal swab	• Treatment failure	-	-
AAP	• Culture via tympanocentesis<br>• Nasopharyngeal aspirate	• Treatment failure	-	-

No Investigations advised: Belgium, Denmark, Ireland, Netherlands, Poland, Portugal, Switzerland, UK SIGN, WHO

For peer review only

Clinical practice guidelines for acute otitis media in children: A systematic review and appraisal of European national guidelines

Supplementary File 8: Antibiotic treatment recommended by European, American and WHO guidelines for acute otitis media (AOM) in children

Guideline	First line*	Duration of first line therapy	Second line/Treatment failure	Third line/ Allergy to first line	National LOE / SOR	Oxford LOE /SOR
Belgium	PO amoxicillin 75mg-100/kg/day in 3 divided doses	7 days	Treatment failure :<br><br>cefuroxime axetil 30-50 mg / kg in 3 doses<br><br>amoxicillin-clavulanic acid 50 / 37.5 mg / kg in 3 doses	In case of Allergy to cephalosporins , either: Co-trimoxazole (Trimethoprim 8 mg/kg/day and Sulfumethoxazole 40 mg/kg/day) in 3 divided doses<br><br>Levofloxacin 10 mg/ kg/day in 2 divided doses	For treatment choice: Expert Opinion/Weak recommendation<br><br>Duration: GRADE A - B, low recommendation)	For treatment choice: 5 /X<br><br>For duration: 1a-1b/ X
Czech Republic	PO amoxicillin 75-90mg/kg/day in 3 divided doses	7-10 days	Discusses targeted therapy to specific bacterial agents, but no empiric antibiotic for treatment failure	Allergy to beta lactams: co-trimoxazole	None	None
Denmark	PO Penicillin V 60mg/kg/day in 3 divided doses	7 days	Treatment failure<br><br><2 years of age: Amoxicillin-clavulanic acid	Penicillin allergy: clarithromycin 7.5mg.kg/dose x2 for 7 days	Level 1a-1c and 5 / Grade A and √	Level 1a-1c and 5

For peer review only

			10/2.5mg/kg/dose 8 hourly for 7 days<br><br>2-12 years of age: amoxicillin – clavulanic acid 1-/2.5mg/kg/dose 8 hourly for 7 days		For duration: No LOE/SOR	
Finland	PO Amoxicillin 40mg/kg/day 8-12 hourly<br>OR<br>PO amoxicillin-clavulanic acid 40/5.7mg/kg/day in 2-3 divided doses	5 -7 days	If vomiting: IM Ceftriaxone (one dose)	Penicillin allergy: cefaclor, cefuroximexetil, sulfa trimethoprim, azithromycin or clarithromycin	First line: C / C<br><br>(None for treatment duration)	1b, 2b-2c, and 3b-4
France	PO amoxicillin 80-90mg/kg/day in 2-3 divided doses	5 day duration if >2 years of age, 8-10 days if <2 years of age	Treatment failure: PO Amoxicillin/clavulanic acid 80mg/kg/day) AND PO amoxicillin 70mg/kg/day)<br><br>OR<br><br>IM/IV ceftriaxone 50mg/kg daily for 3 days<br><br>PO Amoxicillin/clavulanic acid = otitis conjunctivitis syndrome	Allergy to beta lactams erythromycin-sulfafuraole or cotrimoxazole<br><br>Allergy to penicillins without allergy to cephalosporins: cefpodoxime	Only duration has LOE:<br><br>Level 1 and Professional Agreement/ Grade A<br><br>Duration for child <2 years of age is Level 1/ Grade A<br>>2 years of age is professional agreement (No LOE)<br><br>3rd line treatment: Professional agreement (No LOE)	Level 1a

For peer review only

Germany	PO Amoxicillin 50mg/kg/day in 2-3 divided doses<br><br>If from country with high rates of penicillin resistance: PO amoxicillin 80-90mg/kg/day	7 days	Treatment failure: PO amoxicillin 80-90mg/kg/day<br><br>Second choice: PO cephalosporin including cefuroxime axetil (20-30mg/kg/day for 5 days),	Allergy to penicillins/cephalosporins: macrolide ie erythromycin 7 days	None	None
Ireland	Amoxicillin (no duration, no route, no frequency)	None	None	None	None	None
Italy	Mild symptoms and no otorrhea nor risk factors† :<br>PO Amoxicillin 50mg/kg/day in 2-3 divided doses<br><br>Severe symptoms, otorrhea, or risk factors for bacterial resistance<br>Amoxicillin-clavulanic acid 80-90mg/kg/day in 2-3 divided doses	Duration:<br><br>10 days <2 years of age<br>5 days >2 years of age	Treatment failure:<br>If they were treated with amoxicillin or cefaclor: amoxicillin plus clavulanic acid or cefpodoxime proxetil or cefuroxime axetil.<br><br>If they were being treated with a broad-spectrum antibiotic: intramuscular or intravenous ceftriaxone 50mg/kg once daily<br><br>Duration:<br>10 days <2 years of age or spontaneous otorrhea<br>5 days >2 years of age<br>3 days for ceftriaxone	Penicillin allergy: macrolide	First line (for both) : Level I/ Grade A<br><br>Treatment failure: Level II/ Grade B<br><br>Duration Level I/ Grade B<br><br>Allergy: VI/D	First line: Level1a- 1b/ A<br><br>Treatment failure: Level 1b/ X<br><br>Duration: Level 1a-1b /X

For peer review only

Luxembourg	Amoxycillin 80-90mg /kg/day in 3 divided doses<br><br>Alternatively if 'very severe cases'<br><br>Amoxicillin-clavulanic acid 80-90mg/kg/day in 3 divided doses	<6 years of age 10 days treatment<br><br>>6 years 5-7 days treatment	Treatment failure:<br><br>Amoxycillin/ clavulanate<br><br>Otherwise<br><br>cefuroxime axetil no dose, no frequency) or<br><br>ceftriaxone 50mg/kg/day for 3 days<br><br>or<br><br>azithromycin or clarithromycin or clindamycin	If vomiting: Ceftriaxone 50 mg / kg once daily for 3 days<br><br>Penicillin allergy: Cefuroxime 30mg/kg in two divided doses<br><br>Allergy to penicillin + cephalosporin: Azithromycin 10mg/kg/day for 6 days or clarithromycin 15mg/kg/day in two divided doses<br><br>Otherwise: sulfamethoxazole-trimethoprim 6019ng.kg of trimethoprim per day or clindamycin 30-40mg/kg in three divided doses	Not applicable	None<br>Not applicable
Netherlands	Amoxicillin 40mg/kg/day in 3 divided doses	7 days	Second line and also treatment failure:<br><br>Amoxicillin-clavulanic acid 40/10mg/kg/day in 3 divided doses for 7 days	Penicillin allergy: cotrimoxazole 36mg/kg/day in two divided doses; 5-7 days	Not applicable	Not applicable
Norway	PO phenoxymethylpenicillin 24-60mg/kg/day in 3-4 divided doses per day<br><br>In case of frequent/recurent cases:	5 days	Treatment failure:<br><br>trimetroprim sulfamethoxazole (for children)	Allergy:<br><25kg:<br>Erythromycin oral solution (ethyl succinate) 40mg/kg/day in two divided doses for 5 days<br><br>or<br>Clarithromycin (children over 6 months) 15 mg/kg/day in two divided doses for 5 days	None	None

For peer review

	Amoxicillin 21-42mg/kg/day in three divided doses			25-35kg:<br>Erythromycin enteric capsules 500mg/kg/day in two divided doses for 5 days<br>or<br>Clarithromycin 14 mg/kg/day in two divided doses for 5 days		
Poland	PO amoxicillin<br><40kg: 75-90mg/kg/day in 2 divided doses<br><br>>40kg 3000-4000mg per day in 2 divided doses	If <2 years of age, 10 days duration<br><br>If >2 years of age, 5 days duration	Treatment failure:<br>Amoxicillin/clavulanic acid<br><40kg: 70-90mg/kg/day in two divided doses<br><br>>40kg: 3000mg-4000mg per day in 2 divided doses<br><br>IVceftriaxone<br><40kg 50mg/kg/day once daily for 3 days<br><br>>40kg 1-2g/day once daily for 3 days	Allergy to amoxicillin:<br>PO Cefuroxime axetil<br>>40kg 1000mg/day in 2 divided doses for 5 days<br><40kg 30mg/kg/day in two divided doses for 5 days unless if <2 yoa then for 10 days<br><br>Allergy to amoxicillin and severe infection:<br>Ceftriaxone<br>>40kg- 1-2g IV/IM per day once daily for 3 days<br><40kg- 50mg/kg IV/IM per day once daily for 3 days<br><br>Allergy to beta lactams:<br>Clarithromycin<br><40kg 15-20mg/kg/day in two divided doses;<br>>40kg 500-1000mg/day in two divided doses	First line:<br>Level II /Grade A<br><br>First line duration: Level II /Grade B<br><br>Treatment failure:<br>Level II-III/ Grade A-B<br><br>Allergy treatment:<br>Level I-II/ Grade A-B	First line antibiotic:<br>Level 2a and 3a<br><br>First line duration:<br>Level 2a and 3a<br><br>Treatment failure: Level 2a, 3a, 4, and 5<br><br>Allergy treatment:<br>1a, 2a, 3a

For peer review only

Portugal	Amoxicillin 80-90mg/day in 2 divided doses	5 days routine or <2 years of age<br>7 days if <2 years, recurrent, failure of initial treatment<br>10 days if recurrent AOM	Treatment failure:<br><br>PO/IV Amoxicillin and clavulanic acid 80-90mg/kg/day in 2 divided doses or<br><br>PO Cefuroxime-axetil 30mg/kg/day in 2 divided doses or IV 80-100mg/kg/day in 3 dividied doses<br>7 days if <2 years<br><br>OR<br><br>IM/IV Ceftriaxone 50mg/kg/day once daily	Penicillin allergy:<br>Clarithromycin 50mg/kg/day in 2 divided doses or<br><br>Erythromycin 50mg/kg/day in 3-4 divided doses per day or<br><br>Azithromycin 10mg/kg/day once a day	First line: Level A/ Grade 1<br><br>Treatment failure: Level B/ Grade IIa<br><br>Duration:<br>7 days- Level A / Grade IIa<br><br>5 days- Level A/ Grade 1<br><br>10 days no evidence<br><br>Allergic treatment: Level C/Grade I	First line: level 1a, 2a, or 3a<br><br>Treatment failure: Level 1b, 2b, or 3b<br><br>Duration:<br>7 days- level 1a, 2a, or 3a<br><br>5 days- level 1a, 2a, or 3a<br><br>10 days no evidence<br><br>Allergic treatment Level 4-5 s
Spain	PO Amoxicillin 80-90mg/kg/day in 3 divided doses<br><br>If <6 months, severe symptoms, family history of ENT complications, previous therapeutic failure<br>Amoxicillin/clavulanic acid 80-90mg/kg/day in 3 divided doses for 7-10 days<br><br>If <2 months of age:	Routine: 5 days<br><br>Otherwise: 10 days	Treatment failure:<br><br>Amoxicillin-clavulanic acid 80-90mg/kg/day in 3 divided doses for 7-10 days or IM/IV Ceftriaxone 50mg/kg/day for 3 days	Penicillin allergy:<br>Cefuroxime axetil 30mg/kg/day in 2 divided doses<br><br>If anaphylaxis to penicillin:<br>Clarithromycin 15mg/kg/day in 2 divided doses for 7 days or azithromycin 10mg/kg once daily on first day, then 5mg/kg once daily for 4 additional days.<br>Can also give Levofloxacin<br>6 months-5 yoa: 10mg/kg every twelve hours<br><br>>5 yoa give 10mg/kg every 24 hours	First line: Level II/ Grade B<br><br>For children requiring amoxicillin-clavulanic acid as first line: Level II/ Grade B (No evidence for <2 months of age)<br><br>Treatment failure: Level III/Grade C; for	First line: 2a<br><br>For children requiring amoxicillin-clavulanic acid as first line: Level 2a-3a<br><br>Treatment failure: Level 4-5 ; Ceftraixone as used in treatment failure: Level 1a

For peer review only

	IV cefotaxime or PO/ IV amoxicillin-clavulanic acid. For PO only if no fever or no symptoms. No LOE				ceftriaxone Level I/Grade A<br><br>Allergic to penicillin/cephalosporin: Level III/Grade C	Allergy to penicillin/cephalosporin: Level 4-5
Sweden	PO Penicillin V 75 mg / kg/day in 3 divided doses<br><br>If recurrent: (new acute otitis media within a month with symptom-free intervals): Penicillin V 75mg / kg/day in 3 divided doses or<br><br>Amoxicillin 60 mg / kg/day in 3 divided doses	Routine: 5 days<br><br>Recurrent: 10 days	Treatment failure:<br><br>Amoxycillin 60mg/kg/day in 3 divided doses for ten days.	Penicillin allergy: Erythromycin 40mg/kg/day in 4 divided doses or 40mg/kg/day in 2 divided doses	Not applicable	Not applicable
Switzerland	Amoxicillin 50mg/kg/day in 2 divided doses<br><br>If risk factors or from country with high rates of penicillin resistance: Amoxicillin 80mg/kg/day in 2 divided doses	Routine: 5 days<br><br><2 years of age, previous otitis media, perforated tympanic membrane or from country with high rates of penicillin resistance : 10 days	‡Amoxicillin/clavulanic acid 80mg/kg/day in two divided doses for 10 days ‡ or<br><br>Ceftriaxone 50mg/kg daily for 1-3 days	None	Not applicable	Not applicable
UK SIGN (refers to BNF for Children)	Amoxicillin	5-7 days	Treatment failure: Amoxicillin-clavulanic acid	Penicillin allergy:	None	None

For peer review only

	Child 1-11 months of age: 125mg three times a day for 5-7 days<br><br>Child 1-4 years: 250mg three times a day for 5-7 days<br><br>(NB: dosage can be both low and high dose amoxicillin dependently on kg; for example If 1 yoa and weight <50th centile, will be given high dose; if 2 yoa and weight at 50th centile, would be given low dose.		(dose dependent on suspension available)	Clarithromycin or erythromycin ; dosage dependent on age		
USA	Amoxicillin (80–90 mg/ kg/ day in 2 divided doses<br><br>OR<br><br>If amoxicillin past 30 days, concurrent purulent conjunctivitis (otitis conjunctivitis syndrome), or history of AOM non responsive to amoxicillin:<br><br>Amoxicillin-clavulanic acid (90 mg/kg/ day amoxicillin dosage in 2 divided doses	<2 years of age: 10 days<br><br>2-5 years of age:7 days<br><br>>6 years of age 5-7 days	Treatment failure:<br><br>Amoxicillin-clavulanic acid 90mg/kg/day 12 hourly<br><br><2 years of age: 10 day course<br><br>2-5 years of age 7 day course<br><br>>6 years of age 5-7 days<br><br>OR<br><br>IM/IV Ceftriaxone 50mg daily 3 days	Penicillin allergy:<br><br>Cefdinir 14mg/kg per day in 1-2 divided doses or cefuroxime 30mg/kg per day in 2 divided doses or cefpodoxime 10mg/kg per day in 2 divided doses or ceftriaxone 50mg IM or IV per day for 1-3 days	First line : B, recommendation; first line amoxil-clav: C, recommendation<br><br>Treatment failure: B, recommendation	First line: 2a or 3a<br><br>First line amoxi clav: 2a, 3a, 4<br><br>Treatment failure: 2a, 3a

For peer review only

WHO	PO amoxicillin 80mg/kg/day in 2 divided doses<br><br>If consider pathogen sensitive, give co-trimaxazole, dose: (trimethoprim component 8mg/kg/day 12 hourly for 5 days)	7-10 days	Repeat antibiotics for another 5 days	None	Low quality evidence/ Strong recommendation	Level 4

NB Amoxicillin-clavulanic acid dose always given in terms of amoxicillin component dosage.

* Routinely to treat with low-dose amoxicillin, but for high dose amoxicillin in the following situations: Switzerland: If from area of high penicillin resistance, age <6 months, severe symptoms. UK: "If necessary." Germany: area of high penicillin resistance
† Italy risk factors: risk factors for bacterial resistance: age <3 years, day-care attendance, older siblings, recent antibiotic therapy (<1 month), no PCV-7.
‡ Switzerland: If <6 months, severe symptoms, family history of ENT complications, previous therapeutic failure, recent administration of amoxicillin, concurrent purulent conjunctivitis or otorrhoea , region of high penicillin resistance or risk factors for antibiotic resistant for PO amoxicillin-clavulanic acid as first line.

For peer review only

Clinical practice guidelines for acute otitis media in children: A systematic review and appraisal of European national guidelines

Supplementary File 9: AGREE scores for acute otits media guidelines (AOM) in European, American, and WHO guidelines

a) National AGREE scores (%)

Guideline	Domain 1	Domain 2	Domain 3	Domain 4	Domain 5	Domain 6	Mean National Score
Belgium	100	56	64	81	10	29	57
Czech Republic	36	25	4	21	0	0	14
Denmark	97	64	83	100	56	54	76
Finland	92	50	59	97	40	42	63
France	64	0	3	83	4	0	26
Germany	83	61	43	92	35	96	68
Ireland	11	3	1	56	2	0	12
Italy	100	83	63	97	40	46	72
Luxembourg	58	6	6	58	4	0	22
Netherlands	69	81	74	92	25	83	71
Norway	53	50	14	86	13	0	36
Poland	67	42	27	92	21	13	44
Portugal	44	33	18	64	33	8	33
Spain	44	28	22	83	15	29	37
Sweden	64	25	9	83	31	58	45
Switzerland	8	0	0	56	0	0	11
UK SIGN	92	92	82	92	58	29	74

For peer review only

European mean	*64*	*41*	*34*	*78*	*23*	*29*	*29*
AAP	97	67	88	89	35	54	72
WHO	94	58	80	92	60	83	78

b) AGREE scores by marker

i) HS AGREE scores

	**Domain 1**			**Domain 2**			**Domain 3**								**Domain 4**			**Domain 5**				**Domain 6**	
Criteria	**1**	**2**	**3**	**4**	**5**	**6**	**7**	**8**	**9**	**10**	**11**	**12**	**13**	**14**	**15**	**16**	**17**	**18**	**19**	**20**	**21**	**22**	**23**
Belgium	7	7	7	4	3	4	7	7	7	6	5	7	1	1	6	7	5	1	4	1	1	2	3
Czech Republic	7	1	1	6	1	1	1	1	1	1	1	1	3	1	7	4	7	1	1	1	1	1	1
Denmark	6	7	7	5	1	6	7	6	7	5	7	6	7	5	7	7	7	3	7	5	7	5	7
Finland	7	6	6	6	1	6	7	5	7	6	6	5	5	5	6	7	7	7	6	6	4	5	7
France	3	3	5	1	1	1	1	1	1	1	3	1	1	1	5	5	5	2	2	1	1	1	1
Germany	6	5	5	5	1	7	1	5	6	2	6	5	1	7	7	6	6	6	4	5	4	7	7
Ireland	1	1	1	1	1	1	1	1	1	1	2	1	1	1	5	4	6	1	1	1	2	1	1
Italy	7	7	7	7	7	7	7	6	5	6	6	6	6	4	6	7	7	6	5	5	3	5	6
Luxembourg	2	2	7	1	1	1	1	1	1	1	1	1	1	1	5	4	4	3	1	1	1	1	1
Netherlands	3	2	7	6	6	6	7	7	7	5	7	5	7	1	7	6	7	6	4	5	1	7	7
Norway	7	1	4	5	1	7	1	1	1	1	1	4	3	3	6	7	7	3	3	1	2	1	1
Poland	5	4	2	3	1	7	4	1	5	1	6	4	1	1	6	6	7	5	4	4	1	1	1
Portugal	2	1	7	1	1	4	1	1	2	1	2	3	1	4	4	3	3	3	5	2	6	1	3
Spain	4	2	2	5	2	1	1	2	3	1	4	7	1	1	6	6	5	5	2	3	1	1	5
Sweden	6	5	7	6	1	1	1	1	5	1	5	1	1	1	5	7	7	7	5	4	1	5	7
Switzerland	1	1	1	1	1	1	1	1	1	1	1	1	1	1	5	3	6	1	1	1	1	1	1

For peer review only

UK SIGN	6	5	7	6	1	7	7	6	6	7	7	6	7	5	6	6	6	7	5	5	7	3	3
AAP	7	7	7	7	1	7	6	7	6	7	6	7	6	5	5	6	7	5	6	6	2	1	7
WHO	7	7	5	5	1	7	7	5	7	5	6	5	7	6	6	5	7	6	7	7	2	6	7

ii) JED AGREE Scores

	**Domain 1**			**Domain 2**			**Domain 3**								**Domain 4**			**Domain 5**				**Domain 6**	
**Criteria**	**1**	**2**	**3**	**4**	**5**	**6**	**7**	**8**	**9**	**10**	**11**	**12**	**13**	**14**	**15**	**16**	**17**	**18**	**19**	**20**	**21**	**22**	**23**
Belgium	7	7	7	6	4	5	7	7	7	4	4	5	1	1	6	7	4	1	1	1	3	3	3
Czech Republic	5	1	4	5	1	1	1	1	1	1	1	2	2	1	4	7	7	1	1	1	1	1	1
Denmark	7	7	7	7	3	7	7	7	6	1	7	6	5	6	7	7	7	1	6	1	5	1	4
Finland	7	6	7	6	1	4	5	1	5	1	1	7	1	6	7	7	7	1	1	1	1	1	1
France	7	4	7	1	1	1	1	1	1	1	2	1	1	1	7	7	7	1	1	1	1	1	1
Germany	6	7	7	6	2	7	1	1	1	6	5	1	2	7	6	7	7	1	2	2	1	6	7
Ireland	3	2	2	1	1	2	1	1	1	1	1	1	1	1	2	2	7	1	1	1	1	1	1
Italy	7	7	7	7	1	7	4	2	4	1	4	6	3	6	7	7	7	1	4	2	1	1	3
Luxembourg	5	4	7	3	1	1	1	1	1	1	5	3	1	1	6	7	5	1	1	1	1	1	1
Netherlands	6	7	6	6	4	7	7	7	7	4	6	5	4	1	6	7	6	1	1	1	1	5	5
Norway	6	2	5	5	1	5	1	1	1	1	1	2	2	5	5	7	5	1	3	1	1	1	1
Poland	7	5	7	3	1	6	2	1	1	1	5	7	1	1	6	7	7	1	1	1	1	4	1
Portugal	4	1	7	5	1	6	1	1	1	1	2	5	1	6	5	7	7	1	1	1	5	1	1
Spain	5	4	5	6	1	1	1	1	1	1	5	6	1	1	7	7	5	1	1	1	1	1	4
Sweden	4	3	4	5	1	1	1	1	1	2	1	1	1	1	3	7	7	3	1	1	1	1	5
Switzerland	2	3	1	1	1	1	1	1	1	1	1	1	1	1	2	5	5	1	1	1	1	1	1

UK SIGN	7	7	7	7	4	7	4	3	7	6	7	6	5	6	7	7	7	2	3	1	6	1	4
AAP	6	7	7	7	1	7	5	6	7	7	7	7	6	5	6	7	7	7	1	2	1	3	6
WHO	7	7	7	6	1	7	4	1	7	7	7	7	6	6	7	7	7	5	4	4	2	4	7

For peer review only

PRISMA 2009 Checklist

Section/topic	#	Checklist item	Reported on page #
**TITLE**			
Title	1	Identify the report as a systematic review, meta-analysis, or both.	1, 2, 3
**ABSTRACT**			
Structured summary	2	Provide a structured summary including, as applicable: background; objectives; data sources; study eligibility criteria, participants, and interventions; study appraisal and synthesis methods; results; limitations; conclusions and implications of key findings; systematic review registration number.	2
**INTRODUCTION**			
Rationale	3	Describe the rationale for the review in the context of what is already known.	4
Objectives	4	Provide an explicit statement of questions being addressed with reference to participants, interventions, comparisons, outcomes, and study design (PICOS).	N/A
**METHODS**			
Protocol and registration	5	Indicate if a review protocol exists, if and where it can be accessed (e.g., Web address), and, if available, provide registration information including registration number.	No protocol
Eligibility criteria	6	Specify study characteristics (e.g., PICOS, length of follow-up) and report characteristics (e.g., years considered, language, publication status) used as criteria for eligibility, giving rationale.	4-5
Information sources	7	Describe all information sources (e.g., databases with dates of coverage, contact with study authors to identify additional studies) in the search and date last searched.	4-5
Search	8	Present full electronic search strategy for at least one database, including any limits used, such that it could be repeated.	5, Appendix 1
Study selection	9	State the process for selecting studies (i.e., screening, eligibility, included in systematic review, and, if applicable, included in the meta-analysis).	5
Data collection process	10	Describe method of data extraction from reports (e.g., piloted forms, independently, in duplicate) and any processes for obtaining and confirming data from investigators.	5
Data items	11	List and define all variables for which data were sought (e.g., PICOS, funding sources) and any assumptions and simplifications made.	N/A
Risk of bias in individual studies	12	Describe methods used for assessing risk of bias of individual studies (including specification of whether this was done at the study or outcome level), and how this information is to be used in any data synthesis.	N/A
Summary measures	13	State the principal summary measures (e.g., risk ratio, difference in means).	N/A

For peer review only

PRISMA 2009 Checklist

Synthesis of results	14	Describe the methods of handling data and combining results of studies, if done, including measures of consistency (e.g., I^2) for each meta-analysis.	6

Section/topic	#	Checklist item	Reported on page #
Risk of bias across studies	15	Specify any assessment of risk of bias that may affect the cumulative evidence (e.g., publication bias, selective reporting within studies).	N/A
Additional analyses	16	Describe methods of additional analyses (e.g., sensitivity or subgroup analyses, meta-regression), if done, indicating which were pre-specified.	6
**RESULTS**			
Study selection	17	Give numbers of studies screened, assessed for eligibility, and included in the review, with reasons for exclusions at each stage, ideally with a flow diagram.	Figure 1
Study characteristics	18	For each study, present characteristics for which data were extracted (e.g., study size, PICOS, follow-up period) and provide the citations.	7
Risk of bias within studies	19	Present data on risk of bias of each study and, if available, any outcome level assessment (see item 12).	N/A
Results of individual studies	20	For all outcomes considered (benefits or harms), present, for each study: (a) simple summary data for each intervention group (b) effect estimates and confidence intervals, ideally with a forest plot.	N/A
Synthesis of results	21	Present results of each meta-analysis done, including confidence intervals and measures of consistency.	Page 7-10
Risk of bias across studies	22	Present results of any assessment of risk of bias across studies (see Item 15).	N/A
Additional analysis	23	Give results of additional analyses, if done (e.g., sensitivity or subgroup analyses, meta-regression [see Item 16]).	N/A
**DISCUSSION**			
Summary of evidence	24	Summarize the main findings including the strength of evidence for each main outcome; consider their relevance to key groups (e.g., healthcare providers, users, and policy makers).	13
Limitations	25	Discuss limitations at study and outcome level (e.g., risk of bias), and at review-level (e.g., incomplete retrieval of identified research, reporting bias).	13
Conclusions	26	Provide a general interpretation of the results in the context of other evidence, and implications for future research.	15
**FUNDING**			
Funding	27	Describe sources of funding for the systematic review and other support (e.g., supply of data); role of funders for the systematic review.	17

From: Moher D, Liberati A, Tetzlaff J, Altman DG, The PRISMA Group (2009). Preferred Reporting Items for Systematic Reviews and Meta-Analyses: The PRISMA Statement. PLoS Med 6(7): e1000097.

For peer review only

For peer review only - http://bmjopen.bmj.com/site/about/guidelines.xhtml

PRISMA 2009 Checklist

doi:10.1371/journal.pmed1000097

For more information, visit: www.prisma-statement.org.

For peer review only
